# The Contribution of Executive Functions in Predicting Mathematical Creativity in Typical Elementary School Classes: A Twofold Role for Updating

**DOI:** 10.3390/jintelligence8020026

**Published:** 2020-06-02

**Authors:** Marije Stolte, Trinidad García, Johannes E. H. Van Luit, Bob Oranje, Evelyn H. Kroesbergen

**Affiliations:** 1 Department of Orthopedagogics: Cognitive and Motor Disabilities, Utrecht University, Heidelberglaan 1, 3584CS Utrecht, The Netherlands; j.e.h.vanluit@uu.nl; 2 Department of Psychology, University of Oviedo, Plaza Feijoo s/n, 33003 Oviedo, Spain; garciatrinidad@uniovi.es; 3 Department of Psychiatry, UMC Utrecht Brain Center, University Medical Center Utrecht, Heidelberglaan 100, 3584CX Utrecht, The Netherlands; b.oranje-2@umcutrecht.nl; 4 Behavioural Science Institute, Radboud University, PO Box 9104, 6500HE Nijmegen, The Netherlands; e.kroesbergen@pwo.ru.nl

**Keywords:** creativity, mathematics, executive functions, updating, shifting, inhibition, divergent thinking

## Abstract

The goal of the current study was to investigate the role of executive functions in mathematical creativity. The sample included 278 primary school children (ages 8–13). Two models were compared: the starting model tested whether executive functions (shifting, updating, and inhibition), domain-general creativity, and mathematical ability directly predicted mathematical creativity. The second model, which fitted the data best, included the additional assumption that updating influences mathematical creativity indirectly through mathematical ability and domain-general creativity. Updating was positively related to mathematical creativity. Additionally, updating was positively related to mathematical ability and domain-general creativity. Inhibition, shifting, domain-general creativity and mathematical ability did not have a significant contribution to either model but did positively correlate with mathematical creativity. This study reports the first empirical evidence that updating is a predictor of mathematical creativity in primary school children and demonstrates that creativity is a higher order cognitive process, activating a variety of cognitive abilities.

## 1. Introduction

It is of no doubt that creativity is important in mathematics (Sriraman 2004). For example, some mathematical questions can be answered in multiple ways or require out-of-the-box thinking (Leikin and Pitta-Pantazi 2013). Therefore, creativity should be encouraged and taught during primary school. However, teachers are unsure about how to incorporate creative exercises into their teaching methods, especially in the field of mathematics (Kaufman and Baer 2004). From this perspective, research into creativity and mathematics has increased over the last years (Singer et al. 2017), but mainly in adults or secondary school students, and much is still unknown. In particular, how executive functions are related to the domain of mathematical creativity remains unresearched. Executive functions, or the higher cognitive order functions that are necessary to reach a goal or finish a task, are important for the development and acquisition of mathematical ability. In addition, since executive functions are mainly important in novel, challenging situations in which flexibility is a key aspect (Davidson et al. 2006; Rhoades et al. 2011; Van der Sluis et al. 2007), they also seem to fulfill an important role during creative tasks (e.g., Benedek et al. 2014; Sharma and Babu 2017). Domain-general creativity and mathematical ability, in their turn, seem to promote mathematical creativity (e.g., Jeon et al. 2011; Kroesbergen and Schoevers 2017; Lin and Cho 2011; Sak and Maker 2006). To move beyond such correlational results and provide a more holistic image, the current study investigated if and how executive functions, domain-general creativity, and mathematical ability are related to mathematical creativity in a sample of 8- to 13-year-olds.

### 1.1. Domain-General Creativity and Mathematical Ability in Relation to Mathematical Creativity

Although creativity knows many definitions, a common description of the general creative process is that it is an “interaction among aptitude, process, and environment by which an individual or a group produces a perceptible product that is both novel and useful as defined within a social context” (Plucker et al. 2004, p. 90). That a more general type of creativity is required in order to be mathematically creative has been reported in several studies (Ayllón et al. 2016; Kroesbergen and Schoevers 2017; Lin and Cho 2011; Sak and Maker 2006; Schoevers et al. 2018). These studies verify that there is a domain-general part of creativity, perhaps related to insight or divergent thinking (Mumford and Gustafson 1988; Plucker 1999). However, as others have found, most variance is explained by specific abilities (Chen et al. 2006; Leikin 2014; Sawyer 2006). For instance, a very creative mathematician is not necessarily creative in another domain such as writing.

For example, Lin and Cho (2011) found that there was a direct effect of domain-general creativity on mathematical creativity, whereas variables such as motivation and environment only showed an indirect effect. Similar to the definition problem of creativity in general, the concept of mathematical creativity has also been operationalized in various ways (Mann 2006; Sriraman 2005). In the current study, mathematical creativity is used as a synonym for mathematical divergent thinking, in accordance with other work on this topic, and given the focus of the current study (e.g., Kattou et al. 2013; Leikin 2009; Stolte et al. 2018). In other words: mathematical creativity is the simultaneous activation of multiple ideas and sources of information in order to select and assemble several alternative solutions for mathematical tasks. Given the open nature of divergent thinking tasks, mathematical creativity is often associated with mathematical problem solving (Sriraman 2005). Some mathematical tasks cannot be answered with standard or pre-learned strategies. For these tasks, creativity is important because more than one answer can be correct or more than one strategy can be used to find an answer (Leikin and Pitta-Pantazi 2013). Mathematical creativity tasks are commonly scored on fluency (the number of correct answers), flexibility (the different strategies used or answer-categories), and originality (how unusual an answer is) (Sak and Maker 2006; Schoevers et al. 2018). This differentiation provides both a quantitative and a qualitative measure of creativity.

Apart from domain-general creativity, domain-specific skills and proficiency (i.e., mathematical abilities such as spatial abilities, algebraic reasoning, and number sense) (Kattou et al. 2013) are also related to mathematical creativity. In order to be creative in a domain, a person needs to have at least some familiarity with this domain to use creatively (Csikszentmihalyi 1997; Jeon et al. 2011; Sak and Maker 2006). Empirical research supports this claim (Hong and Aqui 2004; Mann 2005; Sak and Maker 2006; Schoevers et al. 2018; Stolte et al. 2018).

In comparison, the relation between domain-general creativity and mathematical ability is less straightforward. For instance, Schoevers et al. (2018) found that mathematical ability and domain-general creativity were not significantly related in a sample of fourth graders. Interestingly, their study showed that mathematical ability and domain-general creativity explained an almost similar amount of variance of mathematical creativity. They reported that creativity may not be a general construct of mathematical ability but may instead be domain-specific for mathematical creativity (Baran et al. 2011; Schoevers et al. 2018). On the other hand, a positive relation between domain-general creativity and mathematical ability has been reported in a previous study, too. In this study, with 8-, 9-, and 10-year-olds, domain-general creativity predicted a similar amount of variance in mathematical ability as updating and number sense did (Kroesbergen and Schoevers 2017).

### 1.2. Executive Functions and Mathematical Ability

A longstanding line of research shows the relation between executive functions and mathematical abilities. In such research, executive functioning is often divided into three processes: updating, shifting, and inhibition (or response inhibition) (Bull and Scerif 2001; Cragg and Gilmore 2014; Toll et al. 2011). Updating can be defined as the ability to continuously add and remove information from working memory storage as well as monitoring this process (Miyake et al. 2000). This function is important during a variety of everyday activities that involve several steps such as cooking, driving, and writing. Shifting refers to the ability to change strategies or shift from a set of rules to another; for instance, when changing between adding or multiplying numbers every other time. Response inhibition is characterized as the ability to stop an impulsive response and opt for a more appropriate response given the circumstances but also to inhibit immature strategies that are no longer optimal (e.g., when solving a mathematical assignment). Although the different executive functions are known to overlap, they each have unique properties (Miyake et al. 2000; De Ribaupierre and Lecerf 2017). Therefore, we will study them separately in the current study, whilst accounting for their possible correlation.

In mathematics, updating is required to extrapolate the individual parts from a whole and to simultaneously add new information to the mix. These are important aspects of successful mathematical reasoning (Friso-Van den Bos et al. 2013; Kroesbergen and Van Dijk 2015; Lee et al. 2012; Raghubar et al. 2010; Van der Ven et al. 2012). In addition, updating allows for the storage of intermediate results in working memory that can be manipulated to find the end-solution during a mathematical task (Van der Ven et al. 2012). Similarly, well developed shifting abilities are necessary when changing strategies during more advanced mathematical tasks (Agostino et al. 2010; Bull and Scerif 2001; Yeniad et al. 2013). However, when the shared variance with updating is accounted for, the relation between shifting and mathematics may not be present anymore (Espy et al. 2004; Van der Sluis et al. 2004; Van der Ven et al. 2012). For the third executive function, inhibition, the relation with mathematics seems comparable. Good inhibitory skills make it possible to inhibit immature strategies and irrelevant knowledge from entering working memory. Moreover, good inhibition is also necessary to stay focused on a task, and several studies find a positive relation between the two (Chiappe et al. 2000; De Beni et al. 1998; Passolunghi and Siegel 2001) but others do not support this relation (Lee et al. 2012; Van der Sluis et al. 2004; Van der Ven et al. 2012). Seemingly, updating may also be involved in inhibition and shifting, by keeping information online to manipulate in working memory (Van der Ven et al. 2012).

### 1.3. Executive Functions and (Mathematical) Creativity

Executive functions are indispensable for the flexible adaptation of skills and knowledge, especially in novel situations (Dajani and Uddin 2015). Given these characteristics, it seems plausible that they are linked to creativity as well. In the current study, we defined domain-general creativity as those creative abilities that are transferable across domains, similar to the intelligence factor g (Baer 2012; Chooi et al. 2014). Moreover, it may be that executive functioning and intelligence are related but unique concepts. For instance, one study found that only updating was a significant predictor of intelligence in young adults (Friedman et al. 2006). In a study about the unity and diversity of intelligence and executive functions in children between 7 and 9 years of age, a similar result was found (Brydges et al. 2012). Additionally, there are also indications that both inhibition and updating predict intelligence (Duan et al. 2010) and that shifting predicts intelligence (Purić and Pavlović 2012). Other studies found no such predicting effect of executive functions on intelligence (Lehto et al. 2003). This failure to find a relation was probably due to statistical decisions. In their study, Brydges et al. (2012) found a large overlap in variance between executive functioning and intelligence (between 69% and 80%). It has therefore been argued that, at least for children, what is measured during intelligence tests is not that different from the engagement of executive functioning (Brydges et al. 2012). Therefore, the current study placed its focus on executive functioning.

When creativity is required, it is important that different elements from the environment and from memory are connected even when they are not always obviously relevant at first. With this (uncommon) combination of elements, an original and creative idea can be generated. In order to do this, working memory needs to be continuously updated with new and/or different information. This leads to a broader scope of ideas and action possibilities. Therefore, a positive relation between creativity and updating can be expected (Benedek et al. 2014; Diamond 2013).

In addition, it is important to shift one’s focus from standard answers and concepts towards more novel ideas when thinking creatively. In this way, shifting also seems to be important to boost creativity by promoting flexibility (Nusbaum and Silvia 2011; Pan and Yu 2018). Surprisingly, empirical evidence for a positive relation between shifting and creativity is lacking and the only two studies reporting on this association found opposite results (Benedek et al. 2014; Pan and Yu 2018). Although (cognitive) flexibility is often mentioned as one of the cornerstones of creativity such as in the dual pathway model of creativity (Nijstad et al. 2010; Stein 1953; Torrance 1974), only one empirical study supports this claim (Pan and Yu 2018). This study by Pan and Yu (2018) only measured shifting and no other executive functions, whereas Benedek et al. (2014) took updating and inhibition into account as well. Perhaps the explanation that once several executive functions are taken into account, the explained variance from shifting is not significant anymore also applies for the relation between creativity and shifting.

Besides, good response inhibition and creativity have also been linked in the past (Benedek et al. 2014; Cassotti et al. 2016; Edl et al. 2014). Effective inhibition helps to suppress the increasing interference of previous responses in order not to persevere on initial ideas. There are also indications that good inhibition makes it difficult to move beyond less creative answers and think out-of-the-box (Burch et al. 2006; Carson et al. 2003; Scibinetti et al. 2011). However, these studies refer to an early type of inhibition, namely latent inhibition, and thus, creativity may benefit from decreased early inhibition and well-developed response inhibition. Thus, with lowered latent inhibition, a more diverse collection of stimuli is available to a person, which leads to different and perhaps more original action possibilities (Carson et al. 2003). Response inhibition, on the other hand, needs to be sufficient to stay focused on the task at hand and move beyond less creative responses. Although there are a handful of studies that report on the effect of executive functions on domain-general creativity, these studies have investigated adult samples and did not always take the shared variance of executive functions into account. In addition, empirical evidence about the relation between executive functions and mathematical creativity is missing. It is important to understand what cognitive abilities are involved during mathematical creativity in children in order for teachers to feel more confident about incorporating creativity in their math lessons (Kaufman and Baer 2004).

### 1.4. The Current Study

**Figure 1:**
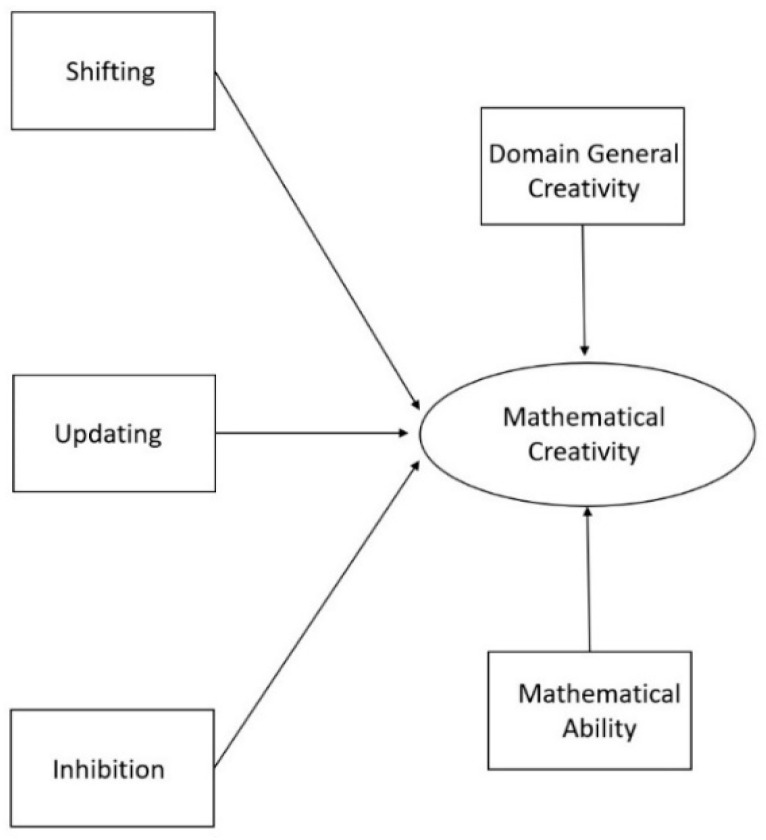
Theoretical model in which mathematical ability, domain-general creativity, shifting, updating, and inhibition all directly influence mathematical creativity. *Note.* To increase clarity, this image does not show error-terms or that we correlated the errors of the executive functions to account for their overlap.

**Figure 2:**
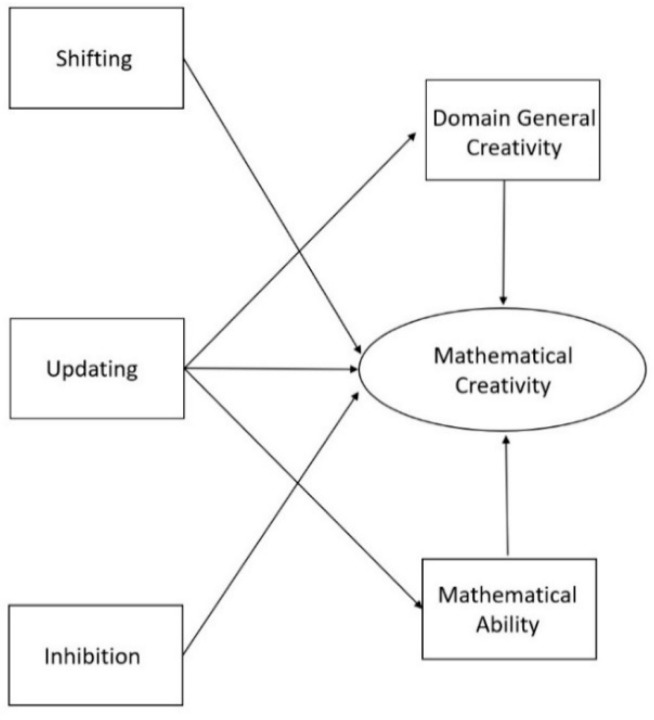
Theoretical model in which updating directly influences mathematical ability and domain-general creativity in addition to all dependent variables having a direct influence on mathematical creativity. *Note.* To increase clarity, this image does not show error-terms or that we correlated the errors of the executive functions to account for their overlap.

Even though previous studies have demonstrated that there are (mostly positive) relations between domain-general creativity, mathematical abilities, and mathematical creativity, to our knowledge, these factors have never been combined in a model with executive functions before or examined together in a sample of primary school children. Therefore, the current study aimed to create a theoretical model about the roles of the executive functions on domain-general creativity, domain-specific mathematical ability and mathematical creativity in primary school children. With this, we seek to transcend the correlational results that have been reported thus far and provide an integrated image of what underlying cognitive and behavioral factors are involved in mathematical creativity. Visual representations of our hypothesized models are presented in Figure 1 and Figure 2. Based on our discussion of the literature, we assumed that (1) domain-general creativity and mathematical ability would have a positive relation on mathematical creativity; (2) the executive function updating would have a positive relation to mathematical ability and domain-general creativity, but also to mathematical creativity; and (3) inhibition and shifting would be directly and positively related to mathematical creativity. Although there are indications that shifting and inhibition do not explain additional variances in mathematics and (general) creativity once the shared variance with updating is accounted for, no such evidence is present within the domain of mathematical creativity (Benedek et al. 2014; Espy et al. 2004; Van der Sluis et al. 2004). Indeed, given the theoretical link between shifting and creativity, we wanted to hypothesize a positive link between the two for mathematical creativity.

To investigate these hypotheses, we compared two models. The first model tested if all dependent variables (i.e., shifting, updating, inhibition, domain-general creativity, and mathematical ability) were directly related to mathematical creativity. The second model tested whether updating also had an indirect effect on mathematical creativity through its influence on mathematical ability and domain-general creativity, given that all previous studies consistently report that updating plays the biggest (and most stable) role of all executive functions in creativity and mathematics (e.g., Benedek et al. 2014; Diamond 2013; Friso-Van den Bos et al. 2013; Van der Ven et al. 2012).

## 2. Materials and Methods

Data was collected through a large-scale cross-sectional study with one measurement time point with children between the ages of 8 and 13. This study investigated the role of mathematical ability, domain-general creativity, and executive functions during mathematical creativity.

### 2.1. Participants

In the current study, 360 children participated. After excluding any cases with missing data, the final sample was composed of 278 children (*M*_age_ = 9.71, *SD*_age_ = 0.93), of which 139 (50.0%) were boys. Based on the minimum requirement of 10 cases per variable for structural equation modelling and 14 observed variables in our model, the sample was deemed sufficient for the intendent statistical method (Kline 2010). According to a teacher-questionnaire, 7 (2.5%) children that participated had autism or a related disorder and 16 (5.75%) children had an attentional disorder such as ADHD. In the Netherlands, prevalence of autism in 4–12-year olds is around 2.8% (CBS 2015). According to the DSM-5, the prevalence of ADHD is 5% in children (American Psychiatric Association 2013). Therefore, we assumed the participants to be a realistic representation of children in Dutch primary school education. Participants were recruited from 21 classes of 9 regular primary schools, situated in the Netherlands. All schools were located in the central part of the Netherlands. Four schools were situated in an urban area (i.e., city with more than 50,000 inhabitants) and five schools were situated in more rural areas. Six of the schools had classes with 20 children or more, whereas the others worked with a system of smaller classes. Children were included in the study if at least one of the parents gave active informed consent. Prior to data collection, the study had been approved by the Ethics Committee of the Faculty of Social and Behavioral Science of Utrecht University in 2016 (FERB16-112).

### 2.2. Procedure

Over the course of two days, participating primary school children were administered a test battery containing several tasks that measured mathematical ability, creativity, executive functioning, and intelligence. On both days, the session was roughly one hour. Testing commenced at the participating primary schools. Children were, therefore, in a familiar and safe environment. Most classes completed the tests on two consecutive days. If this was not possible, the two test days were, at most, nine days apart. On day one, participants completed individual paper-and-pencil tasks in their own classroom. During day one, the participants sat in a test setup and completed the domain-general creativity task amongst several other tasks not relevant to the current study. On day two, participants made one more paper-and-pencil task in the classroom. This task measured mathematical creativity. In addition, the executive functioning tasks were administered individually in a separate quiet room in small groups of maximal six children. There were three executive functioning tasks, two to measure updating and one that measured shifting and inhibition. All executive function tasks were computerized in order to measure reaction time and accuracy. All tests were administered and supervised by at least one test leader. Test leaders were trained prior to data collection by their supervisor and used a protocol during data collection to ensure standard test instructions across classes, schools, and test leaders.

### 2.3. Materials

#### 2.3.1. Inhibition

**Figure 3:**
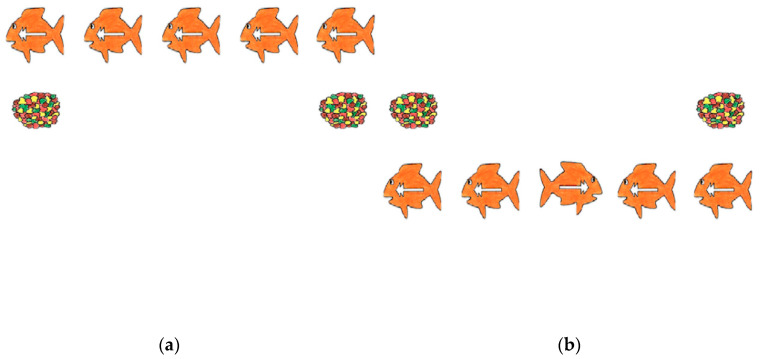
(**a**) An example of a congruent trial during the Fish Game and (**b**) an example of an incongruent trial during the Fish Game.

The Fish Game (Stolte et al. 2018) measured inhibition, which is an adapted version of the classical Flanker task, where the middle target has to be identified amongst distractors. In the Fish Game (of which Figure 3 shows a visual representation), the middle target is flanked by four identical targets that can be identical to the middle target (i.e., congruent trials) or be the mirror image of the middle target (i.e., incongruent trials). The Fish Game also contained so called neutral trials, in which only one stimulus was presented without distractors. The task started with five practice trials, in which the participant received feedback on the accuracy of the responses. The practice trials were followed with a testing block that contained 12 congruent trials, 12 incongruent trials, and 12 neutral trials. Stimulus presentation was at random and participants had 2000 ms to respond. Stimuli were presented either at the top or the bottom of the screen. To measure inhibition, average reaction time during incongruent trials was used. Therefore, this task is a reversed indicator of inhibition. The inhibition block of the Fish Game has good internal consistency as assessed in the current study (neutral trials α = 0.84; congruent α = 0.87; incongruent α = 0.88).

#### 2.3.2. Shifting

**Figure 4:**
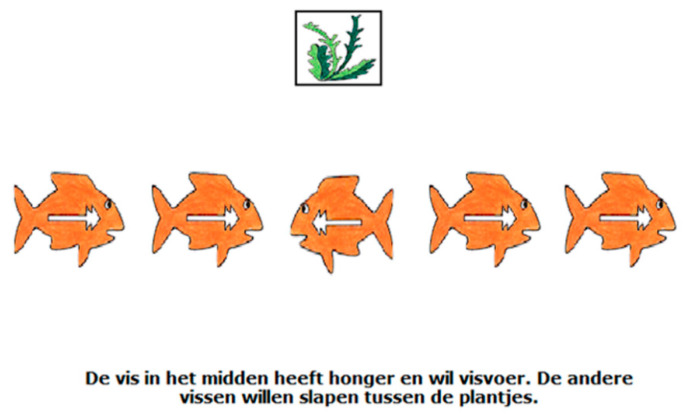
An example of a shifting trial during the Fish Game. Translation of text in the figure: “The fish in the middle is still hungry and wants fish food. The other fishes want to go to sleep between the plants.”

The second block of the Fish Game measured the executive function shifting. During this part of the task, the child has to keep shifting between two strategies. The child is instructed that when they see an image of a plant (see Figure 4 for an example), they need to respond to the direction that the fish on the outside are swimming in. This is because the fish on the outside are tired and want to go to sleep between the plants. However, when an image of fish food is presented, the child is instructed to respond to the middle target, as they are used to from the first block of the Fish Game that measured inhibition. The story behind this is that the middle fish is still hungry and, therefore, wants to go to the fish food. This block of the game contains 22 shift trials (the trials with an image of a plant) and 18 non-shift trials (the trials with an image of fish food). Participants had 2500 ms to respond to each trial and trials were presented in a fixed order. The shifting block of the Fish Game has good internal consistency as assessed in the current study (shifting trials’ Cronbach’s α = 0.94; non-shift trials’ Cronbach’s α = 0.99). We measured shifting ability by recording the average reaction time in milliseconds on the shift trials. Therefore, this variable is a reversed indicator of shifting.

#### 2.3.3. Updating

Two tasks were administered to measure updating (Van de Weijer-Bergsma et al. 2016). Verbal updating was measured with the Monkey Game. In this task, the child is instructed to remember and recall a sequence of words in the reversed order. During the task, the child will hear a sequence of spoken words, for example “fire, coat, cat.” Then, the child is instructed to remember this sequence in reverse, which, in this case would be “cat, coat, fire.” The Monkey Game measures this by letting the child click on the correct words in the correct order. All words are presented in a 3 × 3 matrix. The task contains five levels with four trials. In the first level, the child has to remember and reverse two words. In the last level, the child has to remember and reverse a sequence of five words. Internal consistency of the Monkey Game was found to be “acceptable” to “good” (Cronbach’s α between 0.78 and 0.89). Additionally, concurrent validity was also good (ρ between 0.51 and 0.59) (Van de Weijer-Bergsma et al. 2015a).

To measure visuo-spatial updating, participants completed the Lion Game (Van de Weijer-Bergsma et al. 2015b). The children were asked to remember where they saw the last lion with a specific color. The task consisted of a 4 × 4 matrix. Every square contained a bush, behind which a colored lion could appear. Each trial, eight lions were presented on the screen, one after the other. Every lion was visible for 2000 ms. Lions could be green, yellow, purple, blue, or red. After eight lions were presented, the child was instructed to click in the matrix where they had seen the last lion with a specific color. The task consisted of 20 trials which were equally divided over five levels. The task increases in difficulty because the number of colored lions that should be remembered increased with every level, starting at two colors and ending with all five colors. The Lion Game has good internal consistency (Cronbach’s α between 0.86 and 0.90), good concurrent and predictive validity, and a satisfactory test-retest reliability (ρ = 0.71) (Van de Weijer-Bergsma et al. 2015b).

For both the Monkey and the Lion Game, the proportion of items correct is recorded, which leads to a score between 0 and 1. The current study created a composite score of updating by first standardizing the outcomes for the Monkey and the Lion Game to account for possible differences in mean and standard deviation between the tasks. Hereafter, the standardized scores were added and subsequently averaged to create the new updating variable.

#### 2.3.4. Mathematical Creativity

We measured mathematical creativity with an adapted version of the Mathematical Creativity Test developed by Kattou et al. (2013) (see Schoevers et al. 2018). This task contained five open-ended mathematical questions about geometry that could be answered in multiple ways. Participants are instructed to think of as many answers to each of the mathematical questions as they can. In our adapted and translated version, we aimed to measure the construct of mathematical creativity instead of geometric creativity. To achieve this, we used three questions from the original task and included a similar question that Hershikovitz et al. (2009) used in their research. This question is about dividing a square pie in such a way that four people would get the same amount. This task had the following instruction: “Four children [names given] have to share a square cake fairly. How will they cut the cake?” In addition to this instruction, we included a sentence instructing participants to think of as many different solutions to cutting the cake as they could. Answers were scored on fluency, flexibility (maximum score = 22), and originality (maximum score = 1 for each question). Similar to Leikin and Lev (2013), fluency was operationalized as the sum score of correct answers. Flexibility was operationalized as the number of categories the correct answers could be placed in. Originality was scored by comparing the answer of a participant to a reference group. Previous research reported that the Mathematical Creativity Test had an acceptable internal consistency (Cronbach’s α = 78) (Kattou et al. 2013). An exploratory factor analysis was done to investigate if these questions measured the same latent construct.

#### 2.3.5. Mathematical Ability

The Cito test is a Dutch ability test used by most Dutch primary schools in grade 1 through 5 to monitor spelling, vocabulary, reading comprehension, and mathematical development (Janssen et al. 2007). The test contains multiple choice questions and is used to advise children on the most appropriate level of higher education after primary school and is administered twice a year by teachers. We requested the most recent ability scores of the mathematical part of the Cito test from the participating schools to obtain a measure of participants’ standard mathematical abilities. The mathematical part of the Cito has different subtests to measure different types of mathematical ability (e.g., arithmetic, measuring, fractions, percentages, and proportions), adjusted for the level of mathematical ability in each grade. The sum of the subtests leads to a final ability score in a domain, in this case, mathematical ability. The ability scores of the Cito test have a good reliability (Cronbach’s α between 0.91 and 0.94 for grades 3 and 5) (Janssen et al. 2010). Since the ability scores differ between grades and schools, in different versions of the Cito test, we made standard scores of all values to be able to compare them.

#### 2.3.6. Domain-General Creativity

The Test for Creativity Thinking Drawing Production (TCT-DP) is a measure of general creativity (Kālis et al. 2014; Urban 2004). We selected this measure as our variable for domain-general creativity since it supersedes the dichotomy of convergent and divergent creativity and incorporates non-cognitive aspects of creativity into the task (Urban 2004). During administration of the TCT-DP, each participant receives a piece of paper that contains six figural fragments. A test leader than instructs participants that a painter started with this painting and that the participant will now have to finish it however they see fit. Participants have to try to finish the drawing in 15 min (or less). The end product is scored on 14 creativity aspects such as new elements and humor. Time is taken into account if the score of the first 13 creativity aspects is at or above 25 points and a maximum score is calculated. The points gained on the 14 aspects are summed to a total score, which can reach a maximum of 72 points. The TCT-DP has a high differential reliability (*χ*^2^ = 33.45, C_(corr.)_ = 0.92) (Urban 2004) and good interrater reliability in the current study (Cronbach’s α *=* 0.96).

### 2.4. Analyses

The relationship between variables were examined by performing Spearman correlations, as all the variables were found to have a non-normal distribution (based on the Shapiro-Wilks test of normality). Before performing the path analyses, an exploratory factor analysis (EFA) for mathematical creativity was conducted in order to test if one latent construct could be created. Hereafter, path analyses were performed to test our theoretical models. This was done twice; with and without the proposed mediation effects (Figure 1 and Figure 2). In order to formally test our mediation model, we performed a bootstrap with 100 samples. Robust Maximum Likelihood was used to assess the model since our data failed to meet the assumption of normally distributed data. The degree of fit was based on the Chi-square (*χ*^2^) and degrees of freedom (*df*) ratio, the comparative fit index (CFI), the Tucker Lewis index (TLI), and the root mean square error of approximation (RMSEA) and standardized root mean square residual (SRMR). The model fit was considered to be good when CFI ≥ 0.95, TLI ≥ 0.95, and RMSEA ≤ 0.06, SRMR ≤ 0.08 and *χ*^2^/*df* < 3 (Schreiber et al. 2006). The analyses were carried out with the sample as a whole, leaving age and gender out of the equation to reduce complexity and in order to have a sufficient sample size for structural equation modelling. The statistical programs, SPSS 24 and the SPSS add-on for structural equation modelling AMOS 24, were used. The Supplementary Materials contains the data package of the current study. 

## 3. Results

Before testing our hypothesized models, we investigated the correlations between the variables alongside the descriptive statistics, which can be viewed in Table 1, and employed EFA to extract a latent factor of mathematical creativity.

Mathematical creativity showed significant positive correlations with domain-general creativity, mathematical ability, updating, shifting and inhibition. Moreover, in line with our expectations, domain-general creativity and mathematical ability also had positive correlations with updating.

EFA was performed to investigate if the four questions from the mathematical creativity task measured the constructs of fluency, flexibility, and originality in a similar way. We chose this method because there is no data available on how the three questions from the original mathematical creativity task are related to the question from Hershikovitz et al. (2009) that we included. The results showed that when all four questions were added to the analyses, there was no significant increase in the amount of explained variance (73.16% versus 74.07%) compared to when the three original questions from Kattou et al. (2013) were added. In addition, in the EFA containing three factors, all correlations between the scoring components (i.e., fluency, flexibility, and originality) were significant, whereas this was not the case when all four questions were included. Therefore, we concluded it was best to exclude question number 2 from Hershikovitz et al. (2009) from further analyses. In other words, it seems that the three separate questions from the mathematical creativity task each measure a unique part of mathematical creativity. In question 1, for example, creative geometry is examined; question 3 is about creating an equation, and question 4 is about number sense.

**Figure 5:**
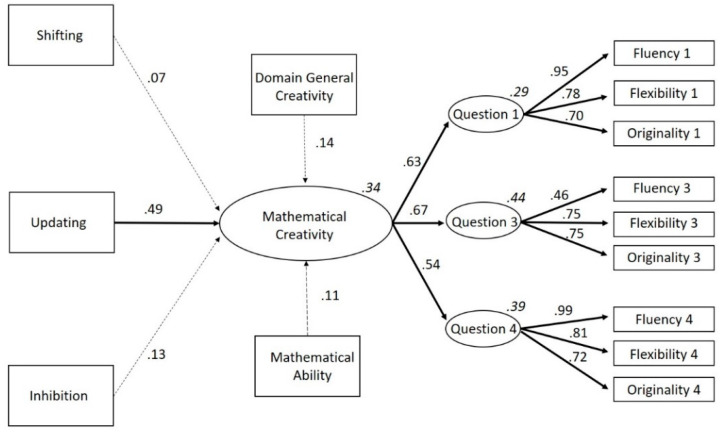
Standardized factor loadings of the starting model, with only direct effects on mathematical creativity (Model 1). Bold arrows signify a significant relation, striped arrows signify an insignificant relation. R^2^ of the endogenous variables is added in cursive above its rectangle. *Note.* To increase clarity, this image does not show error-terms or that we correlated the errors of the executive functions or the error covariances between fluency, flexibility, and originality to account for their overlap.

As for the two different models tested, Model 1 (the starting model, with 57 estimated parameters, depicted in Figure 1) showed a good fit to the empirical data (CFI = 0.972, TLI = 0.958, *χ*^2^ = 97.32, *df* = 62, *χ*^2^/*df* = 1.57, *p* = 0.003, RMSEA = 0.045 (90% CI: 0.027–0.062), SRMR = 0.059). A full representation of the model can be viewed in Figure 5 The starting model demonstrated a positive relation between shifting, updating, inhibition, domain-general creativity, mathematical ability and mathematical creativity. Unlike the significant Spearman correlations, the effects of inhibition (Standard Error = 0.000; Critical Ratio = −1.563; *p* = 0.118), shifting (Standard Error = 0.000; Critical Ratio = −0.862; *p* = 0.389), domain-general creativity (Standard Error = 0.012; Critical Ratio = 1.875; *p* = 0.061), and mathematical ability (Standard Error = 0.061; Critical Ratio = 1.461; *p* = 0.144) on mathematical creativity were not statistically significant. The effect of updating on mathematical creativity was statistically significant (Standard Error = 0.094; Critical Ratio = 5.314; *p* < 0.001) (see Figure 5).

**Figure 6:**
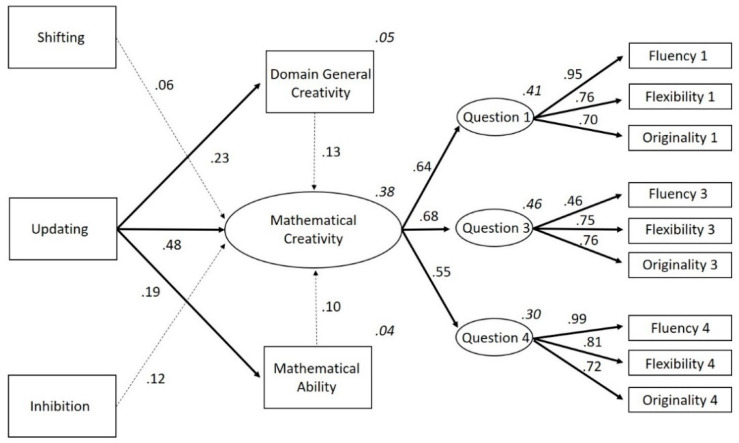
Standardized factor loadings of the second model with indirect and direct effects of updating on mathematical creativity (Model 2). Bold arrows signify a significant relation, striped arrows signify an insignificant relation. R^2^ of the endogenous variables is added in cursive above its rectangle. *Note.* To increase clarity, this image does not show error-terms or that we correlated the errors of the executive functions or the error covariances between fluency, flexibility, and originality to account for their overlap.

Next, we tested Model 2, with direct associations between updating on the one hand and mathematical ability, domain-general creativity and mathematical creativity on the other (as originally depicted in Figure 2). This model had 59 estimated parameters which met the fit requirements (CFI = 0.990, TLI = 0.985, *χ*^2^ = 72.64, *df* = 60, *χ*^2^/*df* = 1.21, *p* = 0.13, RMSEA = 0.028 (90% CI: 0.000–0.047), SRMR = 0.043). For a full visual representation of the results, see Figure 6 This model revealed a positive influence of updating on mathematical ability and mathematical creativity. Furthermore, shifting, updating, inhibition, mathematical ability, and domain-general creativity also positively influenced mathematical creativity. The effects of inhibition (Standard Error = 0.000; Critical Ratio = −1.564; *p* = 0.118), shifting (Standard Error = 0.000; Critical Ratio = −0.878; *p* = 0.380), mathematical ability (Standard Error = 0.061; Critical Ratio = 1.476; *p* = 0.140), and domain-general creativity (Standard Error = 0.006; Critical Ratio = 1.887; *p* = 0.0.59) on mathematical creativity were not statistically significant. All other paths were statistically significant (see Figure 6 and Table 2). Thus, although the total mediation effect of updating on mathematical creativity through mathematical ability or domain-general creativity was not significant, we can still conclude partial mediation is present (MacKinnon et al. 2002; Shrout and Bolger 2002; Zhao et al. 2010).

This second model had a lower AIC and BCC (AIC = 19064, BCC = 197.40) compared to the first model (AIC = 211.32, BCC = 217.84), which indicates that it fits the data better. For a complete overview of statistics of the second model, see Table 2.

Table 2 shows the coefficients of the relations in the structural and measurement models of the second model, as well as their corresponding estimation errors, critical ratio, and associated significance. The measurement model represents the relations between latent variables or composite variables while the structural model tests all the hypothetical dependencies based on path analysis (Kline 2010).

## 4. Discussion

The aim of the current study was to provide an overview of the relation between executive functions, domain-general creativity and mathematical ability on mathematical creativity. To this end, we tested and compared two models. The first model consisted of only direct relations of mathematical ability, domain-general creativity, and executive functions (shifting, updating, and inhibition) on mathematical creativity. The second model consisted of all direct relations including indirect effects of updating through its influence on domain-general creativity and mathematical ability. Based on the correlational analyses, we found significant associations between mathematical creativity and updating, shifting, inhibition, mathematical ability, and domain-general creativity. Furthermore, our results revealed that a model in which mathematical ability and domain-general creativity act as partial mediators between updating and mathematical creativity, and where updating also has a direct relation to mathematical creativity, fitted the data best. More specifically, there was a positive relation between updating and mathematical ability, domain-general creativity, and mathematical creativity. Additionally, mathematical creativity was positively related to updating directly as well.

Concerning the relation between updating and mathematical creativity, we found most support for the model in which updating influenced mathematical creativity directly and indirectly through its positive association with mathematical ability and domain-general creativity. Although this has not been studied before in the domain of mathematical creativity or in children, this result was not unexpected because it is in line with the known relation between general creativity and updating in adults (Diamond 2013; Benedek et al. 2014) and with the relation between mathematical ability and updating in children (e.g., Friso-Van den Bos et al. 2013; Van der Ven et al. 2012). Thus, since mathematical creativity requires both domain-general creativity and mathematical ability, it can be argued that updating exerts its influence on mathematical creativity in direct and indirect ways. That is, although the total effect of the mediator on mathematical creativity was not significant, indirect mediation was still present since the path between updating and domain-general creativity, and updating and mathematical ability was significant (MacKinnon et al. 2002; Shrout and Bolger 2002; Zhao et al. 2010). Updating plays a vital role in both processes because it allows for the storage of intermediate results or ideas. Mathematical tasks and creativity tasks are often comprised of a multi-step process in which updating is therefore necessary to move from one step to the next. To apply one’s abilities creatively, preexisting knowledge and skills should be activated, and working memory continuously updated to come to creative solutions (Benedek et al. 2014).

Similarly, general creative abilities are transferable to specific domains and therefore, requested during mathematical creativity, too (Jeon et al. 2011). Thus, well developed general creative abilities might make it possible to use one’s mathematical abilities creatively during a divergent thinking task (Bahar and Maker 2011; Sak and Maker 2006). However, it is not possible to make a prediction about the direction of this relation at this point and it may be that mathematical ability influences domain- general creativity, too (Kattou et al. 2013). Furthermore, one can question whether our current task to assess mathematical creativity did indeed measure this construct or if the strong connection between executive functions and mathematical creativity reflect the predicted positive link between novelty and executive function as well (Davidson et al. 2006; Rhoades et al. 2011; Van der Sluis et al. 2007). In a regular classroom environment, mathematics and divergent thinking are seldom combined. Therefore, the mathematical creativity task that we used may have made an appeal to the executive functions of the participating children because of their unfamiliarity with such tasks and because of the divergent thinking aspect, which explains the strength of the correlation that we found. Additionally, since the positive correlation between mathematical abilities and mathematical creativity was no longer significant after executive functions were taken into account, there appears to be a common variance between the two. We recommend future studies to take other (domain-general) cognitive factors into account, such as intelligence, processing speed, or motivational factors (Brydges et al. 2012; Clark et al. 2014; Duan et al. 2010; Tella 2007; Moenikia and Zahed-Babelan 2010).

Although this study replicated the positive relation between updating, domain-general and domain-specific aspects of creativity, the role of response inhibition and shifting on creativity and mathematics is still debatable. Regarding inhibition, we found a significant relation between inhibition and mathematical creativity in the correlational analysis but in the path model there was no such effect. This may be because there is overlap of response inhibition with updating and shifting (Miyake et al. 2000). Therefore, the explained variance of inhibition may have gone through the other two executive functions instead. Since the executive functions are still developing in our included age range, it is possible that the three functions are not fully distinct factors yet (Huizinga et al. 2006; Van der Sluis et al. 2007; Van der Ven et al. 2012). Since making different age groups within the current sample would have led to too small samples to draw definite conclusions, we recommend future research to be done with a larger sample size to replicate and expand the current results with more specific information about the contribution of age and gender to the model.

In the correlational analysis, the current study found that response inhibition had a small positive, albeit nonsignificant, predictive value on domain-general creativity but a significant positive relation to mathematical creativity. This deviates from previous results that investigated domain-general creativity (Benedek et al. 2014; Cassotti et al. 2016; Edl et al. 2014). This suggests that the relation between inhibition and creative activities may be task dependent, and something similar may have played a role in our other variables of interest as well. This is further corroborated in the study by White and Shah (2006). This provides support that inhibition can either help or harm the creative process, depending on the specific measure of creativity. Previous literature indicated that creativity and good response inhibition were connected by the suppression of interferences from dominant responses (e.g., Benedek et al. 2012a; Groborz and Necka 2003) but negative correlations were found when no such interference was present (Vartanian et al. 2007). For instance, the TCT-DP may require less response inhibition because of a lack of interference during the task. When this task is first presented, divergent thinking is important to stipulate the different options of finishing the painting, and response inhibition is necessary to delay making a decision what to draw until you have reviewed several ideas. After assessing all options, convergence is necessary to choose which option to draw, which requires good response inhibition (White and Shah 2006). However, since the TCT-DP is not a particularly time sensitive or complex task, with no ‘incorrect’ answers, the executive functions are probably less engaged (Miyake et al. 2000). Since no competing concepts or ideas are present anymore at the stage of drawing, response inhibition is probably less important.

However, in a divergent task such as our mathematical creativity task, it seems plausible that some response inhibition is needed to overcome interference of common ideas. To generate more creative ideas, it is necessary to activate concepts that are more distantly associated with a task or problem, which is related to earlier forms of inhibition and attention (Benedek et al. 2012b; Carson et al. 2003; Friedman and Miyake 2004). When stated this way, perhaps it is not so much efficient response inhibition that is important during mathematical creativity but efficient updating skills. At first, the most common and closely related concepts are activated in working memory. Hereafter, updating is required to facilitate the process of gating less obvious information into working memory as well (Benedek et al. 2014; Diamond 2013). These strong and weakly related concepts can be combined to form novel and creative ideas (Mednick 1962). As such, it is not necessarily the inhibition of irrelevant information that is important but the continuous updating of the information in the working memory, in order to create new combinations, that is important for creativity.

Regarding the relation between shifting and mathematical creativity, the current study found no support that well developed shifting abilities are linked to mathematical creativity once the overlap with updating was accounted for. Shifting, as well as other executive functions, are used during perspective taking. That is, processes such as perspective taking first require inhibition of the old perspective, making space for new ideas (i.e., the process of updating working memory) to come to a new perspective or idea (Davidson et al. 2006). Therefore, it is often cited that (cognitive) flexibility or shifting are paramount in creativity and mathematics (Bailey et al. 2007; Bull and Scerif 2001; Nijstad et al. 2010; Yeniad et al. 2013). Within the field of mathematics, there is research supporting this finding (i.e., Clark et al. 2010; Mayes et al. 2009) although not all studies find such clear results when other executive functions are added (Espy et al. 2004; Toll et al. 2011). However, for the field of mathematical creativity, such empirical evidence is missing. One study on shifting and creativity did find a relation between creativity and shifting (Pan and Yu 2018) but the only other study that investigated this relation did not (Benedek et al. 2014). Providing an explanation for this difference in results is difficult. Perhaps the difference in age of participants, the complexity of the task, or the type of creativity task (domain-general versus mathematical creativity) was a factor or the difference in the measurement of shifting (difference score or reaction time of shift-trials). More likely, however, the difference in whether or not shared variance with updating was taken into account explains these discrepancies in results (Van der Ven et al. 2012). The current study further strengthens the idea that shifting and inhibition are ancillary to updating during creative and mathematical tasks.

## 5. Conclusions and Future Directions

The current study provided the first theoretical model that included the roles of the executive functions of updating, shifting and inhibition, mathematical ability, and domain-general creativity, on mathematical creativity in children. This contributed to our understanding of the complex underlying factors to mathematical creativity and further strengthens the idea that creativity/divergent thinking is a top-down process (Razumnikova 2007; Zabelina et al. 2016). The substantive sample size allowed for employing structural equation modelling which made it possible to test the fit of several models to the data and to compare these models against each other. In addition, from a theoretical perspective, the graphical representation of the relations between variables increases our understanding of set connections and it provides a means to examine the impact of direct as well as indirect relations within the same analysis.

Our study implies that updating is associated with mathematical creative performance in a direct and in a smaller capacity, as well as in an indirect manner because it positively predicted domain-general creativity and mathematical ability as well. Although the current study contributed to our understanding of mathematical creativity, it is not without its limitations. First, caution should be taken when generalizing these results as it seems that results are task dependent. In the current study, we used a mathematical divergent thinking task, while convergent thinking was not measured separately. Therefore, conclusions are limited to divergence. Future research should take both forms of creativity into account to provide a more complete picture. Second, it is recommended to use more than one measure for domain-general creativity in the future to better capture the entire construct of general creative abilities (Cropley 2010). Although the TCT-DP is widely used to measure creativity thinking and creative potential in a culturally independent way, domain-specific abilities are involved (Kālis et al. 2014; Urban 2004). Third, since the current study had one measurement time point, it is not possible to say something about the causal relationships between variables. Therefore, it would be beneficial to carry out future research with a longitudinal design to examine if the implied causal relations described here can be confirmed. Additionally, our sample was restricted in terms of age. This makes any generalization of our results to other age groups difficult, especially since the ability of inhibiting one’s irrelevant thoughts and responses is still developing until the age of 11, shifting abilities until the age of 12, and updating, even until 18 years of age (Carlson et al. 2004; Gathercole et al. 2004; Huizinga et al. 2006; Huizinga and Van der Molen 2007).

Despite these limitations the current study provided a first look at the underlying cognitive factors of mathematical creativity in primary school children. These results can have important implications for how primary school teachers can promote (mathematical) creativity. While the effectiveness of training programs for executive functions is up for discussion (Karbach and Unger 2014), insight into a person’s strengths and weaknesses can serve an important purpose for psychoeducation, for example. By creating awareness about the role of, and perhaps providing training in executive functions such as updating abilities, creativity can be promoted in domain-general and domain-specific ways.

## Figures and Tables

**Table 1:** Descriptive statistics and Spearman correlations of the studied variables.

	Mean	SD	Skew	Kurtosis	1	2	3	4	5	6
1. D-G Creativity	20.33	9.58	0.63	−0.44	-					
2. Math Ability	0.01	1.00	−1.20	4.78	−0.003	-				
3. Updating	0.04	0.83	−0.75	0.73	0.209 ***	0.153 *	-			
4. Shifting	1386.83	236.50	−0.48	0.77	0.072	0.097	0.124 *	-		
5. Inhibition	790.08	151.84	1.12	1.65	0.101	−0.038	0.354 ***	0.416 ***	-	
6. MC (Factor)	0.000	0.69	0.33	−0.23	0.198 **	0.202 **	0.429 ***	0.169 **	0.281 ***	-

^1^ D-G = Domain-General; Math Ability = Mathematical Ability; MC = Mathematical Creativity. ^2^ For shifting and inhibition, scores were reversed so that higher values indicated faster reaction times. ^3^ *** *p* < 0.001; ** *p* < 0.01; * *p* < 0.05.

**Table 2:** Results of testing the second model with the direct and indirect effect of updating on mathematical creativity (structural and measurement models).

			Standardized Coefficients	SE	CR	P
Structural Model						
D-G Creativity	→	Updating	0.226	0.672	3.865	***
Math Ability	→	Updating	0.189	0.071	3.212	0.001
Math Creativity	→	Math Ability	0.102	0.061	1.476	0.140
Math Creativity	→	D-G Creativity	0.132	0.006	1.887	0.059
Math Creativity	→	Inhibition	0.121	0.000	−1.564	0.118
Math Creativity	→	Shifting	0.063	0.000	−0.878	0.380
Math Creativity	→	Updating	0.475	0.090	5.521	***
Measurement Model						
Question 1	→	Math Creativity	0.638	-	-	-
Question 3	→	Math Creativity	0.677	0.645	4.767	***
Question 4	→	Math Creativity	0.549	0.165	5.910	***
MC 1 fluency	→	Question 1	0.950	-	-	-
MC 1 flexibility	→	Question 1	0.760	0.030	13.842	***
MC 1 originality	→	Question 1	0.699	0.014	12.603	***
MC 3 fluency	→	Question 3	0.460	-	-	-
MC 3 flexibility	→	Question 3	0.754	0.025	6.428	***
MC 3 originality	→	Question 3	0.755	0.007	6.438	***
MC 4 fluency	→	Question 4	0.987	-	-	-
MC 4 flexibility	→	Question 4	0.809	0.030	17.092	***
MC 4 originality	→	Question 4	0.722	0.009	14.363	***

^1^ D-G = Domain-General; Math Ability = Mathematical Ability; MC = Mathematical Creativity Question; Standardized Coefficients = Standardized Regression Weights; SE = Standardized Errors; CR = Critical Ratio; p = Probability; Structural Model = relation between the independent and the dependent variables in the model; Measurement Model = relation between the latent variables and the observed variables in the model. ^2^ For shifting and inhibition, scores were reversed so that higher values indicated faster reaction times. ^3^ *** *p* < 0.001.

## Supplementary Materials

The data package of the current study is available online at https://www.mdpi.com/2079-3200/8/2/26/s1.

## References

[B1-jintelligence-08-00026] Agostino Alba, Johnson Janice, Pascual-Leone Juan (2010). Executive functions underlying multiplicative reasoning: Problem type matters. Journal of Experimental Child Psychology.

[B2-jintelligence-08-00026] American Psychiatric Association (2013). Diagnostic and Statistical Manual of Mental Disorders.

[B3-jintelligence-08-00026] Ayllón María F., Gómez Isabel A., Ballesta-Claver Julio (2016). Mathematical thinking and creativity through mathematical problem posing and solving. Propósitos y Representaciones.

[B4-jintelligence-08-00026] Baer John (2012). Domain specificity and the limits of creativity theory. The Journal of Creative Behavior.

[B5-jintelligence-08-00026] Bahar Kadir A., Maker Carol J. (2011). Exploring the relationship between mathematical creativity and mathematical achievement. Asia-Pacific Journal of Gifted and Talented Education.

[B6-jintelligence-08-00026] Bailey Aileen M., McDaniel William F., Thomas Roger K. (2007). Approached to the study of higher cognitive functions related to creativity in nonhuman animals. Methods.

[B7-jintelligence-08-00026] Baran Gülen, Erdogan Serap, Çakmak Aygen (2011). A study on the relationship between six-year-old children’s creativity and mathematical ability. International Education Studies.

[B8-jintelligence-08-00026] Benedek Mathias, Franz Fabiola, Heene Moritz, Neubauer Aljoscha C. (2012a). Differential effects of cognitive inhibition and intelligence on creativity. Personality and Individual Differences.

[B9-jintelligence-08-00026] Benedek Mathias, Könen Tanja, Neubauer Aljoscha C. (2012b). Associative abilities underlying creativity. Psychology of Aesthetics, Creativity, and the Arts.

[B10-jintelligence-08-00026] Benedek Mathias, Jauk Emanuel, Sommer Markus, Arendasy Martin, Neubauer Aljoscha C. (2014). Intelligence, creativity, and cognitive control: The common and differential involvement of executive functions in intelligence and creativity. Intelligence.

[B11-jintelligence-08-00026] Brydges Christopher R., Reid Corinne L., Fox Allison M., Anderson Mike (2012). A unitary executive function predicts intelligence in children. Intelligence.

[B12-jintelligence-08-00026] Bull Rebecca, Scerif Gaia (2001). Executive functioning as a predictor of children’s mathematics ability: Inhibition, switching, and working memory. Developmental Neuropsychology.

[B13-jintelligence-08-00026] Burch Giles St. J., Hemsley David R., Pavelis Christos, Corr Philip J. (2006). Personality, creativity and latent inhibition. European Journal of Personality.

[B14-jintelligence-08-00026] Carlson Stephanie M., Mandell Dorothy J., Williams Luke (2004). Executive function and theory of mind: Stability and prediction from ages 2 to 3. Developmental Psychology.

[B15-jintelligence-08-00026] Carson Shelley H., Peterson Jordan B., Higgins Daniel M. (2003). Decreased latent inhibition is associated with increased creative achievement in high-functioning individuals. Journal of Personality and Social Psychology.

[B16-jintelligence-08-00026] Cassotti Mathieu, Agogué Marine, Camarda Anaëlle, Houdé Olivier, Borst Grégoire (2016). Inhibitory control as a core process of creative problem solving and idea generation from childhood to adulthood. New Directions of Child and Adolescent Development.

[B17-jintelligence-08-00026] Centraal Bureau voor de Statistiek (CBS) (2015). Gezondheidsmetingen kinderen: 2001–2013 [Health measures of children: 2001–2013].

[B18-jintelligence-08-00026] Chen Chuansheng, Himsel Amy, Kasof Josph, Greenberger Ellen, Dmitrieva Julia (2006). Boundless creativity: Evidence for the domain generality of individual differences in creativity. The Journal of Creative Behavior.

[B19-jintelligence-08-00026] Chiappe Penny, Hasher Lynn, Siegel Linda S. (2000). Working memory, inhibitory control, and reading disability. Memory & Cognition.

[B20-jintelligence-08-00026] Chooi Weng-Tink, Long Holly E., Thompson Lee A. (2014). The Sternberg Triarchic Abilities Test (Level-H) is a measure of g. Journal of Intelligence.

[B21-jintelligence-08-00026] Clark Caron A. C., Pritchard Verena E., Woodward Lianne J. (2010). Preschool executive functioning abilities predict early mathematics achievement. Developmental Psychology.

[B22-jintelligence-08-00026] Clark Caron A. C., Nelson Jennifer M., Garza John, Sheffield Tiffany D., Wiebe Sandra A., Espy Kimberly A. (2014). Gaining control: Changing relations between executive control and processing speed and their relevance for mathematics achievement over course of the preschool period. Frontiers in Psychology.

[B23-jintelligence-08-00026] Cragg Lucy, Gilmore Camilla (2014). Skills underlying mathematics: The role of executive function in the development of mathematics proficiency. Trends in Neuroscience and Education.

[B24-jintelligence-08-00026] Cropley Arthur J. (2010). Defining and measuring creativity: Are creativity tests worth using?. Roeper Review.

[B25-jintelligence-08-00026] Csikszentmihalyi Mihály (1997). Flow and the Psychology of Discovery and Invention.

[B26-jintelligence-08-00026] Dajani Dina R., Uddin Lucina Q. (2015). Demystifying cognitive flexibility: Implications for clinical and developmental neuroscience. Trends in Neurosciences.

[B27-jintelligence-08-00026] Davidson Matthew C., Amso Dima, Anderson Loren C., Diamond Adele (2006). Development of cognitive control and executive functions from 4 to 13 years: Evidence from manipulations of memory, inhibition, and task switching. Neuropsychologia.

[B28-jintelligence-08-00026] De Beni Rossana, Palladino Paola, Pazzaglia Francesca, Cornoldi Cesare (1998). Increases in intrusion errors and working memory deficit of poor comprehenders. The Quarterly Journal of Experimental Psychology.

[B29-jintelligence-08-00026] De Ribaupierre Anik, Lecerf Thierry (2017). Intelligence and cognitive development: Three sides of the same coin. Journal of Intelligence.

[B30-jintelligence-08-00026] Diamond Adele (2013). Executive functions. Annual Review of Psychology.

[B31-jintelligence-08-00026] Duan Xiaoju, Wei Siwang, Wang Guiqing, Shi Jiannong (2010). The relationship between executive functions and intelligence on 11- to 12-year-old children. Psychological Test and Assessment Modeling.

[B32-jintelligence-08-00026] Edl Susanne, Benedek Mathias, Papousek Ilona, Weiss Elisabeth M., Fink Andreas (2014). Creativity and the Stroop interference effect. Personality and individual Differences.

[B33-jintelligence-08-00026] Espy Kimberly A., McDiarmid Melanie M., Cwik Mary F., Stalets Melissa M., Hamby Arlena, Senn Theresa E. (2004). The contribution of executive functions to emergent mathematic skills in preschool children. Developmental Neuropsychology.

[B34-jintelligence-08-00026] Friedman Naomi P., Miyake Akira (2004). The relations among inhibition and interference control functions: A latent-variable analysis. Journal of Experimental Psychology: General.

[B35-jintelligence-08-00026] Friedman Naomi P., Miyake Akira, Corley Robin P., Young Susan E., DeFries John C., Hewitt John K. (2006). Not all executive functions are related to intelligence. Psychological Science.

[B36-jintelligence-08-00026] Friso-Van den Bos Ilona, Van der Ven Sanne H. G., Kroesbergen Evelyn H., Van Luit Johannes E. H. (2013). Working memory and mathematics in primary school children: A meta-analysis. Educational Research Review.

[B37-jintelligence-08-00026] Gathercole Susan E., Pickering Susan J., Ambridge Benjamin, Wearing Hannah (2004). The structure of working memory from 4 to 15 years of age. Developmental Psychology.

[B38-jintelligence-08-00026] Groborz Magdalena, Necka Edward (2003). Creativity and cognitive control: Explorations of generation and evaluation skills. Creativity Research Journal.

[B39-jintelligence-08-00026] Hershikovitz Sara, Peled Irit, Littler Graham, Leikin Rosa, Berman Abraham, Koichu Boris (2009). Mathematical creativity in giftedness in elementary school: Task and teacher promoting creativity for all. Creativity in Mathematics and the Education of Gifted Students.

[B40-jintelligence-08-00026] Hong Eunsook, Aqui Yvette (2004). Cognitive and motivational characteristics of adolescents gifted in mathematics: Comparisons among students with different types of giftedness. Gifted Child Quarterly.

[B41-jintelligence-08-00026] Huizinga Mariëtte, Van der Molen Maurits W. (2007). Age-group differences in set-switching and set-maintenance on the Wisconsin Card Sorting Task. Developmental Neuropsychology.

[B42-jintelligence-08-00026] Huizinga Mariëtte, Dolan Conor V., Van der Molen Maurits W. (2006). Age-related change in executive function: Developmental trends and a latent variable analysis. Neuropsychologica.

[B43-jintelligence-08-00026] Janssen Judith, Scheltens Floor, Kraemer Jean-Marie (2007). Rekenen-Wiskunde. Handleiding [Manual Mathematics Test].

[B44-jintelligence-08-00026] Janssen Judith, Verhelst Norman, Engelen Ronald, Scheltens Floor (2010). Wetenschappelijke Verantwoording van de Toetsen LOVS Rekenen-Wiskunde Voor Groep 3 tot en Met 8 [Scientific Justification of the LOVS Mathematics Tests for Grade 1 to 6].

[B45-jintelligence-08-00026] Jeon Kyung-Nam, Moon Sidney M., French Brian (2011). Differential effects of divergent thinking, domain knowledge, and interest on creative performance in art and math. Creative Research Journal.

[B46-jintelligence-08-00026] Kālis Emīls, Roķe Līga, Krūmiņa Indra (2014). Investigation of psychometric properties of the Test for Creative Thinking—Drawing production: Evidence from study in Latvia. The Journal of Creative Behavior.

[B47-jintelligence-08-00026] Karbach Julia, Unger Kerstin (2014). Executive control training from middle school to adolescence. Frontiers in Psychology.

[B48-jintelligence-08-00026] Kattou Maria, Kontoyianni Katerina, Pitta-Pantazi Demetra, Christou Constantinos (2013). Connecting mathematical creativity to mathematical ability. ZDM Mathematics Education.

[B49-jintelligence-08-00026] Kaufman James C., Baer John (2004). Sure, I’m creative—But not in mathematics!: Self-reported creativity in diverse domains. Empirical Studies of the Arts.

[B50-jintelligence-08-00026] Kline Rex B. (2010). Principles and Practice of Structural Equation Modeling.

[B51-jintelligence-08-00026] Kroesbergen Evelyn H., Schoevers Eveline M. (2017). Creativity as predictor mathematical abilities in fourth graders in addition to number sense and working memory. Journal of Numerical Cognition.

[B52-jintelligence-08-00026] Kroesbergen Evelyn H., Dijk Marloes Van (2015). Working-memory and number sense as predictors of mathematical (dis-)ability. Zeitschrift für Psychologie.

[B53-jintelligence-08-00026] Lee Kerry, Ng Swee F., Pe Madeline L., Ang Su Y., Hasshim Muhammad N. A. M., Bull Rebecca (2012). The cognitive underpinnings of emerging mathematical skills: Executive functioning, patterns, numeracy, and arithmetic. British Journal of Educational Psychology.

[B54-jintelligence-08-00026] Lehto Juhani E., Juujärvi Petri, Kooistra Libbe, Pulkkinen Lea (2003). Dimensions of executive functioning: Evidence from children. Britisch Journal of Developmental Psychology.

[B55-jintelligence-08-00026] Leikin Roza, Leikin Roza, Berman Abraham, Koichu Boris (2009). Exploring mathematical creativity using multiple solution tasks. Creativity in Mathematics and the Education of Gifted Students.

[B56-jintelligence-08-00026] Leikin Mark (2014). Bilingualism and creative abilities in early childhood. English Linguistics Research.

[B57-jintelligence-08-00026] Leikin Roza, Lev Miriam (2013). Mathematical creativity in generally gifted and mathematically excelling adolescents: What makes the difference?. ZDM Mathematics Education.

[B58-jintelligence-08-00026] Leikin Roza, Pitta-Pantazi Demetra (2013). Creativity and mathematics education: The state of the art. ZDM Mathematics Education.

[B59-jintelligence-08-00026] Lin Chia-Yi, Cho Seokhee (2011). Predicting creative problem-solving in math from a dynamic system model of creative problem solving ability. Creativity Research Journal.

[B60-jintelligence-08-00026] MacKinnon David P., Lockwood Chondra M., Hoffman Jeanne M., West Stephen G., Sheets Virgil (2002). A comparison of methods to test mediation and other intervening variable effects. Psychological Methods.

[B61-jintelligence-08-00026] Mann Eric L. (2005). Mathematical Creativity and School Mathematics: Indicators of Mathematical Creativity in Middle School Students. Ph.D. dissertation.

[B62-jintelligence-08-00026] Mann Eric L. (2006). Creativity: The essence of mathematics. Journal for the Education of the Gifted.

[B63-jintelligence-08-00026] Mayes Susan D., Calhoun Susan L., Bixler Edward O., Zimmerman Dennis N. (2009). IQ and neuropsychological predictors of academic achievement. Learning and Individual Differences.

[B64-jintelligence-08-00026] Mednick Sarnoff A. (1962). The associative basis of the creative process. Psychological Review.

[B65-jintelligence-08-00026] Miyake Akira, Friedman Naomi P., Emerson Michael J., Witzki Alexander H., Howerter Amy (2000). The unity and diversity of executive functions and their contributions to complex “frontal lobe” tasks: A latent variable analysis. Cognitive Psychology.

[B66-jintelligence-08-00026] Moenikia Mahdi, Zahed-Babelan Adel (2010). A study of simple and multiple relations between mathematics attitude, academic motivation and intelligence quotient with mathematics achievement. Procedia-Social and Behavioral Sciences.

[B67-jintelligence-08-00026] Mumford Michael D., Gustafson Sigrid B. (1988). Creativity syndrome: Integration, application, and innovation. Psychological Bulletin.

[B68-jintelligence-08-00026] Nijstad Bernard A., De Dreu Carsten K. W., Rietzschel Eric F., Baas Matthijs (2010). The dual pathway to creativity model: Creative ideation as a function of flexibility and persistence. European Review of Social Psychology.

[B69-jintelligence-08-00026] Nusbaum Emily C., Silvia Paul J. (2011). Are intelligence and creativity really so different?: Fluid intelligence, executive processes, and strategy use in divergent thinking. Intelligence.

[B70-jintelligence-08-00026] Pan Xuan, Yu Huihong (2018). Different effects of cognitive shifting and intelligence on creativity. Journal of Creative Behavior.

[B71-jintelligence-08-00026] Passolunghi Maria C., Siegel Linda S. (2001). Short-term memory, working memory, and inhibitory control in children with difficulties in arithmetic problem solving. Journal of Experimental Child Psychology.

[B72-jintelligence-08-00026] Plucker Jonathan A. (1999). Reanalyses of student responses to creativity checklists: Evidence of content generality. Journal of Creative Behavior.

[B73-jintelligence-08-00026] Plucker Jonathan A., Beghetto Ronald A., Dow Gayle T. (2004). Why isn’t creativity more important to educational psychologists? Potentials, pitfalls, and future directions of creativity research. Educational Psychologist.

[B74-jintelligence-08-00026] Purić Danka, Pavlović Maša (2012). Executive function of shifting: Factorial structure and relations to personality and intelligence domains. Suvremena Psihologija.

[B75-jintelligence-08-00026] Raghubar Kimberly P., Barner Marcia A., Hecht Steven A. (2010). Working memory and mathematics: A review of developmental, individual difference, and cognitive approaches. Learning and Individual Differences.

[B76-jintelligence-08-00026] Razumnikova Olga M. (2007). Creativity related cortex activity in the remote associates task. Brain Research Bulletin.

[B77-jintelligence-08-00026] Rhoades Brittany L., Greenberg Mark T., Lanza Stephanie T., Blair Clancy (2011). Demographic and familial predictors of early executive function development: Contribution of a person-centered perspective. Journal of Experimental Child Psychology.

[B78-jintelligence-08-00026] Sak Ugur, Maker Carol J. (2006). Developmental variation in children’s creative mathematical thinking as a function of schooling, age, and knowledge. Creativity Research Journal.

[B79-jintelligence-08-00026] Sawyer Keith R. (2006). Defining creativity through assessment. The Science of Human Innovation.

[B80-jintelligence-08-00026] Schoevers Eveline M., Kroesbergen Evelyn H., Kattou Maria (2018). Mathematical creativity: A combination of domain-general and domain-specific mathematical skills. Journal of Creative Behavior.

[B81-jintelligence-08-00026] Schreiber James B., Nora Amaury, Stage Frances K., Barlow Elizabeth A., King Jamie (2006). Reporting structural equation modeling and confirmatory factor analysis results: A review. The Journal of Educational Research.

[B82-jintelligence-08-00026] Scibinetti Patrizia, Tocci Nicoletta, Pesce Caterina (2011). Motor creativity and creative thinking in children: The diverging role of inhibition. Creativity Research Journal.

[B83-jintelligence-08-00026] Sharma Shivani, Babu Nandita (2017). Interplay between creativity, executive function and working memory in middle-aged and older adults. Creativity Research Journal.

[B84-jintelligence-08-00026] Shrout Patrick E., Bolger Niall (2002). Mediation in experimental and nonexperimental studies: New procedures and recommendations. Psychological Methods.

[B85-jintelligence-08-00026] Singer Florence M., Sheffield Linda J., Leikin Roza (2017). Advancements in research on creativity and giftedness in mathematics education: Introduction to the special issue. ZDM Mathematics Education.

[B86-jintelligence-08-00026] Sriraman Bharath (2004). The characteristics of mathematical creativity. The Mathematics Education.

[B87-jintelligence-08-00026] Sriraman Bharath (2005). Are giftedness and creativity synonyms in mathematics?. The Journal of Secondary Gifted Education.

[B88-jintelligence-08-00026] Stein Morris I. (1953). Creativity and culture. Journal of Psychology.

[B89-jintelligence-08-00026] Stolte Marije, Kroesbergen Evelyn H., Van Luit Johannes E. H. (2018). Inhibition friend or foe: Cognitive inhibition as a moderator between mathematical ability and mathematical creativity in primary school students. Personality and Individual Differences.

[B90-jintelligence-08-00026] Tella Adedeji (2007). The impact of motivation on student’s academic achievement and learning outcomes in mathematics among secondary school students in Nigeria. Eurasia Journal of Mathematics, Science & Technology Education.

[B91-jintelligence-08-00026] Toll Sylke W. M., Van der Ven Sanne H. G., Kroesbergen Evelyn H., Van Luit Johannes E. H. (2011). Executive functions as predictors of math learning disabilities. Journal of Learning Disabilities.

[B92-jintelligence-08-00026] Torrance Ellis P. (1974). Norms Technical Manual: Torrance Tests of Creative Thinking.

[B93-jintelligence-08-00026] Urban Klaus K. (2004). The Test for Creative Thinking—Drawing Production (TCT-DP) the concept, application, evaluation, and international studies. Psychology Science.

[B94-jintelligence-08-00026] Van de Weijer-Bergsma Eva, Kroesbergen Evelyn H., Van Luit Johannes E. H. (2015a). Verbal and visual-spatial working memory and mathematical ability in different domains throughout primary school. Memory & Cognition.

[B95-jintelligence-08-00026] Van de Weijer-Bergsma Eva, Kroesbergen Evelyn H., Prast Emilie J., Van Luit Johannes E. H. (2015b). Validity and reliability of an online visual–spatial working memory task for self-reliant administration in school-aged children. Behavior Research Methods.

[B96-jintelligence-08-00026] Van de Weijer-Bergsma Eva, Kroesbergen Evelyn H., Jolani Shahab, Van Luit Johannes E. H. (2016). The Monkey game: A computerized verbal working memory task for self-reliant administration in primary school children. Behavior Research Methods.

[B97-jintelligence-08-00026] Van der Sluis Sophie, De Jong Peter F., Van der Leij Aryan (2004). Inhibition and shifting in children with learning deficits in arithmetic and reading. Journal of Experimental Child Psychology.

[B98-jintelligence-08-00026] Van der Sluis Sophie, De Jong Peter F., Van der Leij Aryan (2007). Executive functioning in children, and its relations with reasoning, reading, and arithmetic. Intelligence.

[B99-jintelligence-08-00026] Van der Ven Sanne H. G., Kroesbergen Evelyn H., Boom Jan, Leseman Paul P. M. (2012). The development of executive functions and early mathematics: A dynamic relationship. British Journal of Educational Psychology.

[B100-jintelligence-08-00026] Vartanian Oshin, Martindale Colin, Kwiatkowski Jonna (2007). Creative potential, attention, and speed of information processing. Personality and Individual Differences.

[B101-jintelligence-08-00026] White Holly A., Shah Priti (2006). Uninhibited imaginations: Creativity in adults with attention-deficit/hyperactivity disorder. Personality and Individual Differences.

[B102-jintelligence-08-00026] Yeniad Nihal, Malda Maike, Mesman Judi, Van IJzendoorn Marinus H., Pieper Suzanne (2013). Shifting ability predicts mat hand reading performance in children: A meta-analytical study. Learning and Individual Differences.

[B103-jintelligence-08-00026] Zabelina Darya L., Colzato Lorenza, Beeman Mark, Hommel Bernhard (2016). Dopamine and the creative mind: Individual differences in creativity are predicted by interactions between dopamine genes DAT and COMT. PLoS ONE.

[B104-jintelligence-08-00026] Zhao Xinshu, Lynch John G., Chen Qimei (2010). Reconsidering Baron and Kenny: Myths and truths about mediation analysis. Journal of Consumer Research.

