# Supplementary material for: The Contribution of Executive Functions in Predicting Mathematical Creativity in Typical Elementary School Classes: A Twofold Role for Updating
Source: J Intell. 2020 Jun 2;8(2):26. doi: 10.3390/jintelligence8020026 (PMC7713010; doi:10.3390/jintelligence8020026)
Supplement: Supplementary file 1 [file jintelligence-08-00026-s001.zip › direct model without missings 01052020.AmosOutput]

direct model without missings or intelligence 01052020.amw


#### \\Client\D$\Corona Backup 15032020\Selfpublished Articles\Creamath Paper\Journal of Intelligence\Revision\direct model without missings or intelligence 01052020.amw

##### Analysis Summary

##### Date and Time

Date: vrijdag 1 mei 2020

Time: 11:01:23

##### Title

direct model without missings or intelligence 01052020: vrijdag 1 mei 2020 11:01

##### Groups

##### Group number 1 (Group number 1)

##### Notes for Group (Group number 1)

The model is recursive.

Sample size = 278

##### Variable Summary (Group number 1)

##### Your model contains the following variables (Group number 1)

Observed, endogenous variables

URC\_Flu\_1

URC\_Flex\_1

URC\_Org\_1

URC\_Flu\_3

URC\_Flex\_3

URC\_Org\_3

URC\_Flu\_4

URC\_Flex\_4

URC\_Org\_4

MeanRTShift

UpdatingCombined

InhibitionIncongruentRT

MathKnowledge\_Cito

Creativity\_TCTDP

Unobserved, endogenous variables

Task\_1

Math\_DT

Task\_3

Task\_4

Unobserved, exogenous variables

e1

e2

e3

e5

e4

e6

e8

e7

e9

e10

e11

e13

e12

e14

e15

e16

e17

e18

##### Variable counts (Group number 1)

|  |  |
| --- | --- |
| Number of variables in your model: | 36 |
| Number of observed variables: | 14 |
| Number of unobserved variables: | 22 |
| Number of exogenous variables: | 18 |
| Number of endogenous variables: | 18 |

##### Parameter Summary (Group number 1)

|  | Weights | Covariances | Variances | Means | Intercepts | Total |
| --- | --- | --- | --- | --- | --- | --- |
| Fixed | 22 | 0 | 0 | 18 | 4 | 44 |
| Labeled | 0 | 0 | 0 | 0 | 0 | 0 |
| Unlabeled | 13 | 12 | 18 | 0 | 14 | 57 |
| Total | 35 | 12 | 18 | 18 | 18 | 101 |

##### Assessment of normality (Group number 1)

| Variable | min | max | skew | c.r. | kurtosis | c.r. |
| --- | --- | --- | --- | --- | --- | --- |
| Creativity\_TCTDP | 4,000 | 47,000 | ,629 | 4,282 | -,451 | -1,535 |
| MathKnowledge\_Cito | -6,287 | 3,154 | -1,196 | -8,144 | 4,673 | 15,906 |
| InhibitionIncongruentRT | 493,150 | 1315,200 | 1,111 | 7,560 | 1,603 | 5,457 |
| UpdatingCombined | -3,198 | 1,633 | -,749 | -5,095 | ,698 | 2,375 |
| MeanRTShift | 492,640 | 1973,950 | -,472 | -3,215 | ,735 | 2,500 |
| URC\_Org\_4 | ,000 | 1,000 | ,178 | 1,210 | -,819 | -2,787 |
| URC\_Flex\_4 | ,000 | 5,000 | ,108 | ,738 | -,399 | -1,359 |
| URC\_Flu\_4 | ,000 | 9,000 | 1,076 | 7,323 | 2,096 | 7,134 |
| URC\_Org\_3 | ,000 | 1,000 | ,642 | 4,373 | ,309 | 1,051 |
| URC\_Flex\_3 | ,000 | 4,000 | -,549 | -3,734 | ,307 | 1,044 |
| URC\_Flu\_3 | ,000 | 48,000 | 2,484 | 16,911 | 7,228 | 24,602 |
| URC\_Org\_1 | ,000 | 1,000 | ,106 | ,722 | -1,465 | -4,986 |
| URC\_Flex\_1 | ,000 | 3,000 | -,144 | -,980 | -,324 | -1,101 |
| URC\_Flu\_1 | ,000 | 8,000 | ,859 | 5,849 | 1,002 | 3,412 |
| Multivariate |  |  |  |  | 28,506 | 11,228 |

##### Observations farthest from the centroid (Mahalanobis distance) (Group number 1)

| Observation number | Mahalanobis d-squared | p1 | p2 |
| --- | --- | --- | --- |
| 165 | 57,105 | ,000 | ,000 |
| 35 | 49,805 | ,000 | ,000 |
| 119 | 42,031 | ,000 | ,000 |
| 218 | 40,284 | ,000 | ,000 |
| 251 | 37,985 | ,001 | ,000 |
| 198 | 33,125 | ,003 | ,000 |
| 255 | 32,496 | ,003 | ,000 |
| 123 | 32,222 | ,004 | ,000 |
| 168 | 30,729 | ,006 | ,000 |
| 24 | 30,201 | ,007 | ,000 |
| 97 | 28,954 | ,011 | ,000 |
| 48 | 28,898 | ,011 | ,000 |
| 104 | 28,868 | ,011 | ,000 |
| 224 | 28,679 | ,012 | ,000 |
| 59 | 28,570 | ,012 | ,000 |
| 245 | 27,853 | ,015 | ,000 |
| 137 | 27,621 | ,016 | ,000 |
| 90 | 27,608 | ,016 | ,000 |
| 253 | 26,701 | ,021 | ,000 |
| 103 | 25,965 | ,026 | ,000 |
| 151 | 25,284 | ,032 | ,000 |
| 254 | 25,245 | ,032 | ,000 |
| 98 | 24,310 | ,042 | ,002 |
| 102 | 24,241 | ,043 | ,001 |
| 157 | 24,156 | ,044 | ,001 |
| 54 | 23,989 | ,046 | ,001 |
| 22 | 23,183 | ,057 | ,006 |
| 86 | 23,117 | ,058 | ,004 |
| 163 | 22,565 | ,068 | ,014 |
| 14 | 22,169 | ,075 | ,030 |
| 188 | 22,121 | ,076 | ,022 |
| 150 | 21,845 | ,082 | ,033 |
| 28 | 21,690 | ,085 | ,034 |
| 159 | 21,684 | ,085 | ,022 |
| 122 | 21,440 | ,091 | ,031 |
| 152 | 21,428 | ,091 | ,021 |
| 261 | 21,420 | ,091 | ,014 |
| 31 | 21,235 | ,096 | ,017 |
| 67 | 20,949 | ,103 | ,030 |
| 190 | 20,582 | ,113 | ,065 |
| 57 | 20,547 | ,114 | ,051 |
| 175 | 20,298 | ,121 | ,078 |
| 121 | 20,200 | ,124 | ,075 |
| 259 | 19,821 | ,136 | ,158 |
| 234 | 19,791 | ,137 | ,131 |
| 5 | 19,600 | ,143 | ,165 |
| 195 | 19,550 | ,145 | ,146 |
| 239 | 19,112 | ,161 | ,317 |
| 161 | 19,091 | ,161 | ,274 |
| 91 | 18,772 | ,174 | ,420 |
| 138 | 18,699 | ,177 | ,409 |
| 176 | 18,593 | ,181 | ,422 |
| 213 | 18,592 | ,181 | ,364 |
| 162 | 18,311 | ,193 | ,503 |
| 72 | 18,297 | ,194 | ,453 |
| 211 | 18,218 | ,197 | ,451 |
| 249 | 18,081 | ,203 | ,492 |
| 108 | 17,731 | ,219 | ,688 |
| 228 | 17,492 | ,231 | ,790 |
| 182 | 17,430 | ,234 | ,783 |
| 149 | 17,246 | ,243 | ,841 |
| 133 | 17,242 | ,244 | ,806 |
| 3 | 17,138 | ,249 | ,823 |
| 180 | 16,991 | ,257 | ,860 |
| 203 | 16,984 | ,257 | ,830 |
| 260 | 16,857 | ,264 | ,858 |
| 247 | 16,746 | ,270 | ,877 |
| 270 | 16,638 | ,276 | ,893 |
| 52 | 16,637 | ,276 | ,866 |
| 240 | 16,635 | ,276 | ,835 |
| 41 | 16,526 | ,282 | ,856 |
| 174 | 16,239 | ,299 | ,938 |
| 142 | 16,233 | ,299 | ,921 |
| 126 | 16,197 | ,301 | ,912 |
| 276 | 16,182 | ,302 | ,895 |
| 264 | 16,177 | ,303 | ,871 |
| 256 | 15,876 | ,321 | ,951 |
| 147 | 15,824 | ,324 | ,949 |
| 73 | 15,768 | ,328 | ,948 |
| 248 | 15,681 | ,333 | ,954 |
| 272 | 15,619 | ,337 | ,955 |
| 51 | 15,560 | ,341 | ,955 |
| 127 | 15,556 | ,341 | ,942 |
| 236 | 15,527 | ,343 | ,935 |
| 112 | 15,493 | ,345 | ,928 |
| 85 | 15,422 | ,350 | ,932 |
| 21 | 15,279 | ,359 | ,954 |
| 125 | 15,155 | ,368 | ,967 |
| 47 | 15,133 | ,369 | ,961 |
| 235 | 15,096 | ,372 | ,958 |
| 179 | 15,044 | ,375 | ,957 |
| 145 | 14,998 | ,378 | ,956 |
| 99 | 14,901 | ,385 | ,964 |
| 153 | 14,873 | ,387 | ,959 |
| 93 | 14,815 | ,391 | ,960 |
| 30 | 14,737 | ,396 | ,965 |
| 113 | 14,721 | ,398 | ,958 |
| 227 | 14,687 | ,400 | ,954 |
| 242 | 14,638 | ,403 | ,953 |
| 34 | 14,594 | ,406 | ,951 |

##### Sample Moments (Group number 1)

##### Sample Covariances (Group number 1)

|  | Creativity\_TCTDP | MathKnowledge\_Cito | InhibitionIncongruentRT | UpdatingCombined | MeanRTShift | URC\_Org\_4 | URC\_Flex\_4 | URC\_Flu\_4 | URC\_Org\_3 | URC\_Flex\_3 | URC\_Flu\_3 | URC\_Org\_1 | URC\_Flex\_1 | URC\_Flu\_1 |
| --- | --- | --- | --- | --- | --- | --- | --- | --- | --- | --- | --- | --- | --- | --- |
| Creativity\_TCTDP | 91,437 |
| MathKnowledge\_Cito | ,160 | ,998 |
| InhibitionIncongruentRT | -94,518 | -9,985 | 22972,423 |
| UpdatingCombined | 1,802 | ,158 | -47,457 | ,694 |
| MeanRTShift | -208,923 | -20,132 | 11910,364 | -16,644 | 55729,438 |
| URC\_Org\_4 | ,406 | ,028 | -2,853 | ,025 | -3,968 | ,085 |
| URC\_Flex\_4 | 1,088 | ,150 | -22,641 | ,201 | -13,859 | ,168 | ,993 |
| URC\_Flu\_4 | 2,223 | ,164 | -38,698 | ,331 | -27,877 | ,327 | 1,252 | 2,478 |
| URC\_Org\_3 | ,243 | ,013 | -7,996 | ,058 | -4,379 | ,010 | ,065 | ,099 | ,053 |
| URC\_Flex\_3 | ,865 | ,133 | -19,051 | ,206 | -10,955 | ,038 | ,240 | ,382 | ,117 | ,730 |
| URC\_Flu\_3 | 7,635 | ,436 | -335,670 | 2,427 | -187,023 | ,354 | 1,718 | 2,827 | ,627 | 2,412 | 74,667 |
| URC\_Org\_1 | ,156 | ,023 | -4,669 | ,057 | -4,565 | ,021 | ,061 | ,137 | ,020 | ,050 | ,614 | ,115 |
| URC\_Flex\_1 | 1,335 | ,121 | -26,947 | ,224 | -14,716 | ,051 | ,218 | ,346 | ,037 | ,101 | 1,616 | ,134 | ,575 |
| URC\_Flu\_1 | 2,130 | ,181 | -35,352 | ,426 | -41,604 | ,120 | ,442 | ,787 | ,090 | ,295 | 3,001 | ,324 | ,788 | 2,067 |

Condition number = 1988132,409

Eigenvalues

59604,478 19103,527 92,505 68,237 3,568 1,761 ,999 ,608 ,442 ,283 ,215 ,061 ,040 ,030

Determinant of sample covariance matrix = 53559569,850

##### Sample Correlations (Group number 1)

|  | Creativity\_TCTDP | MathKnowledge\_Cito | InhibitionIncongruentRT | UpdatingCombined | MeanRTShift | URC\_Org\_4 | URC\_Flex\_4 | URC\_Flu\_4 | URC\_Org\_3 | URC\_Flex\_3 | URC\_Flu\_3 | URC\_Org\_1 | URC\_Flex\_1 | URC\_Flu\_1 |
| --- | --- | --- | --- | --- | --- | --- | --- | --- | --- | --- | --- | --- | --- | --- |
| Creativity\_TCTDP | 1,000 |
| MathKnowledge\_Cito | ,017 | 1,000 |
| InhibitionIncongruentRT | -,065 | -,066 | 1,000 |
| UpdatingCombined | ,226 | ,189 | -,376 | 1,000 |
| MeanRTShift | -,093 | -,085 | ,333 | -,085 | 1,000 |
| URC\_Org\_4 | ,145 | ,096 | -,065 | ,104 | -,058 | 1,000 |
| URC\_Flex\_4 | ,114 | ,150 | -,150 | ,242 | -,059 | ,577 | 1,000 |
| URC\_Flu\_4 | ,148 | ,104 | -,162 | ,253 | -,075 | ,712 | ,798 | 1,000 |
| URC\_Org\_3 | ,110 | ,057 | -,229 | ,303 | -,080 | ,147 | ,282 | ,274 | 1,000 |
| URC\_Flex\_3 | ,106 | ,156 | -,147 | ,290 | -,054 | ,151 | ,282 | ,284 | ,592 | 1,000 |
| URC\_Flu\_3 | ,092 | ,051 | -,256 | ,337 | -,092 | ,140 | ,199 | ,208 | ,315 | ,327 | 1,000 |
| URC\_Org\_1 | ,048 | ,067 | -,091 | ,201 | -,057 | ,213 | ,181 | ,256 | ,257 | ,172 | ,210 | 1,000 |
| URC\_Flex\_1 | ,184 | ,160 | -,234 | ,355 | -,082 | ,231 | ,289 | ,290 | ,213 | ,155 | ,247 | ,521 | 1,000 |
| URC\_Flu\_1 | ,155 | ,126 | -,162 | ,356 | -,123 | ,287 | ,308 | ,348 | ,270 | ,240 | ,242 | ,665 | ,723 | 1,000 |

Condition number = 24,772

Eigenvalues

4,115 1,640 1,442 1,201 ,996 ,978 ,840 ,687 ,496 ,451 ,393 ,371 ,225 ,166

##### Sample Means (Group number 1)

|  | Creativity\_TCTDP | MathKnowledge\_Cito | InhibitionIncongruentRT | UpdatingCombined | MeanRTShift | URC\_Org\_4 | URC\_Flex\_4 | URC\_Flu\_4 | URC\_Org\_3 | URC\_Flex\_3 | URC\_Flu\_3 | URC\_Org\_1 | URC\_Flex\_1 | URC\_Flu\_1 |
| --- | --- | --- | --- | --- | --- | --- | --- | --- | --- | --- | --- | --- | --- | --- |
|  | 20,331 | ,006 | 790,082 | ,036 | 1386,833 | ,498 | 1,777 | 2,306 | ,417 | 2,255 | 8,331 | ,551 | 1,705 | 2,367 |

##### Models

##### Default model (Default model)

##### Notes for Model (Default model)

##### Computation of degrees of freedom (Default model)

|  |  |
| --- | --- |
| Number of distinct sample moments: | 119 |
| Number of distinct parameters to be estimated: | 57 |
| Degrees of freedom (119 - 57): | 62 |

##### Result (Default model)

Minimum was achieved

Chi-square = 97,316

Degrees of freedom = 62

Probability level = ,003

##### Group number 1 (Group number 1 - Default model)

##### Estimates (Group number 1 - Default model)

##### Scalar Estimates (Group number 1 - Default model)

##### Maximum Likelihood Estimates

##### Regression Weights: (Group number 1 - Default model)

|  |  |  | Estimate | S.E. | C.R. | P | Label |
| --- | --- | --- | --- | --- | --- | --- | --- |
| Math\_DT | <--- | MathKnowledge\_Cito | ,089 | ,061 | 1,461 | ,144 | par\_12 |
| Math\_DT | <--- | Creativity\_TCTDP | ,012 | ,006 | 1,875 | ,061 | par\_13 |
| Math\_DT | <--- | InhibitionIncongruentRT | -,001 | ,000 | -1,563 | ,118 | par\_14 |
| Math\_DT | <--- | MeanRTShift | ,000 | ,000 | -,862 | ,389 | par\_15 |
| Math\_DT | <--- | UpdatingCombined | ,497 | ,094 | 5,314 | \*\*\* | par\_16 |
| Task\_1 | <--- | Math\_DT | 1,000 |  |
| Task\_3 | <--- | Math\_DT | 3,076 | ,710 | 4,333 | \*\*\* | par\_1 |
| Task\_4 | <--- | Math\_DT | ,978 | ,161 | 6,069 | \*\*\* | par\_2 |
| URC\_Flu\_1 | <--- | Task\_1 | 1,000 |  |
| URC\_Flex\_1 | <--- | Task\_1 | ,419 | ,031 | 13,486 | \*\*\* | par\_3 |
| URC\_Org\_1 | <--- | Task\_1 | ,173 | ,014 | 12,770 | \*\*\* | par\_4 |
| URC\_Flu\_3 | <--- | Task\_3 | 1,000 |  |
| URC\_Flex\_3 | <--- | Task\_3 | ,162 | ,026 | 6,323 | \*\*\* | par\_5 |
| URC\_Org\_3 | <--- | Task\_3 | ,044 | ,007 | 6,251 | \*\*\* | par\_6 |
| URC\_Flu\_4 | <--- | Task\_4 | 1,000 |  |
| URC\_Flex\_4 | <--- | Task\_4 | ,518 | ,031 | 16,731 | \*\*\* | par\_7 |
| URC\_Org\_4 | <--- | Task\_4 | ,135 | ,009 | 14,411 | \*\*\* | par\_8 |

##### Standardized Regression Weights: (Group number 1 - Default model)

|  |  |  | Estimate |
| --- | --- | --- | --- |
| Math\_DT | <--- | MathKnowledge\_Cito | ,105 |
| Math\_DT | <--- | Creativity\_TCTDP | ,136 |
| Math\_DT | <--- | InhibitionIncongruentRT | -,125 |
| Math\_DT | <--- | MeanRTShift | -,065 |
| Math\_DT | <--- | UpdatingCombined | ,488 |
| Task\_1 | <--- | Math\_DT | ,627 |
| Task\_3 | <--- | Math\_DT | ,666 |
| Task\_4 | <--- | Math\_DT | ,538 |
| URC\_Flu\_1 | <--- | Task\_1 | ,948 |
| URC\_Flex\_1 | <--- | Task\_1 | ,757 |
| URC\_Org\_1 | <--- | Task\_1 | ,696 |
| URC\_Flu\_3 | <--- | Task\_3 | ,455 |
| URC\_Flex\_3 | <--- | Task\_3 | ,750 |
| URC\_Org\_3 | <--- | Task\_3 | ,751 |
| URC\_Flu\_4 | <--- | Task\_4 | ,986 |
| URC\_Flex\_4 | <--- | Task\_4 | ,807 |
| URC\_Org\_4 | <--- | Task\_4 | ,719 |

##### Intercepts: (Group number 1 - Default model)

|  |  |  | Estimate | S.E. | C.R. | P | Label |
| --- | --- | --- | --- | --- | --- | --- | --- |
| MeanRTShift |  |  | 1386,833 | 14,184 | 97,774 | \*\*\* | par\_26 |
| UpdatingCombined |  |  | ,036 | ,050 | ,728 | ,467 | par\_27 |
| InhibitionIncongruentRT |  |  | 790,082 | 9,107 | 86,758 | \*\*\* | par\_28 |
| Creativity\_TCTDP |  |  | 20,331 | ,575 | 35,386 | \*\*\* | par\_29 |
| MathKnowledge\_Cito |  |  | ,006 | ,060 | ,108 | ,914 | par\_30 |
| URC\_Flu\_1 |  |  | 2,979 | ,453 | 6,581 | \*\*\* | par\_31 |
| URC\_Flex\_1 |  |  | 1,961 | ,192 | 10,206 | \*\*\* | par\_32 |
| URC\_Org\_1 |  |  | ,657 | ,080 | 8,217 | \*\*\* | par\_33 |
| URC\_Flu\_3 |  |  | 10,212 | 1,506 | 6,780 | \*\*\* | par\_34 |
| URC\_Flex\_3 |  |  | 2,560 | ,230 | 11,148 | \*\*\* | par\_35 |
| URC\_Org\_3 |  |  | ,499 | ,062 | 7,998 | \*\*\* | par\_36 |
| URC\_Flu\_4 |  |  | 2,904 | ,446 | 6,514 | \*\*\* | par\_37 |
| URC\_Flex\_4 |  |  | 2,087 | ,234 | 8,916 | \*\*\* | par\_38 |
| URC\_Org\_4 |  |  | ,579 | ,062 | 9,388 | \*\*\* | par\_39 |

##### Covariances: (Group number 1 - Default model)

|  |  |  | Estimate | S.E. | C.R. | P | Label |
| --- | --- | --- | --- | --- | --- | --- | --- |
| e1 | <--> | e3 | 11910,364 | 2265,816 | 5,257 | \*\*\* | par\_9 |
| e2 | <--> | e3 | -47,457 | 8,105 | -5,855 | \*\*\* | par\_10 |
| e1 | <--> | e2 | -16,644 | 11,860 | -1,403 | ,161 | par\_11 |
| e6 | <--> | e9 | -,299 | ,417 | -,717 | ,474 | par\_17 |
| e6 | <--> | e12 | -,052 | ,048 | -1,087 | ,277 | par\_18 |
| e9 | <--> | e12 | -,099 | ,394 | -,252 | ,801 | par\_19 |
| e7 | <--> | e10 | -,036 | ,022 | -1,673 | ,094 | par\_20 |
| e7 | <--> | e13 | ,037 | ,021 | 1,811 | ,070 | par\_21 |
| e10 | <--> | e13 | ,015 | ,025 | ,611 | ,541 | par\_22 |
| e8 | <--> | e11 | ,005 | ,003 | 1,921 | ,055 | par\_23 |
| e8 | <--> | e14 | ,002 | ,003 | ,534 | ,593 | par\_24 |
| e11 | <--> | e14 | -,002 | ,002 | -,696 | ,486 | par\_25 |

##### Correlations: (Group number 1 - Default model)

|  |  |  | Estimate |
| --- | --- | --- | --- |
| e1 | <--> | e3 | ,333 |
| e2 | <--> | e3 | -,376 |
| e1 | <--> | e2 | -,085 |
| e6 | <--> | e9 | -,086 |
| e6 | <--> | e12 | -,444 |
| e9 | <--> | e12 | -,050 |
| e7 | <--> | e10 | -,132 |
| e7 | <--> | e13 | ,130 |
| e10 | <--> | e13 | ,046 |
| e8 | <--> | e11 | ,146 |
| e8 | <--> | e14 | ,035 |
| e11 | <--> | e14 | -,052 |

##### Variances: (Group number 1 - Default model)

|  |  |  | Estimate | S.E. | C.R. | P | Label |
| --- | --- | --- | --- | --- | --- | --- | --- |
| e1 |  |  | 55729,438 | 4735,434 | 11,769 | \*\*\* | par\_40 |
| e2 |  |  | ,694 | ,059 | 11,769 | \*\*\* | par\_41 |
| e3 |  |  | 22972,423 | 1952,009 | 11,769 | \*\*\* | par\_42 |
| e5 |  |  | ,998 | ,085 | 11,769 | \*\*\* | par\_43 |
| e4 |  |  | 91,437 | 7,770 | 11,769 | \*\*\* | par\_44 |
| e15 |  |  | ,474 | ,127 | 3,731 | \*\*\* | par\_45 |
| e16 |  |  | 1,113 | ,179 | 6,201 | \*\*\* | par\_46 |
| e17 |  |  | 8,549 | 2,642 | 3,236 | ,001 | par\_47 |
| e18 |  |  | 1,693 | ,214 | 7,916 | \*\*\* | par\_48 |
| e6 |  |  | ,205 | ,096 | 2,125 | ,034 | par\_49 |
| e8 |  |  | ,059 | ,006 | 10,415 | \*\*\* | par\_50 |
| e7 |  |  | ,240 | ,027 | 8,878 | \*\*\* | par\_51 |
| e9 |  |  | 58,792 | 5,507 | 10,676 | \*\*\* | par\_52 |
| e10 |  |  | ,315 | ,047 | 6,641 | \*\*\* | par\_53 |
| e11 |  |  | ,023 | ,003 | 6,621 | \*\*\* | par\_54 |
| e13 |  |  | ,342 | ,039 | 8,704 | \*\*\* | par\_55 |
| e12 |  |  | ,066 | ,096 | ,690 | ,490 | par\_56 |
| e14 |  |  | ,041 | ,004 | 10,694 | \*\*\* | par\_57 |

##### Squared Multiple Correlations: (Group number 1 - Default model)

|  |  |  | Estimate |
| --- | --- | --- | --- |
| Creativity\_TCTDP |  |  | ,000 |
| MathKnowledge\_Cito |  |  | ,000 |
| InhibitionIncongruentRT |  |  | ,000 |
| UpdatingCombined |  |  | ,000 |
| MeanRTShift |  |  | ,000 |
| Math\_DT |  |  | ,343 |
| Task\_4 |  |  | ,290 |
| Task\_3 |  |  | ,444 |
| Task\_1 |  |  | ,394 |
| URC\_Org\_4 |  |  | ,517 |
| URC\_Flex\_4 |  |  | ,652 |
| URC\_Flu\_4 |  |  | ,973 |
| URC\_Org\_3 |  |  | ,564 |
| URC\_Flex\_3 |  |  | ,562 |
| URC\_Flu\_3 |  |  | ,207 |
| URC\_Org\_1 |  |  | ,484 |
| URC\_Flex\_1 |  |  | ,573 |
| URC\_Flu\_1 |  |  | ,900 |

##### Matrices (Group number 1 - Default model)

##### Implied Covariances (Group number 1 - Default model)

|  | Creativity\_TCTDP | MathKnowledge\_Cito | InhibitionIncongruentRT | UpdatingCombined | MeanRTShift | URC\_Org\_4 | URC\_Flex\_4 | URC\_Flu\_4 | URC\_Org\_3 | URC\_Flex\_3 | URC\_Flu\_3 | URC\_Org\_1 | URC\_Flex\_1 | URC\_Flu\_1 |
| --- | --- | --- | --- | --- | --- | --- | --- | --- | --- | --- | --- | --- | --- | --- |
| Creativity\_TCTDP | 91,437 |
| MathKnowledge\_Cito | ,000 | ,998 |
| InhibitionIncongruentRT | ,000 | ,000 | 22972,423 |
| UpdatingCombined | ,000 | ,000 | -47,457 | ,694 |
| MeanRTShift | ,000 | ,000 | 11910,364 | -16,644 | 55729,438 |
| URC\_Org\_4 | ,146 | ,012 | -5,614 | ,051 | -3,915 | ,084 |
| URC\_Flex\_4 | ,560 | ,045 | -21,518 | ,194 | -15,004 | ,167 | ,983 |
| URC\_Flu\_4 | 1,081 | ,087 | -41,519 | ,374 | -28,951 | ,322 | 1,236 | 2,450 |
| URC\_Org\_3 | ,149 | ,012 | -5,719 | ,052 | -3,988 | ,011 | ,049 | ,095 | ,052 |
| URC\_Flex\_3 | ,551 | ,044 | -21,168 | ,191 | -14,761 | ,048 | ,198 | ,352 | ,109 | ,719 |
| URC\_Flu\_3 | 3,399 | ,274 | -130,573 | 1,176 | -91,048 | ,294 | 1,126 | 2,073 | ,674 | 2,494 | 74,173 |
| URC\_Org\_1 | ,192 | ,015 | -7,358 | ,066 | -5,131 | ,018 | ,063 | ,122 | ,022 | ,062 | ,385 | ,114 |
| URC\_Flex\_1 | ,463 | ,037 | -17,787 | ,160 | -12,403 | ,040 | ,191 | ,296 | ,041 | ,115 | ,931 | ,133 | ,562 |
| URC\_Flu\_1 | 1,105 | ,089 | -42,455 | ,382 | -29,603 | ,096 | ,366 | ,655 | ,097 | ,360 | 1,923 | ,318 | ,769 | 2,040 |

##### Implied Correlations (Group number 1 - Default model)

|  | Creativity\_TCTDP | MathKnowledge\_Cito | InhibitionIncongruentRT | UpdatingCombined | MeanRTShift | URC\_Org\_4 | URC\_Flex\_4 | URC\_Flu\_4 | URC\_Org\_3 | URC\_Flex\_3 | URC\_Flu\_3 | URC\_Org\_1 | URC\_Flex\_1 | URC\_Flu\_1 |
| --- | --- | --- | --- | --- | --- | --- | --- | --- | --- | --- | --- | --- | --- | --- |
| Creativity\_TCTDP | 1,000 |
| MathKnowledge\_Cito | ,000 | 1,000 |
| InhibitionIncongruentRT | ,000 | ,000 | 1,000 |
| UpdatingCombined | ,000 | ,000 | -,376 | 1,000 |
| MeanRTShift | ,000 | ,000 | ,333 | -,085 | 1,000 |
| URC\_Org\_4 | ,053 | ,041 | -,128 | ,209 | -,057 | 1,000 |
| URC\_Flex\_4 | ,059 | ,046 | -,143 | ,235 | -,064 | ,580 | 1,000 |
| URC\_Flu\_4 | ,072 | ,056 | -,175 | ,287 | -,078 | ,709 | ,796 | 1,000 |
| URC\_Org\_3 | ,068 | ,053 | -,165 | ,270 | -,074 | ,170 | ,217 | ,266 | 1,000 |
| URC\_Flex\_3 | ,068 | ,052 | -,165 | ,270 | -,074 | ,193 | ,235 | ,265 | ,563 | 1,000 |
| URC\_Flu\_3 | ,041 | ,032 | -,100 | ,164 | -,045 | ,117 | ,132 | ,154 | ,342 | ,342 | 1,000 |
| URC\_Org\_1 | ,059 | ,046 | -,144 | ,236 | -,064 | ,186 | ,190 | ,232 | ,288 | ,218 | ,132 | 1,000 |
| URC\_Flex\_1 | ,065 | ,050 | -,156 | ,256 | -,070 | ,184 | ,257 | ,252 | ,238 | ,180 | ,144 | ,526 | 1,000 |
| URC\_Flu\_1 | ,081 | ,062 | -,196 | ,321 | -,088 | ,230 | ,259 | ,293 | ,298 | ,297 | ,156 | ,660 | ,718 | 1,000 |

##### Implied Means (Group number 1 - Default model)

|  | Creativity\_TCTDP | MathKnowledge\_Cito | InhibitionIncongruentRT | UpdatingCombined | MeanRTShift | URC\_Org\_4 | URC\_Flex\_4 | URC\_Flu\_4 | URC\_Org\_3 | URC\_Flex\_3 | URC\_Flu\_3 | URC\_Org\_1 | URC\_Flex\_1 | URC\_Flu\_1 |
| --- | --- | --- | --- | --- | --- | --- | --- | --- | --- | --- | --- | --- | --- | --- |
|  | 20,331 | ,006 | 790,082 | ,036 | 1386,833 | ,498 | 1,777 | 2,306 | ,417 | 2,255 | 8,331 | ,551 | 1,705 | 2,367 |

##### Residual Covariances (Group number 1 - Default model)

|  | Creativity\_TCTDP | MathKnowledge\_Cito | InhibitionIncongruentRT | UpdatingCombined | MeanRTShift | URC\_Org\_4 | URC\_Flex\_4 | URC\_Flu\_4 | URC\_Org\_3 | URC\_Flex\_3 | URC\_Flu\_3 | URC\_Org\_1 | URC\_Flex\_1 | URC\_Flu\_1 |
| --- | --- | --- | --- | --- | --- | --- | --- | --- | --- | --- | --- | --- | --- | --- |
| Creativity\_TCTDP | ,000 |
| MathKnowledge\_Cito | ,160 | ,000 |
| InhibitionIncongruentRT | -94,518 | -9,985 | ,000 |
| UpdatingCombined | 1,802 | ,158 | ,000 | ,000 |
| MeanRTShift | -208,923 | -20,132 | ,000 | ,000 | ,000 |
| URC\_Org\_4 | ,260 | ,016 | 2,762 | -,025 | -,053 | ,001 |
| URC\_Flex\_4 | ,528 | ,104 | -1,123 | ,007 | 1,145 | ,001 | ,011 |
| URC\_Flu\_4 | 1,142 | ,077 | 2,821 | -,042 | 1,073 | ,005 | ,016 | ,028 |
| URC\_Org\_3 | ,094 | ,001 | -2,277 | ,007 | -,391 | -,001 | ,016 | ,004 | ,001 |
| URC\_Flex\_3 | ,314 | ,089 | 2,117 | ,016 | 3,806 | -,010 | ,043 | ,030 | ,007 | ,011 |
| URC\_Flu\_3 | 4,236 | ,162 | -205,097 | 1,251 | -95,975 | ,060 | ,592 | ,753 | -,047 | -,082 | ,494 |
| URC\_Org\_1 | -,035 | ,007 | 2,689 | -,010 | ,566 | ,003 | -,002 | ,014 | -,002 | -,013 | ,229 | ,001 |
| URC\_Flex\_1 | ,872 | ,083 | -9,160 | ,064 | -2,314 | ,011 | ,028 | ,050 | -,004 | -,014 | ,685 | ,001 | ,013 |
| URC\_Flu\_1 | 1,025 | ,092 | 7,103 | ,044 | -12,001 | ,025 | ,075 | ,132 | -,008 | -,065 | 1,078 | ,006 | ,019 | ,027 |

##### Residual Means (Group number 1 - Default model)

|  | Creativity\_TCTDP | MathKnowledge\_Cito | InhibitionIncongruentRT | UpdatingCombined | MeanRTShift | URC\_Org\_4 | URC\_Flex\_4 | URC\_Flu\_4 | URC\_Org\_3 | URC\_Flex\_3 | URC\_Flu\_3 | URC\_Org\_1 | URC\_Flex\_1 | URC\_Flu\_1 |
| --- | --- | --- | --- | --- | --- | --- | --- | --- | --- | --- | --- | --- | --- | --- |
|  | ,000 | ,000 | ,000 | ,000 | ,000 | ,000 | ,000 | ,000 | ,000 | ,000 | ,000 | ,000 | ,000 | ,000 |

##### Standardized Residual Covariances (Group number 1 - Default model)

|  | Creativity\_TCTDP | MathKnowledge\_Cito | InhibitionIncongruentRT | UpdatingCombined | MeanRTShift | URC\_Org\_4 | URC\_Flex\_4 | URC\_Flu\_4 | URC\_Org\_3 | URC\_Flex\_3 | URC\_Flu\_3 | URC\_Org\_1 | URC\_Flex\_1 | URC\_Flu\_1 |
| --- | --- | --- | --- | --- | --- | --- | --- | --- | --- | --- | --- | --- | --- | --- |
| Creativity\_TCTDP | ,000 |
| MathKnowledge\_Cito | ,279 | ,000 |
| InhibitionIncongruentRT | -1,085 | -1,098 | ,000 |
| UpdatingCombined | 3,765 | 3,153 | ,000 | ,000 |
| MeanRTShift | -1,540 | -1,421 | ,000 | ,000 | ,000 |
| URC\_Org\_4 | 1,553 | ,934 | 1,036 | -1,701 | -,013 | ,099 |
| URC\_Flex\_4 | ,926 | 1,754 | -,123 | ,138 | ,081 | ,039 | ,128 |
| URC\_Flu\_4 | 1,266 | ,815 | ,195 | -,521 | ,048 | ,135 | ,135 | ,135 |
| URC\_Org\_3 | ,712 | ,077 | -1,078 | ,564 | -,120 | -,343 | 1,113 | ,184 | ,172 |
| URC\_Flex\_3 | ,643 | 1,738 | ,271 | ,359 | ,316 | -,665 | ,823 | ,365 | ,554 | ,179 |
| URC\_Flu\_3 | ,855 | ,313 | -2,602 | 2,864 | -,785 | ,397 | 1,144 | ,919 | -,374 | -,176 | ,078 |
| URC\_Org\_1 | -,180 | ,352 | ,866 | -,550 | ,118 | ,467 | -,118 | ,433 | -,441 | -,711 | 1,300 | ,083 |
| URC\_Flex\_1 | 2,020 | 1,853 | -1,325 | 1,653 | -,217 | ,837 | ,600 | ,682 | -,332 | -,364 | 1,747 | ,031 | ,262 |
| URC\_Flu\_1 | 1,245 | 1,072 | ,536 | ,584 | -,590 | ,965 | ,859 | ,946 | -,376 | -,861 | 1,441 | ,163 | ,244 | ,156 |

##### Standardized Residual Means (Group number 1 - Default model)

|  | Creativity\_TCTDP | MathKnowledge\_Cito | InhibitionIncongruentRT | UpdatingCombined | MeanRTShift | URC\_Org\_4 | URC\_Flex\_4 | URC\_Flu\_4 | URC\_Org\_3 | URC\_Flex\_3 | URC\_Flu\_3 | URC\_Org\_1 | URC\_Flex\_1 | URC\_Flu\_1 |
| --- | --- | --- | --- | --- | --- | --- | --- | --- | --- | --- | --- | --- | --- | --- |
|  | ,000 | ,000 | ,000 | ,000 | ,000 | ,000 | ,000 | ,000 | ,000 | ,000 | ,000 | ,000 | ,000 | ,000 |

##### Total Effects (Group number 1 - Default model)

|  | Creativity\_TCTDP | MathKnowledge\_Cito | InhibitionIncongruentRT | UpdatingCombined | MeanRTShift | Math\_DT | Task\_4 | Task\_3 | Task\_1 |
| --- | --- | --- | --- | --- | --- | --- | --- | --- | --- |
| Math\_DT | ,012 | ,089 | -,001 | ,497 | ,000 | ,000 | ,000 | ,000 | ,000 |
| Task\_4 | ,012 | ,087 | -,001 | ,486 | ,000 | ,978 | ,000 | ,000 | ,000 |
| Task\_3 | ,037 | ,275 | -,002 | 1,530 | -,001 | 3,076 | ,000 | ,000 | ,000 |
| Task\_1 | ,012 | ,089 | -,001 | ,497 | ,000 | 1,000 | ,000 | ,000 | ,000 |
| URC\_Org\_4 | ,002 | ,012 | ,000 | ,066 | ,000 | ,132 | ,135 | ,000 | ,000 |
| URC\_Flex\_4 | ,006 | ,045 | ,000 | ,252 | ,000 | ,507 | ,518 | ,000 | ,000 |
| URC\_Flu\_4 | ,012 | ,087 | -,001 | ,486 | ,000 | ,978 | 1,000 | ,000 | ,000 |
| URC\_Org\_3 | ,002 | ,012 | ,000 | ,067 | ,000 | ,135 | ,000 | ,044 | ,000 |
| URC\_Flex\_3 | ,006 | ,045 | ,000 | ,248 | ,000 | ,499 | ,000 | ,162 | ,000 |
| URC\_Flu\_3 | ,037 | ,275 | -,002 | 1,530 | -,001 | 3,076 | ,000 | 1,000 | ,000 |
| URC\_Org\_1 | ,002 | ,015 | ,000 | ,086 | ,000 | ,173 | ,000 | ,000 | ,173 |
| URC\_Flex\_1 | ,005 | ,037 | ,000 | ,208 | ,000 | ,419 | ,000 | ,000 | ,419 |
| URC\_Flu\_1 | ,012 | ,089 | -,001 | ,497 | ,000 | 1,000 | ,000 | ,000 | 1,000 |

##### Standardized Total Effects (Group number 1 - Default model)

|  | Creativity\_TCTDP | MathKnowledge\_Cito | InhibitionIncongruentRT | UpdatingCombined | MeanRTShift | Math\_DT | Task\_4 | Task\_3 | Task\_1 |
| --- | --- | --- | --- | --- | --- | --- | --- | --- | --- |
| Math\_DT | ,136 | ,105 | -,125 | ,488 | -,065 | ,000 | ,000 | ,000 | ,000 |
| Task\_4 | ,073 | ,057 | -,067 | ,262 | -,035 | ,538 | ,000 | ,000 | ,000 |
| Task\_3 | ,091 | ,070 | -,083 | ,325 | -,043 | ,666 | ,000 | ,000 | ,000 |
| Task\_1 | ,085 | ,066 | -,078 | ,306 | -,041 | ,627 | ,000 | ,000 | ,000 |
| URC\_Org\_4 | ,053 | ,041 | -,048 | ,189 | -,025 | ,387 | ,719 | ,000 | ,000 |
| URC\_Flex\_4 | ,059 | ,046 | -,054 | ,212 | -,028 | ,435 | ,807 | ,000 | ,000 |
| URC\_Flu\_4 | ,072 | ,056 | -,066 | ,259 | -,034 | ,531 | ,986 | ,000 | ,000 |
| URC\_Org\_3 | ,068 | ,053 | -,062 | ,244 | -,032 | ,500 | ,000 | ,751 | ,000 |
| URC\_Flex\_3 | ,068 | ,052 | -,062 | ,244 | -,032 | ,500 | ,000 | ,750 | ,000 |
| URC\_Flu\_3 | ,041 | ,032 | -,038 | ,148 | -,020 | ,304 | ,000 | ,455 | ,000 |
| URC\_Org\_1 | ,059 | ,046 | -,054 | ,213 | -,028 | ,436 | ,000 | ,000 | ,696 |
| URC\_Flex\_1 | ,065 | ,050 | -,059 | ,232 | -,031 | ,475 | ,000 | ,000 | ,757 |
| URC\_Flu\_1 | ,081 | ,062 | -,074 | ,290 | -,039 | ,595 | ,000 | ,000 | ,948 |

##### Direct Effects (Group number 1 - Default model)

|  | Creativity\_TCTDP | MathKnowledge\_Cito | InhibitionIncongruentRT | UpdatingCombined | MeanRTShift | Math\_DT | Task\_4 | Task\_3 | Task\_1 |
| --- | --- | --- | --- | --- | --- | --- | --- | --- | --- |
| Math\_DT | ,012 | ,089 | -,001 | ,497 | ,000 | ,000 | ,000 | ,000 | ,000 |
| Task\_4 | ,000 | ,000 | ,000 | ,000 | ,000 | ,978 | ,000 | ,000 | ,000 |
| Task\_3 | ,000 | ,000 | ,000 | ,000 | ,000 | 3,076 | ,000 | ,000 | ,000 |
| Task\_1 | ,000 | ,000 | ,000 | ,000 | ,000 | 1,000 | ,000 | ,000 | ,000 |
| URC\_Org\_4 | ,000 | ,000 | ,000 | ,000 | ,000 | ,000 | ,135 | ,000 | ,000 |
| URC\_Flex\_4 | ,000 | ,000 | ,000 | ,000 | ,000 | ,000 | ,518 | ,000 | ,000 |
| URC\_Flu\_4 | ,000 | ,000 | ,000 | ,000 | ,000 | ,000 | 1,000 | ,000 | ,000 |
| URC\_Org\_3 | ,000 | ,000 | ,000 | ,000 | ,000 | ,000 | ,000 | ,044 | ,000 |
| URC\_Flex\_3 | ,000 | ,000 | ,000 | ,000 | ,000 | ,000 | ,000 | ,162 | ,000 |
| URC\_Flu\_3 | ,000 | ,000 | ,000 | ,000 | ,000 | ,000 | ,000 | 1,000 | ,000 |
| URC\_Org\_1 | ,000 | ,000 | ,000 | ,000 | ,000 | ,000 | ,000 | ,000 | ,173 |
| URC\_Flex\_1 | ,000 | ,000 | ,000 | ,000 | ,000 | ,000 | ,000 | ,000 | ,419 |
| URC\_Flu\_1 | ,000 | ,000 | ,000 | ,000 | ,000 | ,000 | ,000 | ,000 | 1,000 |

##### Standardized Direct Effects (Group number 1 - Default model)

|  | Creativity\_TCTDP | MathKnowledge\_Cito | InhibitionIncongruentRT | UpdatingCombined | MeanRTShift | Math\_DT | Task\_4 | Task\_3 | Task\_1 |
| --- | --- | --- | --- | --- | --- | --- | --- | --- | --- |
| Math\_DT | ,136 | ,105 | -,125 | ,488 | -,065 | ,000 | ,000 | ,000 | ,000 |
| Task\_4 | ,000 | ,000 | ,000 | ,000 | ,000 | ,538 | ,000 | ,000 | ,000 |
| Task\_3 | ,000 | ,000 | ,000 | ,000 | ,000 | ,666 | ,000 | ,000 | ,000 |
| Task\_1 | ,000 | ,000 | ,000 | ,000 | ,000 | ,627 | ,000 | ,000 | ,000 |
| URC\_Org\_4 | ,000 | ,000 | ,000 | ,000 | ,000 | ,000 | ,719 | ,000 | ,000 |
| URC\_Flex\_4 | ,000 | ,000 | ,000 | ,000 | ,000 | ,000 | ,807 | ,000 | ,000 |
| URC\_Flu\_4 | ,000 | ,000 | ,000 | ,000 | ,000 | ,000 | ,986 | ,000 | ,000 |
| URC\_Org\_3 | ,000 | ,000 | ,000 | ,000 | ,000 | ,000 | ,000 | ,751 | ,000 |
| URC\_Flex\_3 | ,000 | ,000 | ,000 | ,000 | ,000 | ,000 | ,000 | ,750 | ,000 |
| URC\_Flu\_3 | ,000 | ,000 | ,000 | ,000 | ,000 | ,000 | ,000 | ,455 | ,000 |
| URC\_Org\_1 | ,000 | ,000 | ,000 | ,000 | ,000 | ,000 | ,000 | ,000 | ,696 |
| URC\_Flex\_1 | ,000 | ,000 | ,000 | ,000 | ,000 | ,000 | ,000 | ,000 | ,757 |
| URC\_Flu\_1 | ,000 | ,000 | ,000 | ,000 | ,000 | ,000 | ,000 | ,000 | ,948 |

##### Indirect Effects (Group number 1 - Default model)

|  | Creativity\_TCTDP | MathKnowledge\_Cito | InhibitionIncongruentRT | UpdatingCombined | MeanRTShift | Math\_DT | Task\_4 | Task\_3 | Task\_1 |
| --- | --- | --- | --- | --- | --- | --- | --- | --- | --- |
| Math\_DT | ,000 | ,000 | ,000 | ,000 | ,000 | ,000 | ,000 | ,000 | ,000 |
| Task\_4 | ,012 | ,087 | -,001 | ,486 | ,000 | ,000 | ,000 | ,000 | ,000 |
| Task\_3 | ,037 | ,275 | -,002 | 1,530 | -,001 | ,000 | ,000 | ,000 | ,000 |
| Task\_1 | ,012 | ,089 | -,001 | ,497 | ,000 | ,000 | ,000 | ,000 | ,000 |
| URC\_Org\_4 | ,002 | ,012 | ,000 | ,066 | ,000 | ,132 | ,000 | ,000 | ,000 |
| URC\_Flex\_4 | ,006 | ,045 | ,000 | ,252 | ,000 | ,507 | ,000 | ,000 | ,000 |
| URC\_Flu\_4 | ,012 | ,087 | -,001 | ,486 | ,000 | ,978 | ,000 | ,000 | ,000 |
| URC\_Org\_3 | ,002 | ,012 | ,000 | ,067 | ,000 | ,135 | ,000 | ,000 | ,000 |
| URC\_Flex\_3 | ,006 | ,045 | ,000 | ,248 | ,000 | ,499 | ,000 | ,000 | ,000 |
| URC\_Flu\_3 | ,037 | ,275 | -,002 | 1,530 | -,001 | 3,076 | ,000 | ,000 | ,000 |
| URC\_Org\_1 | ,002 | ,015 | ,000 | ,086 | ,000 | ,173 | ,000 | ,000 | ,000 |
| URC\_Flex\_1 | ,005 | ,037 | ,000 | ,208 | ,000 | ,419 | ,000 | ,000 | ,000 |
| URC\_Flu\_1 | ,012 | ,089 | -,001 | ,497 | ,000 | 1,000 | ,000 | ,000 | ,000 |

##### Standardized Indirect Effects (Group number 1 - Default model)

|  | Creativity\_TCTDP | MathKnowledge\_Cito | InhibitionIncongruentRT | UpdatingCombined | MeanRTShift | Math\_DT | Task\_4 | Task\_3 | Task\_1 |
| --- | --- | --- | --- | --- | --- | --- | --- | --- | --- |
| Math\_DT | ,000 | ,000 | ,000 | ,000 | ,000 | ,000 | ,000 | ,000 | ,000 |
| Task\_4 | ,073 | ,057 | -,067 | ,262 | -,035 | ,000 | ,000 | ,000 | ,000 |
| Task\_3 | ,091 | ,070 | -,083 | ,325 | -,043 | ,000 | ,000 | ,000 | ,000 |
| Task\_1 | ,085 | ,066 | -,078 | ,306 | -,041 | ,000 | ,000 | ,000 | ,000 |
| URC\_Org\_4 | ,053 | ,041 | -,048 | ,189 | -,025 | ,387 | ,000 | ,000 | ,000 |
| URC\_Flex\_4 | ,059 | ,046 | -,054 | ,212 | -,028 | ,435 | ,000 | ,000 | ,000 |
| URC\_Flu\_4 | ,072 | ,056 | -,066 | ,259 | -,034 | ,531 | ,000 | ,000 | ,000 |
| URC\_Org\_3 | ,068 | ,053 | -,062 | ,244 | -,032 | ,500 | ,000 | ,000 | ,000 |
| URC\_Flex\_3 | ,068 | ,052 | -,062 | ,244 | -,032 | ,500 | ,000 | ,000 | ,000 |
| URC\_Flu\_3 | ,041 | ,032 | -,038 | ,148 | -,020 | ,304 | ,000 | ,000 | ,000 |
| URC\_Org\_1 | ,059 | ,046 | -,054 | ,213 | -,028 | ,436 | ,000 | ,000 | ,000 |
| URC\_Flex\_1 | ,065 | ,050 | -,059 | ,232 | -,031 | ,475 | ,000 | ,000 | ,000 |
| URC\_Flu\_1 | ,081 | ,062 | -,074 | ,290 | -,039 | ,595 | ,000 | ,000 | ,000 |

##### Modification Indices (Group number 1 - Default model)

##### Covariances: (Group number 1 - Default model)

|  |  |  | M.I. | Par Change |
| --- | --- | --- | --- | --- |
| e2 | <--> | e4 | 13,573 | 1,633 |
| e2 | <--> | e5 | 9,077 | ,139 |
| e9 | <--> | e2 | 4,073 | ,740 |
| e7 | <--> | e3 | 4,135 | -8,326 |

##### Variances: (Group number 1 - Default model)

|  |  |  | M.I. | Par Change |
| --- | --- | --- | --- | --- |

##### Regression Weights: (Group number 1 - Default model)

|  |  |  | M.I. | Par Change |
| --- | --- | --- | --- | --- |
| Creativity\_TCTDP | <--- | UpdatingCombined | 14,146 | 2,591 |
| MathKnowledge\_Cito | <--- | UpdatingCombined | 9,925 | ,227 |
| UpdatingCombined | <--- | MathKnowledge\_Cito | 9,077 | ,140 |
| URC\_Flu\_3 | <--- | UpdatingCombined | 7,984 | 1,611 |

##### Means: (Group number 1 - Default model)

|  |  |  | M.I. | Par Change |
| --- | --- | --- | --- | --- |

##### Intercepts: (Group number 1 - Default model)

|  |  |  | M.I. | Par Change |
| --- | --- | --- | --- | --- |

##### Minimization History (Default model)

| Iteration |  | Negative eigenvalues | Condition # | Smallest eigenvalue | Diameter | F | NTries | Ratio |
| --- | --- | --- | --- | --- | --- | --- | --- | --- |
| 0 | e | 9 |  | -,374 | 9999,000 | 1610,527 | 0 | 9999,000 |
| 1 | e | 8 |  | -,378 | 1,597 | 792,656 | 19 | ,680 |
| 2 | e | 2 |  | -,054 | ,908 | 329,435 | 4 | ,831 |
| 3 | e | 1 |  | -,015 | ,270 | 241,555 | 5 | ,823 |
| 4 | e | 0 | 2738516,139 |  | ,879 | 154,994 | 8 | ,734 |
| 5 | e | 0 | 3032375,175 |  | ,880 | 121,046 | 2 | ,000 |
| 6 | e | 0 | 3270460,035 |  | ,383 | 105,320 | 2 | ,000 |
| 7 | e | 0 | 3313040,210 |  | ,308 | 97,902 | 1 | 1,135 |
| 8 | e | 0 | 3396066,173 |  | ,131 | 97,331 | 1 | 1,083 |
| 9 | e | 0 | 3415681,361 |  | ,036 | 97,316 | 1 | 1,029 |
| 10 | e | 0 | 3533543,178 |  | ,002 | 97,316 | 1 | 1,002 |
| 11 | e | 0 | 3532438,937 |  | ,000 | 97,316 | 1 | 1,000 |

##### Pairwise Parameter Comparisons (Default model)

##### Variance-covariance Matrix of Estimates (Default model)

|  | par\_1 | par\_2 | par\_3 | par\_4 | par\_5 | par\_6 | par\_7 | par\_8 | par\_9 | par\_10 | par\_11 | par\_12 | par\_13 | par\_14 | par\_15 | par\_16 | par\_17 | par\_18 | par\_19 | par\_20 | par\_21 | par\_22 | par\_23 | par\_24 | par\_25 | par\_26 | par\_27 | par\_28 | par\_29 | par\_30 | par\_31 | par\_32 | par\_33 | par\_34 | par\_35 | par\_36 | par\_37 | par\_38 | par\_39 | par\_40 | par\_41 | par\_42 | par\_43 | par\_44 | par\_45 | par\_46 | par\_47 | par\_48 | par\_49 | par\_50 | par\_51 | par\_52 | par\_53 | par\_54 | par\_55 | par\_56 | par\_57 |
| --- | --- | --- | --- | --- | --- | --- | --- | --- | --- | --- | --- | --- | --- | --- | --- | --- | --- | --- | --- | --- | --- | --- | --- | --- | --- | --- | --- | --- | --- | --- | --- | --- | --- | --- | --- | --- | --- | --- | --- | --- | --- | --- | --- | --- | --- | --- | --- | --- | --- | --- | --- | --- | --- | --- | --- | --- | --- |
| par\_1 | ,504 |
| par\_2 | ,041 | ,026 |
| par\_3 | ,001 | ,000 | ,001 |
| par\_4 | ,001 | ,000 | ,000 | ,000 |
| par\_5 | -,011 | ,000 | ,000 | ,000 | ,001 |
| par\_6 | -,003 | ,000 | ,000 | ,000 | ,000 | ,000 |
| par\_7 | ,000 | ,000 | ,000 | ,000 | ,000 | ,000 | ,001 |
| par\_8 | ,000 | ,000 | ,000 | ,000 | ,000 | ,000 | ,000 | ,000 |
| par\_9 | ,000 | ,000 | ,000 | ,000 | ,000 | ,000 | ,000 | ,000 | 5133924,250 |
| par\_10 | ,000 | ,000 | ,000 | ,000 | ,000 | ,000 | ,000 | ,000 | -3420,827 | 65,698 |
| par\_11 | ,000 | ,000 | ,000 | ,000 | ,000 | ,000 | ,000 | ,000 | -10263,402 | 32,698 | 140,655 |
| par\_12 | -,004 | -,001 | ,000 | ,000 | ,000 | ,000 | ,000 | ,000 | ,000 | ,000 | ,000 | ,004 |
| par\_13 | -,001 | ,000 | ,000 | ,000 | ,000 | ,000 | ,000 | ,000 | ,000 | ,000 | ,000 | ,000 | ,000 |
| par\_14 | ,000 | ,000 | ,000 | ,000 | ,000 | ,000 | ,000 | ,000 | ,000 | ,000 | ,000 | ,000 | ,000 | ,000 |
| par\_15 | ,000 | ,000 | ,000 | ,000 | ,000 | ,000 | ,000 | ,000 | ,000 | ,000 | ,000 | ,000 | ,000 | ,000 | ,000 |
| par\_16 | -,022 | -,007 | ,000 | ,000 | ,000 | ,000 | ,000 | ,000 | ,000 | ,000 | ,000 | -,001 | ,000 | ,000 | ,000 | ,009 |
| par\_17 | ,012 | ,005 | ,002 | ,001 | ,000 | ,000 | ,000 | ,000 | ,000 | ,000 | ,000 | ,001 | ,000 | ,000 | ,000 | -,003 | ,174 |
| par\_18 | ,004 | ,000 | ,000 | ,000 | ,000 | ,000 | ,000 | ,000 | ,000 | ,000 | ,000 | ,000 | ,000 | ,000 | ,000 | ,000 | ,001 | ,002 |
| par\_19 | -,005 | -,004 | ,000 | ,000 | ,000 | ,000 | ,001 | ,000 | ,000 | ,000 | ,000 | ,001 | ,000 | ,000 | ,000 | -,001 | ,005 | ,000 | ,155 |
| par\_20 | -,001 | ,000 | ,000 | ,000 | ,000 | ,000 | ,000 | ,000 | ,000 | ,000 | ,000 | ,000 | ,000 | ,000 | ,000 | ,000 | -,001 | ,000 | ,000 | ,000 |
| par\_21 | -,001 | ,000 | ,000 | ,000 | ,000 | ,000 | ,000 | ,000 | ,000 | ,000 | ,000 | ,000 | ,000 | ,000 | ,000 | ,000 | ,000 | ,000 | ,000 | ,000 | ,000 |
| par\_22 | ,000 | ,000 | ,000 | ,000 | ,000 | ,000 | ,000 | ,000 | ,000 | ,000 | ,000 | ,000 | ,000 | ,000 | ,000 | ,000 | ,000 | ,000 | -,001 | ,000 | ,000 | ,001 |
| par\_23 | ,000 | ,000 | ,000 | ,000 | ,000 | ,000 | ,000 | ,000 | ,000 | ,000 | ,000 | ,000 | ,000 | ,000 | ,000 | ,000 | ,000 | ,000 | ,000 | ,000 | ,000 | ,000 | ,000 |
| par\_24 | ,000 | ,000 | ,000 | ,000 | ,000 | ,000 | ,000 | ,000 | ,000 | ,000 | ,000 | ,000 | ,000 | ,000 | ,000 | ,000 | ,000 | ,000 | ,000 | ,000 | ,000 | ,000 | ,000 | ,000 |
| par\_25 | ,000 | ,000 | ,000 | ,000 | ,000 | ,000 | ,000 | ,000 | ,000 | ,000 | ,000 | ,000 | ,000 | ,000 | ,000 | ,000 | ,000 | ,000 | ,000 | ,000 | ,000 | ,000 | ,000 | ,000 | ,000 |
| par\_26 | ,000 | ,000 | ,000 | ,000 | ,000 | ,000 | ,000 | ,000 | ,000 | ,000 | ,000 | ,000 | ,000 | ,000 | ,000 | ,000 | ,000 | ,000 | ,000 | ,000 | ,000 | ,000 | ,000 | ,000 | ,000 | 201,189 |
| par\_27 | ,000 | ,000 | ,000 | ,000 | ,000 | ,000 | ,000 | ,000 | ,000 | ,000 | ,000 | ,000 | ,000 | ,000 | ,000 | ,000 | ,000 | ,000 | ,000 | ,000 | ,000 | ,000 | ,000 | ,000 | ,000 | -,060 | ,003 |
| par\_28 | ,000 | ,000 | ,000 | ,000 | ,000 | ,000 | ,000 | ,000 | ,000 | ,000 | ,000 | ,000 | ,000 | ,000 | ,000 | ,000 | ,000 | ,000 | ,000 | ,000 | ,000 | ,000 | ,000 | ,000 | ,000 | 42,998 | -,171 | 82,933 |
| par\_29 | ,000 | ,000 | ,000 | ,000 | ,000 | ,000 | ,000 | ,000 | ,000 | ,000 | ,000 | ,000 | ,000 | ,000 | ,000 | ,000 | ,000 | ,000 | ,000 | ,000 | ,000 | ,000 | ,000 | ,000 | ,000 | ,000 | ,000 | ,000 | ,330 |
| par\_30 | ,000 | ,000 | ,000 | ,000 | ,000 | ,000 | ,000 | ,000 | ,000 | ,000 | ,000 | ,000 | ,000 | ,000 | ,000 | ,000 | ,000 | ,000 | ,000 | ,000 | ,000 | ,000 | ,000 | ,000 | ,000 | ,000 | ,000 | ,000 | ,000 | ,004 |
| par\_31 | -,015 | -,007 | ,000 | ,000 | ,000 | ,000 | ,000 | ,000 | ,000 | ,000 | ,000 | -,001 | -,001 | ,000 | ,000 | -,004 | -,007 | ,000 | -,003 | ,000 | ,000 | ,000 | ,000 | ,000 | ,000 | ,000 | ,000 | ,000 | ,000 | ,000 | ,205 |
| par\_32 | -,005 | -,003 | ,000 | ,000 | ,000 | ,000 | ,000 | ,000 | ,000 | ,000 | ,000 | ,000 | ,000 | ,000 | ,000 | -,002 | -,002 | ,000 | -,001 | ,000 | ,000 | ,000 | ,000 | ,000 | ,000 | ,000 | ,000 | ,000 | ,000 | ,000 | ,085 | ,037 |
| par\_33 | -,002 | -,001 | ,000 | ,000 | ,000 | ,000 | ,000 | ,000 | ,000 | ,000 | ,000 | ,000 | ,000 | ,000 | ,000 | -,001 | -,001 | ,000 | -,001 | ,000 | ,000 | ,000 | ,000 | ,000 | ,000 | ,000 | ,000 | ,000 | ,000 | ,000 | ,035 | ,015 | ,006 |
| par\_34 | ,263 | ,003 | ,000 | ,000 | -,008 | -,002 | ,000 | ,000 | ,000 | ,000 | ,000 | -,006 | -,003 | ,000 | ,000 | -,024 | -,015 | ,002 | -,013 | ,000 | ,000 | ,000 | ,000 | ,000 | ,000 | ,000 | ,000 | ,000 | ,000 | ,000 | ,605 | ,254 | ,105 | 2,269 |
| par\_35 | ,022 | ,001 | ,000 | ,000 | ,000 | ,000 | ,000 | ,000 | ,000 | ,000 | ,000 | -,001 | ,000 | ,000 | ,000 | -,004 | -,002 | ,000 | -,002 | ,000 | ,000 | ,000 | ,000 | ,000 | ,000 | ,000 | ,000 | ,000 | ,000 | ,000 | ,098 | ,041 | ,017 | ,319 | ,053 |
| par\_36 | ,006 | ,000 | ,000 | ,000 | ,000 | ,000 | ,000 | ,000 | ,000 | ,000 | ,000 | ,000 | ,000 | ,000 | ,000 | -,001 | -,001 | ,000 | ,000 | ,000 | ,000 | ,000 | ,000 | ,000 | ,000 | ,000 | ,000 | ,000 | ,000 | ,000 | ,027 | ,011 | ,005 | ,087 | ,014 | ,004 |
| par\_37 | ,011 | ,009 | ,000 | ,000 | ,000 | ,000 | ,000 | ,000 | ,000 | ,000 | ,000 | -,002 | -,001 | ,000 | ,000 | -,008 | -,004 | ,000 | -,006 | ,000 | ,000 | ,000 | ,000 | ,000 | ,000 | ,000 | ,000 | ,000 | ,000 | ,000 | ,191 | ,080 | ,033 | ,594 | ,096 | ,026 | ,199 |
| par\_38 | ,006 | ,004 | ,000 | ,000 | ,000 | ,000 | ,000 | ,000 | ,000 | ,000 | ,000 | -,001 | ,000 | ,000 | ,000 | -,004 | -,002 | ,000 | -,002 | ,000 | ,000 | ,000 | ,000 | ,000 | ,000 | ,000 | ,000 | ,000 | ,000 | ,000 | ,099 | ,042 | ,017 | ,308 | ,050 | ,013 | ,103 | ,055 |
| par\_39 | ,001 | ,001 | ,000 | ,000 | ,000 | ,000 | ,000 | ,000 | ,000 | ,000 | ,000 | ,000 | ,000 | ,000 | ,000 | -,001 | -,001 | ,000 | -,001 | ,000 | ,000 | ,000 | ,000 | ,000 | ,000 | ,000 | ,000 | ,000 | ,000 | ,000 | ,026 | ,011 | ,004 | ,080 | ,013 | ,004 | ,027 | ,014 | ,004 |
| par\_40 | ,000 | ,000 | ,000 | ,000 | ,000 | ,000 | ,000 | ,000 | 4792475,901 | -1431,271 | -6697,019 | ,000 | ,000 | ,000 | ,000 | ,000 | ,000 | ,000 | ,000 | ,000 | ,000 | ,000 | ,000 | ,000 | ,000 | ,000 | ,000 | ,000 | ,000 | ,000 | ,000 | ,000 | ,000 | ,000 | ,000 | ,000 | ,000 | ,000 | ,000 | 22424333,716 |
| par\_41 | ,000 | ,000 | ,000 | ,000 | ,000 | ,000 | ,000 | ,000 | 5,703 | -,238 | -,083 | ,000 | ,000 | ,000 | ,000 | ,000 | ,000 | ,000 | ,000 | ,000 | ,000 | ,000 | ,000 | ,000 | ,000 | ,000 | ,000 | ,000 | ,000 | ,000 | ,000 | ,000 | ,000 | ,000 | ,000 | ,000 | ,000 | ,000 | ,000 | 2,000 | ,003 |
| par\_42 | ,000 | ,000 | ,000 | ,000 | ,000 | ,000 | ,000 | ,000 | 1975522,993 | -7871,436 | -4081,053 | ,000 | ,000 | ,000 | ,000 | ,000 | ,000 | ,000 | ,000 | ,000 | ,000 | ,000 | ,000 | ,000 | ,000 | ,000 | ,000 | ,000 | ,000 | ,000 | ,000 | ,000 | ,000 | ,000 | ,000 | ,000 | ,000 | ,000 | ,000 | 1024236,687 | 16,261 | 3810341,053 |
| par\_43 | ,000 | ,000 | ,000 | ,000 | ,000 | ,000 | ,000 | ,000 | ,000 | ,000 | ,000 | ,000 | ,000 | ,000 | ,000 | ,000 | ,000 | ,000 | ,000 | ,000 | ,000 | ,000 | ,000 | ,000 | ,000 | ,000 | ,000 | ,000 | ,000 | ,000 | ,000 | ,000 | ,000 | ,000 | ,000 | ,000 | ,000 | ,000 | ,000 | ,000 | ,000 | ,000 | ,007 |
| par\_44 | ,000 | ,000 | ,000 | ,000 | ,000 | ,000 | ,000 | ,000 | ,000 | ,000 | ,000 | ,000 | ,000 | ,000 | ,000 | ,000 | ,000 | ,000 | ,000 | ,000 | ,000 | ,000 | ,000 | ,000 | ,000 | ,000 | ,000 | ,000 | ,000 | ,000 | ,000 | ,000 | ,000 | ,000 | ,000 | ,000 | ,000 | ,000 | ,000 | ,000 | ,000 | ,000 | ,000 | 60,367 |
| par\_45 | -,050 | -,010 | ,000 | ,000 | ,000 | ,000 | ,000 | ,000 | ,000 | ,000 | ,000 | ,001 | ,000 | ,000 | ,000 | ,004 | -,004 | -,001 | -,001 | ,000 | ,000 | ,000 | ,000 | ,000 | ,000 | ,000 | ,000 | ,000 | ,000 | ,000 | ,004 | ,001 | ,001 | -,019 | -,003 | -,001 | -,003 | -,001 | ,000 | ,000 | ,000 | ,000 | ,000 | ,000 | ,016 |
| par\_46 | ,037 | ,007 | -,002 | -,001 | ,000 | ,000 | ,000 | ,000 | ,000 | ,000 | ,000 | -,001 | ,000 | ,000 | ,000 | -,003 | -,005 | ,000 | ,000 | ,000 | ,000 | ,000 | ,000 | ,000 | ,000 | ,000 | ,000 | ,000 | ,000 | ,000 | -,002 | -,002 | -,001 | ,017 | ,003 | ,001 | ,003 | ,001 | ,000 | ,000 | ,000 | ,000 | ,000 | ,000 | -,009 | ,032 |
| par\_47 | ,538 | -,021 | ,002 | ,000 | -,048 | -,013 | -,001 | ,000 | ,000 | ,000 | ,000 | ,002 | ,001 | ,000 | ,000 | ,021 | -,008 | -,005 | -,028 | ,001 | ,001 | ,001 | ,000 | ,000 | ,000 | ,000 | ,000 | ,000 | ,000 | ,000 | ,016 | ,008 | ,003 | ,380 | -,029 | -,009 | ,003 | ,001 | ,000 | ,000 | ,000 | ,000 | ,000 | ,000 | ,026 | -,054 | 6,979 |
| par\_48 | ,008 | -,009 | ,000 | ,000 | ,000 | ,000 | -,002 | ,000 | ,000 | ,000 | ,000 | ,000 | ,000 | ,000 | ,000 | ,002 | ,000 | ,000 | ,000 | ,000 | ,000 | ,000 | ,000 | ,000 | ,000 | ,000 | ,000 | ,000 | ,000 | ,000 | ,003 | ,001 | ,001 | ,015 | ,002 | ,001 | -,002 | -,002 | -,001 | ,000 | ,000 | ,000 | ,000 | ,000 | -,003 | ,000 | -,011 | ,046 |
| par\_49 | ,005 | ,001 | ,002 | ,001 | ,000 | ,000 | ,000 | ,000 | ,000 | ,000 | ,000 | ,000 | ,000 | ,000 | ,000 | -,001 | ,004 | ,001 | ,000 | ,000 | ,000 | ,000 | ,000 | ,000 | ,000 | ,000 | ,000 | ,000 | ,000 | ,000 | -,001 | ,001 | ,000 | ,002 | ,000 | ,000 | ,000 | ,000 | ,000 | ,000 | ,000 | ,000 | ,000 | ,000 | -,001 | -,008 | ,001 | ,001 | ,009 |
| par\_50 | ,000 | ,000 | ,000 | ,000 | ,000 | ,000 | ,000 | ,000 | ,000 | ,000 | ,000 | ,000 | ,000 | ,000 | ,000 | ,000 | ,000 | ,000 | ,000 | ,000 | ,000 | ,000 | ,000 | ,000 | ,000 | ,000 | ,000 | ,000 | ,000 | ,000 | ,000 | ,000 | ,000 | ,000 | ,000 | ,000 | ,000 | ,000 | ,000 | ,000 | ,000 | ,000 | ,000 | ,000 | ,000 | ,000 | ,000 | ,000 | ,000 | ,000 |
| par\_51 | -,001 | ,000 | ,000 | ,000 | ,000 | ,000 | ,000 | ,000 | ,000 | ,000 | ,000 | ,000 | ,000 | ,000 | ,000 | ,000 | -,001 | ,000 | ,000 | ,000 | ,000 | ,000 | ,000 | ,000 | ,000 | ,000 | ,000 | ,000 | ,000 | ,000 | ,000 | ,000 | ,000 | ,000 | ,000 | ,000 | ,000 | ,000 | ,000 | ,000 | ,000 | ,000 | ,000 | ,000 | ,000 | ,001 | ,000 | ,000 | -,001 | ,000 | ,001 |
| par\_52 | -,702 | ,001 | ,000 | -,001 | ,031 | ,009 | ,000 | ,000 | ,000 | ,000 | ,000 | ,005 | ,000 | ,000 | ,000 | -,003 | -,057 | -,003 | ,046 | ,002 | ,000 | -,003 | ,000 | ,000 | ,000 | ,000 | ,000 | ,000 | ,000 | ,000 | -,034 | -,014 | -,006 | -,534 | -,028 | -,007 | -,032 | -,017 | -,004 | ,000 | ,000 | ,000 | ,000 | ,000 | ,017 | -,010 | -2,191 | -,016 | -,004 | ,000 | ,001 | 30,327 |
| par\_53 | ,004 | ,000 | ,000 | ,000 | ,000 | ,000 | ,000 | ,000 | ,000 | ,000 | ,000 | ,000 | ,000 | ,000 | ,000 | ,000 | -,002 | ,000 | ,000 | ,000 | ,000 | ,000 | ,000 | ,000 | ,000 | ,000 | ,000 | ,000 | ,000 | ,000 | ,001 | ,000 | ,000 | ,005 | ,000 | ,000 | ,001 | ,000 | ,000 | ,000 | ,000 | ,000 | ,000 | ,000 | ,000 | ,000 | -,001 | ,000 | ,000 | ,000 | ,000 | -,018 | ,002 |
| par\_54 | ,000 | ,000 | ,000 | ,000 | ,000 | ,000 | ,000 | ,000 | ,000 | ,000 | ,000 | ,000 | ,000 | ,000 | ,000 | ,000 | ,000 | ,000 | ,000 | ,000 | ,000 | ,000 | ,000 | ,000 | ,000 | ,000 | ,000 | ,000 | ,000 | ,000 | ,000 | ,000 | ,000 | ,000 | ,000 | ,000 | ,000 | ,000 | ,000 | ,000 | ,000 | ,000 | ,000 | ,000 | ,000 | ,000 | ,000 | ,000 | ,000 | ,000 | ,000 | -,002 | ,000 | ,000 |
| par\_55 | ,000 | ,000 | ,000 | ,000 | ,000 | ,000 | -,001 | ,000 | ,000 | ,000 | ,000 | ,000 | ,000 | ,000 | ,000 | ,000 | ,000 | ,000 | -,001 | ,000 | ,000 | ,000 | ,000 | ,000 | ,000 | ,000 | ,000 | ,000 | ,000 | ,000 | ,000 | ,000 | ,000 | ,000 | ,000 | ,000 | ,000 | ,000 | ,000 | ,000 | ,000 | ,000 | ,000 | ,000 | ,000 | ,000 | ,001 | ,002 | ,000 | ,000 | ,000 | ,000 | ,000 | ,000 | ,002 |
| par\_56 | ,001 | ,000 | ,000 | ,000 | ,000 | ,000 | ,002 | ,000 | ,000 | ,000 | ,000 | ,000 | ,000 | ,000 | ,000 | ,000 | ,000 | ,001 | ,003 | ,000 | ,000 | ,000 | ,000 | ,000 | ,000 | ,000 | ,000 | ,000 | ,000 | ,000 | -,001 | ,000 | ,000 | -,001 | ,000 | ,000 | -,001 | ,001 | ,000 | ,000 | ,000 | ,000 | ,000 | ,000 | ,000 | ,000 | -,004 | -,009 | ,000 | ,000 | ,000 | ,000 | ,000 | ,000 | -,002 | ,009 |
| par\_57 | ,000 | ,000 | ,000 | ,000 | ,000 | ,000 | ,000 | ,000 | ,000 | ,000 | ,000 | ,000 | ,000 | ,000 | ,000 | ,000 | ,000 | ,000 | ,000 | ,000 | ,000 | ,000 | ,000 | ,000 | ,000 | ,000 | ,000 | ,000 | ,000 | ,000 | ,000 | ,000 | ,000 | ,000 | ,000 | ,000 | ,000 | ,000 | ,000 | ,000 | ,000 | ,000 | ,000 | ,000 | ,000 | ,000 | ,000 | ,000 | ,000 | ,000 | ,000 | ,000 | ,000 | ,000 | ,000 | ,000 | ,000 |

##### Correlations of Estimates (Default model)

|  | par\_1 | par\_2 | par\_3 | par\_4 | par\_5 | par\_6 | par\_7 | par\_8 | par\_9 | par\_10 | par\_11 | par\_12 | par\_13 | par\_14 | par\_15 | par\_16 | par\_17 | par\_18 | par\_19 | par\_20 | par\_21 | par\_22 | par\_23 | par\_24 | par\_25 | par\_26 | par\_27 | par\_28 | par\_29 | par\_30 | par\_31 | par\_32 | par\_33 | par\_34 | par\_35 | par\_36 | par\_37 | par\_38 | par\_39 | par\_40 | par\_41 | par\_42 | par\_43 | par\_44 | par\_45 | par\_46 | par\_47 | par\_48 | par\_49 | par\_50 | par\_51 | par\_52 | par\_53 | par\_54 | par\_55 | par\_56 | par\_57 |
| --- | --- | --- | --- | --- | --- | --- | --- | --- | --- | --- | --- | --- | --- | --- | --- | --- | --- | --- | --- | --- | --- | --- | --- | --- | --- | --- | --- | --- | --- | --- | --- | --- | --- | --- | --- | --- | --- | --- | --- | --- | --- | --- | --- | --- | --- | --- | --- | --- | --- | --- | --- | --- | --- | --- | --- | --- | --- |
| par\_1 | 1,000 |
| par\_2 | ,360 | 1,000 |
| par\_3 | ,066 | ,073 | 1,000 |
| par\_4 | ,087 | ,064 | ,393 | 1,000 |
| par\_5 | -,608 | ,005 | -,041 | -,036 | 1,000 |
| par\_6 | -,612 | -,014 | -,005 | ,007 | ,766 | 1,000 |
| par\_7 | ,008 | -,023 | ,046 | -,002 | ,003 | -,008 | 1,000 |
| par\_8 | -,003 | -,035 | ,006 | ,003 | -,001 | -,014 | ,367 | 1,000 |
| par\_9 | ,000 | ,000 | ,000 | ,000 | ,000 | ,000 | ,000 | ,000 | 1,000 |
| par\_10 | ,000 | ,000 | ,000 | ,000 | ,000 | ,000 | ,000 | ,000 | -,186 | 1,000 |
| par\_11 | ,000 | ,000 | ,000 | ,000 | ,000 | ,000 | ,000 | ,000 | -,382 | ,340 | 1,000 |
| par\_12 | -,094 | -,097 | ,017 | ,006 | ,040 | ,000 | ,043 | ,036 | ,000 | ,000 | ,000 | 1,000 |
| par\_13 | -,135 | -,112 | -,006 | -,014 | ,016 | ,017 | ,015 | ,013 | ,000 | ,000 | ,000 | ,056 | 1,000 |
| par\_14 | -,010 | ,058 | -,031 | -,022 | ,035 | ,008 | ,000 | ,000 | ,000 | ,000 | ,000 | -,035 | -,053 | 1,000 |
| par\_15 | ,115 | ,108 | ,051 | ,043 | -,003 | -,011 | ,005 | ,001 | ,000 | ,000 | ,000 | ,065 | ,063 | -,325 | 1,000 |
| par\_16 | -,330 | -,455 | -,075 | -,067 | -,005 | ,005 | -,015 | -,003 | ,000 | ,000 | ,000 | -,099 | -,109 | ,293 | -,144 | 1,000 |
| par\_17 | ,040 | ,069 | ,131 | ,095 | ,044 | -,003 | ,008 | ,007 | ,000 | ,000 | ,000 | ,025 | -,003 | ,018 | ,032 | -,083 | 1,000 |
| par\_18 | ,130 | -,007 | ,116 | ,077 | -,013 | -,015 | ,119 | ,106 | ,000 | ,000 | ,000 | ,043 | -,014 | -,037 | ,051 | -,059 | ,037 | 1,000 |
| par\_19 | -,019 | -,067 | ,005 | ,003 | ,029 | ,054 | ,085 | ,078 | ,000 | ,000 | ,000 | ,030 | ,011 | ,017 | ,001 | -,014 | ,033 | ,016 | 1,000 |
| par\_20 | -,058 | ,000 | -,054 | -,093 | -,017 | ,037 | -,006 | -,007 | ,000 | ,000 | ,000 | -,032 | -,010 | ,029 | -,036 | ,023 | -,134 | -,035 | -,016 | 1,000 |
| par\_21 | -,047 | ,008 | -,037 | -,029 | ,003 | ,004 | -,094 | -,091 | ,000 | ,000 | ,000 | -,027 | ,004 | ,015 | -,021 | ,021 | -,017 | -,408 | -,006 | ,038 | 1,000 |
| par\_22 | ,004 | ,000 | -,009 | -,004 | -,062 | -,041 | -,010 | -,011 | ,000 | ,000 | ,000 | -,027 | ,007 | ,001 | -,012 | ,013 | -,017 | -,111 | -,149 | ,093 | -,037 | 1,000 |
| par\_23 | ,014 | ,017 | -,012 | ,022 | ,003 | ,017 | -,006 | -,007 | ,000 | ,000 | ,000 | -,001 | ,011 | -,004 | -,001 | ,000 | -,097 | -,052 | ,000 | -,032 | ,030 | -,019 | 1,000 |
| par\_24 | -,030 | -,006 | -,029 | -,020 | ,004 | ,004 | ,000 | ,026 | ,000 | ,000 | ,000 | -,008 | ,002 | ,007 | -,011 | ,018 | -,009 | -,204 | ,009 | ,001 | ,075 | ,023 | -,029 | 1,000 |
| par\_25 | -,008 | ,006 | -,008 | -,002 | ,015 | ,035 | -,108 | -,074 | ,000 | ,000 | ,000 | -,023 | -,022 | -,006 | ,001 | ,022 | -,009 | -,031 | -,122 | ,023 | ,022 | -,004 | ,016 | ,111 | 1,000 |
| par\_26 | ,000 | ,000 | ,000 | ,000 | ,000 | ,000 | ,000 | ,000 | ,000 | ,000 | ,000 | ,000 | ,000 | ,000 | ,000 | ,000 | ,000 | ,000 | ,000 | ,000 | ,000 | ,000 | ,000 | ,000 | ,000 | 1,000 |
| par\_27 | ,000 | ,000 | ,000 | ,000 | ,000 | ,000 | ,000 | ,000 | ,000 | ,000 | ,000 | ,000 | ,000 | ,000 | ,000 | ,000 | ,000 | ,000 | ,000 | ,000 | ,000 | ,000 | ,000 | ,000 | ,000 | -,085 | 1,000 |
| par\_28 | ,000 | ,000 | ,000 | ,000 | ,000 | ,000 | ,000 | ,000 | ,000 | ,000 | ,000 | ,000 | ,000 | ,000 | ,000 | ,000 | ,000 | ,000 | ,000 | ,000 | ,000 | ,000 | ,000 | ,000 | ,000 | ,333 | -,376 | 1,000 |
| par\_29 | ,000 | ,000 | ,000 | ,000 | ,000 | ,000 | ,000 | ,000 | ,000 | ,000 | ,000 | ,000 | ,000 | ,000 | ,000 | ,000 | ,000 | ,000 | ,000 | ,000 | ,000 | ,000 | ,000 | ,000 | ,000 | ,000 | ,000 | ,000 | 1,000 |
| par\_30 | ,000 | ,000 | ,000 | ,000 | ,000 | ,000 | ,000 | ,000 | ,000 | ,000 | ,000 | ,000 | ,000 | ,000 | ,000 | ,000 | ,000 | ,000 | ,000 | ,000 | ,000 | ,000 | ,000 | ,000 | ,000 | ,000 | ,000 | ,000 | ,000 | 1,000 |
| par\_31 | -,046 | -,099 | -,016 | -,013 | -,029 | -,002 | -,008 | -,005 | ,000 | ,000 | ,000 | -,043 | -,300 | -,499 | -,592 | -,085 | -,039 | -,008 | -,017 | ,010 | ,004 | ,007 | ,001 | ,003 | ,010 | ,000 | ,000 | ,000 | ,000 | ,000 | 1,000 |
| par\_32 | -,039 | -,091 | ,083 | ,026 | -,033 | -,002 | -,003 | -,005 | ,000 | ,000 | ,000 | -,041 | -,296 | -,495 | -,579 | -,091 | -,025 | ,003 | -,017 | ,004 | ,000 | ,006 | ,000 | ,000 | ,009 | ,000 | ,000 | ,000 | ,000 | ,000 | ,981 | 1,000 |
| par\_33 | -,036 | -,091 | ,025 | ,091 | -,032 | -,001 | -,008 | -,005 | ,000 | ,000 | ,000 | -,042 | -,295 | -,492 | -,577 | -,090 | -,028 | ,000 | -,017 | ,000 | ,001 | ,006 | ,003 | ,001 | ,010 | ,000 | ,000 | ,000 | ,000 | ,000 | ,976 | ,965 | 1,000 |
| par\_34 | ,246 | ,012 | ,004 | ,013 | -,202 | -,178 | -,005 | -,006 | ,000 | ,000 | ,000 | -,067 | -,316 | -,464 | -,514 | -,174 | -,024 | ,030 | -,021 | -,008 | -,010 | ,008 | ,005 | -,006 | ,007 | ,000 | ,000 | ,000 | ,000 | ,000 | ,888 | ,878 | ,874 | 1,000 |
| par\_35 | ,134 | ,014 | -,005 | ,006 | -,005 | -,028 | -,005 | -,006 | ,000 | ,000 | ,000 | -,063 | -,332 | -,486 | -,547 | -,185 | -,016 | ,029 | -,017 | -,012 | -,010 | -,005 | ,006 | -,005 | ,011 | ,000 | ,000 | ,000 | ,000 | ,000 | ,940 | ,924 | ,922 | ,921 | 1,000 |
| par\_36 | ,131 | ,010 | ,003 | ,015 | -,052 | ,023 | -,007 | -,009 | ,000 | ,000 | ,000 | -,071 | -,330 | -,489 | -,546 | -,182 | -,026 | ,028 | -,011 | ,000 | -,010 | -,001 | ,009 | -,005 | ,015 | ,000 | ,000 | ,000 | ,000 | ,000 | ,940 | ,928 | ,928 | ,921 | ,968 | 1,000 |
| par\_37 | ,034 | ,123 | ,000 | ,001 | -,028 | -,005 | -,013 | -,013 | ,000 | ,000 | ,000 | -,064 | -,322 | -,482 | -,564 | -,185 | -,023 | -,010 | -,032 | ,010 | ,006 | ,007 | ,004 | ,002 | ,012 | ,000 | ,000 | ,000 | ,000 | ,000 | ,947 | ,935 | ,930 | ,885 | ,936 | ,936 | 1,000 |
| par\_38 | ,034 | ,119 | ,004 | ,001 | -,027 | -,005 | ,066 | ,016 | ,000 | ,000 | ,000 | -,060 | -,317 | -,476 | -,556 | -,184 | -,022 | ,000 | -,025 | ,009 | -,002 | ,006 | ,004 | ,002 | ,003 | ,000 | ,000 | ,000 | ,000 | ,000 | ,935 | ,926 | ,917 | ,874 | ,924 | ,923 | ,985 | 1,000 |
| par\_39 | ,033 | ,117 | ,001 | ,001 | -,027 | -,006 | ,021 | ,078 | ,000 | ,000 | ,000 | -,060 | -,314 | -,472 | -,552 | -,181 | -,022 | ,000 | -,024 | ,009 | -,003 | ,006 | ,004 | ,004 | ,005 | ,000 | ,000 | ,000 | ,000 | ,000 | ,926 | ,914 | ,910 | ,866 | ,915 | ,913 | ,976 | ,965 | 1,000 |
| par\_40 | ,000 | ,000 | ,000 | ,000 | ,000 | ,000 | ,000 | ,000 | ,447 | -,037 | -,119 | ,000 | ,000 | ,000 | ,000 | ,000 | ,000 | ,000 | ,000 | ,000 | ,000 | ,000 | ,000 | ,000 | ,000 | ,000 | ,000 | ,000 | ,000 | ,000 | ,000 | ,000 | ,000 | ,000 | ,000 | ,000 | ,000 | ,000 | ,000 | 1,000 |
| par\_41 | ,000 | ,000 | ,000 | ,000 | ,000 | ,000 | ,000 | ,000 | ,043 | -,498 | -,119 | ,000 | ,000 | ,000 | ,000 | ,000 | ,000 | ,000 | ,000 | ,000 | ,000 | ,000 | ,000 | ,000 | ,000 | ,000 | ,000 | ,000 | ,000 | ,000 | ,000 | ,000 | ,000 | ,000 | ,000 | ,000 | ,000 | ,000 | ,000 | ,007 | 1,000 |
| par\_42 | ,000 | ,000 | ,000 | ,000 | ,000 | ,000 | ,000 | ,000 | ,447 | -,498 | -,176 | ,000 | ,000 | ,000 | ,000 | ,000 | ,000 | ,000 | ,000 | ,000 | ,000 | ,000 | ,000 | ,000 | ,000 | ,000 | ,000 | ,000 | ,000 | ,000 | ,000 | ,000 | ,000 | ,000 | ,000 | ,000 | ,000 | ,000 | ,000 | ,111 | ,141 | 1,000 |
| par\_43 | ,000 | ,000 | ,000 | ,000 | ,000 | ,000 | ,000 | ,000 | ,000 | ,000 | ,000 | ,000 | ,000 | ,000 | ,000 | ,000 | ,000 | ,000 | ,000 | ,000 | ,000 | ,000 | ,000 | ,000 | ,000 | ,000 | ,000 | ,000 | ,000 | ,000 | ,000 | ,000 | ,000 | ,000 | ,000 | ,000 | ,000 | ,000 | ,000 | ,000 | ,000 | ,000 | 1,000 |
| par\_44 | ,000 | ,000 | ,000 | ,000 | ,000 | ,000 | ,000 | ,000 | ,000 | ,000 | ,000 | ,000 | ,000 | ,000 | ,000 | ,000 | ,000 | ,000 | ,000 | ,000 | ,000 | ,000 | ,000 | ,000 | ,000 | ,000 | ,000 | ,000 | ,000 | ,000 | ,000 | ,000 | ,000 | ,000 | ,000 | ,000 | ,000 | ,000 | ,000 | ,000 | ,000 | ,000 | ,000 | 1,000 |
| par\_45 | -,551 | -,510 | -,118 | -,092 | ,039 | ,051 | ,015 | ,013 | ,000 | ,000 | ,000 | ,077 | ,120 | -,009 | -,117 | ,361 | -,081 | -,175 | -,012 | ,048 | ,063 | ,040 | -,033 | ,040 | -,015 | ,000 | ,000 | ,000 | ,000 | ,000 | ,066 | ,054 | ,055 | -,098 | -,096 | -,093 | -,047 | -,045 | -,045 | ,000 | ,000 | ,000 | ,000 | ,000 | 1,000 |
| par\_46 | ,292 | ,251 | -,352 | -,296 | -,002 | -,016 | -,005 | -,003 | ,000 | ,000 | ,000 | -,063 | -,068 | -,004 | ,055 | -,187 | -,066 | ,026 | ,005 | ,067 | ,000 | -,023 | ,042 | -,008 | ,011 | ,000 | ,000 | ,000 | ,000 | ,000 | -,022 | -,056 | -,052 | ,064 | ,068 | ,064 | ,034 | ,033 | ,033 | ,000 | ,000 | ,000 | ,000 | ,000 | -,378 | 1,000 |
| par\_47 | ,287 | -,050 | ,022 | -,007 | -,713 | -,725 | -,011 | ,001 | ,000 | ,000 | ,000 | ,013 | ,035 | ,012 | -,041 | ,083 | -,007 | -,044 | -,027 | ,025 | ,021 | ,014 | -,013 | ,007 | ,004 | ,000 | ,000 | ,000 | ,000 | ,000 | ,014 | ,016 | ,013 | ,095 | -,048 | -,052 | ,003 | ,002 | ,003 | ,000 | ,000 | ,000 | ,000 | ,000 | ,079 | -,114 | 1,000 |
| par\_48 | ,053 | -,253 | ,009 | ,018 | -,021 | -,012 | -,327 | -,247 | ,000 | ,000 | ,000 | -,017 | -,019 | -,024 | -,011 | ,107 | -,004 | -,006 | ,004 | -,025 | ,044 | -,010 | ,006 | -,012 | ,076 | ,000 | ,000 | ,000 | ,000 | ,000 | ,032 | ,033 | ,034 | ,045 | ,044 | ,045 | -,024 | -,049 | -,046 | ,000 | ,000 | ,000 | ,000 | ,000 | -,109 | ,011 | -,020 | 1,000 |
| par\_49 | ,079 | ,042 | ,625 | ,509 | -,024 | -,016 | ,019 | ,005 | ,000 | ,000 | ,000 | ,035 | -,001 | -,056 | ,070 | -,066 | ,097 | ,130 | ,004 | -,163 | -,057 | -,013 | -,012 | -,030 | -,008 | ,000 | ,000 | ,000 | ,000 | ,000 | -,013 | ,049 | ,040 | ,011 | ,006 | ,008 | -,004 | -,002 | -,003 | ,000 | ,000 | ,000 | ,000 | ,000 | -,114 | -,435 | ,003 | ,032 | 1,000 |
| par\_50 | -,036 | -,025 | -,171 | -,229 | ,011 | ,004 | ,000 | ,002 | ,000 | ,000 | ,000 | -,005 | ,008 | ,014 | -,023 | ,029 | -,021 | -,024 | ,002 | ,046 | ,005 | ,001 | ,152 | ,029 | ,003 | ,000 | ,000 | ,000 | ,000 | ,000 | ,005 | -,012 | -,019 | -,006 | -,004 | -,005 | -,001 | -,001 | ,000 | ,000 | ,000 | ,000 | ,000 | ,000 | ,041 | ,113 | ,003 | -,010 | -,282 | 1,000 |
| par\_51 | -,043 | -,014 | -,425 | -,262 | ,017 | ,008 | -,024 | -,009 | ,000 | ,000 | ,000 | -,026 | -,005 | ,040 | -,043 | ,034 | -,052 | -,128 | -,005 | -,031 | ,153 | ,003 | ,002 | ,034 | ,008 | ,000 | ,000 | ,000 | ,000 | ,000 | ,006 | -,036 | -,022 | -,007 | -,004 | -,006 | ,003 | ,001 | ,002 | ,000 | ,000 | ,000 | ,000 | ,000 | ,071 | ,233 | -,004 | -,022 | -,520 | ,132 | 1,000 |
| par\_52 | -,180 | ,001 | -,001 | -,008 | ,219 | ,227 | ,001 | ,001 | ,000 | ,000 | ,000 | ,014 | ,012 | ,018 | -,005 | -,006 | -,025 | -,010 | ,021 | ,014 | ,004 | -,019 | ,006 | ,003 | ,005 | ,000 | ,000 | ,000 | ,000 | ,000 | -,014 | -,014 | -,014 | -,064 | -,022 | -,020 | -,013 | -,013 | -,013 | ,000 | ,000 | ,000 | ,000 | ,000 | ,024 | -,010 | -,151 | -,014 | -,007 | ,004 | ,005 | 1,000 |
| par\_53 | ,117 | -,022 | ,019 | ,032 | -,322 | ,029 | ,002 | -,001 | ,000 | ,000 | ,000 | -,063 | -,004 | -,050 | -,006 | ,011 | -,082 | ,008 | ,016 | -,063 | -,007 | ,069 | ,010 | -,001 | -,031 | ,000 | ,000 | ,000 | ,000 | ,000 | ,045 | ,047 | ,048 | ,076 | ,013 | ,086 | ,040 | ,040 | ,039 | ,000 | ,000 | ,000 | ,000 | ,000 | -,006 | ,004 | -,010 | ,013 | -,004 | -,003 | ,004 | -,069 | 1,000 |
| par\_54 | ,137 | ,019 | -,016 | -,018 | ,001 | -,347 | ,001 | ,003 | ,000 | ,000 | ,000 | ,045 | -,005 | ,023 | ,013 | -,005 | ,030 | -,001 | -,070 | -,079 | ,001 | ,054 | ,120 | -,005 | -,067 | ,000 | ,000 | ,000 | ,000 | ,000 | -,027 | -,028 | -,029 | ,014 | ,015 | -,058 | -,023 | -,022 | -,022 | ,000 | ,000 | ,000 | ,000 | ,000 | -,026 | ,007 | ,042 | ,003 | ,016 | ,008 | -,005 | -,097 | -,285 | 1,000 |
| par\_55 | -,015 | ,009 | -,019 | -,002 | ,002 | ,004 | -,442 | -,329 | ,000 | ,000 | ,000 | -,037 | -,010 | -,001 | -,006 | ,019 | -,005 | -,106 | -,059 | ,009 | ,191 | ,044 | ,004 | -,003 | ,102 | ,000 | ,000 | ,000 | ,000 | ,000 | ,008 | ,006 | ,008 | ,003 | ,004 | ,004 | ,010 | -,025 | -,020 | ,000 | ,000 | ,000 | ,000 | ,000 | ,006 | -,008 | ,012 | ,277 | -,009 | ,001 | ,020 | -,001 | -,008 | ,004 | 1,000 |
| par\_56 | ,018 | -,021 | ,026 | -,001 | -,004 | -,006 | ,655 | ,520 | ,000 | ,000 | ,000 | ,058 | ,017 | ,002 | ,007 | -,025 | ,007 | ,146 | ,085 | -,008 | -,148 | -,009 | -,008 | ,007 | -,154 | ,000 | ,000 | ,000 | ,000 | ,000 | -,012 | -,009 | -,011 | -,005 | -,007 | -,007 | -,016 | ,036 | ,032 | ,000 | ,000 | ,000 | ,000 | ,000 | -,002 | ,007 | -,015 | -,434 | ,011 | ,003 | -,019 | ,001 | ,014 | -,008 | -,632 | 1,000 |
| par\_57 | -,005 | ,013 | -,008 | ,001 | ,002 | ,003 | -,236 | -,216 | ,000 | ,000 | ,000 | -,022 | -,007 | -,001 | -,002 | ,007 | -,003 | -,054 | -,035 | ,005 | ,056 | ,001 | ,002 | ,018 | ,001 | ,000 | ,000 | ,000 | ,000 | ,000 | ,004 | ,003 | ,004 | ,002 | ,003 | ,003 | ,007 | -,012 | -,013 | ,000 | ,000 | ,000 | ,000 | ,000 | -,003 | ,001 | ,004 | ,154 | -,003 | -,001 | ,005 | ,000 | -,005 | ,004 | ,226 | -,361 | 1,000 |

##### Critical Ratios for Differences between Parameters (Default model)

|  | par\_1 | par\_2 | par\_3 | par\_4 | par\_5 | par\_6 | par\_7 | par\_8 | par\_9 | par\_10 | par\_11 | par\_12 | par\_13 | par\_14 | par\_15 | par\_16 | par\_17 | par\_18 | par\_19 | par\_20 | par\_21 | par\_22 | par\_23 | par\_24 | par\_25 | par\_26 | par\_27 | par\_28 | par\_29 | par\_30 | par\_31 | par\_32 | par\_33 | par\_34 | par\_35 | par\_36 | par\_37 | par\_38 | par\_39 | par\_40 | par\_41 | par\_42 | par\_43 | par\_44 | par\_45 | par\_46 | par\_47 | par\_48 | par\_49 | par\_50 | par\_51 | par\_52 | par\_53 | par\_54 | par\_55 | par\_56 | par\_57 |
| --- | --- | --- | --- | --- | --- | --- | --- | --- | --- | --- | --- | --- | --- | --- | --- | --- | --- | --- | --- | --- | --- | --- | --- | --- | --- | --- | --- | --- | --- | --- | --- | --- | --- | --- | --- | --- | --- | --- | --- | --- | --- | --- | --- | --- | --- | --- | --- | --- | --- | --- | --- | --- | --- | --- | --- | --- | --- |
| par\_1 | ,000 |
| par\_2 | -3,136 | ,000 |
| par\_3 | -3,750 | -3,454 | ,000 |
| par\_4 | -4,095 | -5,003 | -8,589 | ,000 |
| par\_5 | -4,015 | -5,004 | -6,251 | -,381 | ,000 |
| par\_6 | -4,245 | -5,788 | -11,767 | -8,503 | -5,697 | ,000 |
| par\_7 | -3,601 | -2,790 | 2,318 | 10,192 | 8,872 | 14,915 | ,000 |
| par\_8 | -4,142 | -5,210 | -8,758 | -2,312 | -,985 | 7,757 | -13,263 | ,000 |
| par\_9 | 5,255 | 5,256 | 5,256 | 5,256 | 5,256 | 5,257 | 5,256 | 5,256 | ,000 |
| par\_10 | -6,211 | -5,974 | -5,907 | -5,876 | -5,875 | -5,860 | -5,919 | -5,872 | -5,274 | ,000 |
| par\_11 | -1,660 | -1,486 | -1,439 | -1,418 | -1,417 | -1,407 | -1,447 | -1,415 | -5,253 | 2,595 | ,000 |
| par\_12 | -4,158 | -4,998 | -4,841 | -1,343 | -1,114 | ,740 | -6,370 | -,746 | -5,257 | 5,866 | 1,411 | ,000 |
| par\_13 | -4,310 | -5,963 | -12,808 | -10,672 | -5,697 | -3,360 | -16,045 | -10,881 | -5,257 | 5,856 | 1,404 | -1,264 | ,000 |
| par\_14 | -4,334 | -6,074 | -13,501 | -12,805 | -6,353 | -6,341 | -16,752 | -14,469 | -5,257 | 5,855 | 1,403 | -1,473 | -1,971 | ,000 |
| par\_15 | -4,333 | -6,072 | -13,499 | -12,796 | -6,332 | -6,277 | -16,738 | -14,430 | -5,257 | 5,855 | 1,403 | -1,466 | -1,914 | ,786 | ,000 |
| par\_16 | -3,456 | -2,183 | ,778 | 3,394 | 3,451 | 4,835 | -,211 | 3,849 | -5,256 | 5,916 | 1,445 | 3,495 | 5,135 | 5,329 | 5,315 | ,000 |
| par\_17 | -4,173 | -2,926 | -1,735 | -1,136 | -1,107 | -,822 | -1,956 | -1,041 | -5,257 | 5,810 | 1,377 | -,925 | -,746 | -,715 | -,716 | -1,832 | ,000 |
| par\_18 | -4,434 | -6,117 | -8,762 | -4,643 | -3,935 | -1,982 | -10,635 | -3,935 | -5,257 | 5,848 | 1,399 | -1,860 | -1,327 | -1,072 | -1,082 | -5,109 | ,591 | ,000 |
| par\_19 | -3,880 | -2,472 | -1,311 | -,691 | -,663 | -,363 | -1,572 | -,596 | -5,257 | 5,836 | 1,394 | -,475 | -,282 | -,250 | -,251 | -1,468 | ,354 | -,120 | ,000 |
| par\_20 | -4,374 | -6,238 | -11,723 | -7,873 | -5,860 | -3,554 | -14,625 | -7,243 | -5,257 | 5,850 | 1,400 | -1,917 | -2,132 | -1,642 | -1,662 | -5,583 | ,625 | ,292 | ,159 | ,000 |
| par\_21 | -4,273 | -5,796 | -10,070 | -5,441 | -3,799 | -,298 | -12,404 | -4,184 | -5,257 | 5,859 | 1,407 | -,800 | 1,170 | 1,846 | 1,822 | -4,822 | ,805 | 1,508 | ,346 | 2,509 | ,000 |
| par\_22 | -4,309 | -5,906 | -10,115 | -5,589 | -4,000 | -1,101 | -12,622 | -4,516 | -5,257 | 5,857 | 1,405 | -1,114 | ,120 | ,640 | ,621 | -4,997 | ,751 | 1,194 | ,287 | 1,639 | -,676 | ,000 |
| par\_23 | -4,326 | -6,037 | -13,246 | -12,175 | -6,081 | -5,129 | -16,483 | -13,242 | -5,257 | 5,856 | 1,404 | -1,372 | -,962 | 2,144 | 1,995 | -5,255 | ,729 | 1,194 | ,265 | 1,897 | -1,544 | -,392 | ,000 |
| par\_24 | -4,330 | -6,057 | -13,323 | -12,260 | -6,213 | -5,485 | -16,590 | -13,592 | -5,257 | 5,855 | 1,404 | -1,431 | -1,448 | ,749 | ,605 | -5,296 | ,721 | 1,105 | ,256 | 1,733 | -1,729 | -,540 | -,857 | ,000 |
| par\_25 | -4,335 | -6,079 | -13,493 | -12,704 | -6,368 | -6,224 | -16,606 | -13,932 | -5,257 | 5,855 | 1,403 | -1,485 | -1,986 | -,381 | -,590 | -5,332 | ,713 | 1,051 | ,247 | 1,595 | -1,882 | -,672 | -1,943 | -,888 | ,000 |
| par\_26 | 97,435 | 97,698 | 97,744 | 97,761 | 97,762 | 97,771 | 97,737 | 97,764 | -4,644 | 87,796 | 75,909 | 97,766 | 97,773 | 97,774 | 97,774 | 97,736 | 97,753 | 97,777 | 97,743 | 97,776 | 97,771 | 97,772 | 97,773 | 97,774 | 97,774 | ,000 |
| par\_27 | -4,271 | -5,580 | -6,493 | -2,639 | -2,235 | -,146 | -8,185 | -1,940 | -5,257 | 5,859 | 1,406 | -,670 | ,482 | ,742 | ,733 | -4,343 | ,798 | 1,277 | ,341 | 1,333 | -,016 | ,381 | ,620 | ,693 | ,759 | -97,741 | ,000 |
| par\_28 | 86,159 | 86,637 | 86,711 | 86,739 | 86,740 | 86,753 | 86,700 | 86,743 | -4,908 | 68,699 | 53,951 | 86,746 | 86,756 | 86,758 | 86,758 | 86,699 | 86,700 | 86,762 | 86,688 | 86,762 | 86,754 | 86,756 | 86,757 | 86,758 | 86,758 | -42,396 | 86,574 | ,000 |
| par\_29 | 18,895 | 32,433 | 34,607 | 35,075 | 35,069 | 35,307 | 34,434 | 35,146 | -5,248 | 8,342 | 3,114 | 35,033 | 35,363 | 35,388 | 35,387 | 34,071 | 29,067 | 35,355 | 29,323 | 35,424 | 35,299 | 35,327 | 35,377 | 35,383 | 35,389 | -96,261 | 35,190 | -84,358 | ,000 |
| par\_30 | -4,308 | -5,650 | -6,104 | -2,712 | -2,385 | -,618 | -7,578 | -2,120 | -5,257 | 5,856 | 1,404 | -,967 | -,093 | ,119 | ,112 | -4,415 | ,725 | ,760 | ,265 | ,670 | -,486 | -,134 | ,019 | ,080 | ,134 | -97,772 | -,383 | -86,755 | -35,184 | ,000 |
| par\_31 | -,113 | 4,040 | 5,636 | 6,193 | 6,203 | 6,484 | 5,421 | 6,281 | -5,255 | 6,213 | 1,653 | 6,291 | 6,526 | 6,580 | 6,580 | 5,281 | 5,228 | 6,653 | 5,085 | 6,657 | 6,493 | 6,541 | 6,569 | 6,578 | 6,585 | -97,514 | 6,461 | -86,324 | -23,725 | 6,510 | ,000 |
| par\_32 | -1,501 | 3,757 | 8,028 | 9,297 | 9,240 | 9,970 | 7,409 | 9,488 | -5,256 | 6,095 | 1,569 | 9,175 | 10,038 | 10,197 | 10,198 | 6,615 | 4,879 | 10,175 | 4,669 | 10,333 | 9,954 | 10,051 | 10,177 | 10,195 | 10,214 | -97,626 | 9,693 | -86,523 | -30,321 | 9,709 | -3,817 | ,000 |
| par\_33 | -3,372 | -1,723 | 2,799 | 6,056 | 5,840 | 7,639 | 1,614 | 6,478 | -5,256 | 5,936 | 1,459 | 5,530 | 7,856 | 8,203 | 8,204 | 1,243 | 2,241 | 7,617 | 1,875 | 8,368 | 7,507 | 7,682 | 8,146 | 8,190 | 8,236 | -97,726 | 6,579 | -86,682 | -33,916 | 6,507 | -6,192 | -11,161 | ,000 |
| par\_34 | 4,761 | 6,104 | 6,501 | 6,666 | 6,649 | 6,745 | 6,434 | 6,690 | -5,252 | 6,995 | 2,246 | 6,697 | 6,763 | 6,780 | 6,780 | 6,369 | 6,685 | 6,817 | 6,589 | 6,803 | 6,754 | 6,770 | 6,777 | 6,779 | 6,781 | -96,511 | 6,752 | -84,489 | -6,277 | 6,770 | 6,437 | 6,154 | 6,650 | ,000 |
| par\_35 | -,719 | 5,678 | 9,234 | 10,379 | 10,372 | 10,943 | 8,806 | 10,548 | -5,255 | 6,168 | 1,619 | 10,239 | 10,989 | 11,141 | 11,142 | 7,826 | 5,968 | 11,201 | 5,789 | 11,244 | 10,932 | 11,013 | 11,125 | 11,139 | 11,156 | -97,580 | 10,738 | -86,449 | -28,720 | 10,759 | -1,676 | 6,635 | 11,974 | -5,897 | ,000 |
| par\_36 | -3,658 | -2,781 | 1,149 | 5,116 | 4,905 | 7,269 | -,277 | 5,758 | -5,256 | 5,916 | 1,445 | 4,532 | 7,513 | 7,981 | 7,983 | ,013 | 1,886 | 7,116 | 1,496 | 8,103 | 7,006 | 7,206 | 7,907 | 7,959 | 8,022 | -97,738 | 5,782 | -86,701 | -34,316 | 5,689 | -6,286 | -10,730 | -4,927 | -6,704 | -12,128 | ,000 |
| par\_37 | -,208 | 4,232 | 5,560 | 6,122 | 6,130 | 6,414 | 5,334 | 6,207 | -5,255 | 6,204 | 1,647 | 6,201 | 6,456 | 6,512 | 6,512 | 5,097 | 5,189 | 6,585 | 4,969 | 6,590 | 6,425 | 6,472 | 6,502 | 6,510 | 6,518 | -97,521 | 6,392 | -86,336 | -23,964 | 6,441 | -,509 | 3,432 | 6,030 | -6,463 | 1,404 | 6,197 | ,000 |
| par\_38 | -1,336 | 4,140 | 7,068 | 8,162 | 8,151 | 8,724 | 6,702 | 8,337 | -5,256 | 6,110 | 1,579 | 8,139 | 8,785 | 8,911 | 8,912 | 5,941 | 4,945 | 8,954 | 4,718 | 9,040 | 8,722 | 8,808 | 8,893 | 8,908 | 8,923 | -97,613 | 8,567 | -86,500 | -29,407 | 8,610 | -3,594 | 1,369 | 8,727 | -6,219 | -5,246 | 8,916 | -3,729 | ,000 |
| par\_39 | -3,514 | -2,410 | 2,315 | 6,424 | 6,181 | 8,616 | ,884 | 7,197 | -5,256 | 5,926 | 1,452 | 5,476 | 8,859 | 9,367 | 9,369 | ,672 | 2,076 | 8,096 | 1,693 | 9,438 | 8,323 | 8,500 | 9,293 | 9,350 | 9,409 | -97,732 | 6,829 | -86,692 | -34,183 | 6,651 | -6,058 | -10,010 | -2,242 | -6,629 | -11,320 | 3,079 | -6,025 | -8,601 | ,000 |
| par\_40 | 11,768 | 11,768 | 11,769 | 11,769 | 11,769 | 11,769 | 11,768 | 11,769 | 10,336 | 11,778 | 11,769 | 11,769 | 11,769 | 11,769 | 11,769 | 11,768 | 11,769 | 11,769 | 11,769 | 11,769 | 11,769 | 11,769 | 11,769 | 11,769 | 11,769 | 11,476 | 11,769 | 11,602 | 11,764 | 11,769 | 11,768 | 11,768 | 11,768 | 11,766 | 11,768 | 11,768 | 11,768 | 11,768 | 11,768 | ,000 |
| par\_41 | -3,343 | -1,654 | 4,128 | 8,605 | 8,272 | 10,949 | 2,640 | 9,358 | -5,256 | 5,919 | 1,461 | 7,120 | 11,495 | 11,780 | 11,772 | 1,778 | 2,359 | 9,842 | 1,991 | 11,624 | 10,513 | 10,613 | 11,665 | 11,723 | 11,787 | -97,724 | 8,502 | -86,680 | -33,999 | 8,172 | -5,005 | -6,303 | ,373 | -6,314 | -7,871 | 2,274 | -4,914 | -5,770 | 1,353 | -11,768 | ,000 |
| par\_42 | 11,767 | 11,768 | 11,768 | 11,769 | 11,769 | 11,769 | 11,768 | 11,769 | 4,950 | 11,769 | 11,764 | 11,769 | 11,769 | 11,769 | 11,769 | 11,768 | 11,769 | 11,769 | 11,769 | 11,769 | 11,769 | 11,769 | 11,769 | 11,769 | 11,769 | 11,058 | 11,769 | 11,364 | 11,758 | 11,769 | 11,767 | 11,768 | 11,768 | 11,763 | 11,767 | 11,768 | 11,767 | 11,768 | 11,768 | -6,661 | 11,768 | ,000 |
| par\_43 | -2,906 | ,109 | 6,410 | 9,602 | 9,435 | 11,214 | 5,312 | 10,112 | -5,256 | 5,978 | 1,487 | 8,692 | 11,593 | 11,777 | 11,771 | 3,962 | 3,049 | 10,795 | 2,721 | 11,816 | 11,008 | 11,125 | 11,699 | 11,740 | 11,783 | -97,702 | 9,764 | -86,645 | -33,289 | 9,543 | -4,302 | -4,587 | 2,923 | -6,108 | -6,383 | 4,739 | -4,200 | -4,375 | 3,998 | -11,768 | 2,940 | -11,768 | ,000 |
| par\_44 | 11,326 | 11,640 | 11,715 | 11,746 | 11,748 | 11,763 | 11,702 | 11,751 | -5,216 | 12,370 | 7,623 | 11,757 | 11,767 | 11,769 | 11,769 | 11,704 | 11,790 | 11,775 | 11,766 | 11,773 | 11,764 | 11,767 | 11,768 | 11,768 | 11,769 | -80,098 | 11,764 | -58,363 | 9,127 | 11,767 | 11,366 | 11,513 | 11,683 | 10,263 | 11,434 | 11,704 | 11,376 | 11,495 | 11,694 | -11,749 | 11,679 | -11,722 | 11,639 | ,000 |
| par\_45 | -3,305 | -2,006 | ,411 | 2,331 | 2,425 | 3,390 | -,338 | 2,662 | -5,256 | 5,913 | 1,443 | 2,815 | 3,653 | 3,736 | 3,731 | -,181 | 1,735 | 3,669 | 1,380 | 3,991 | 3,427 | 3,572 | 3,685 | 3,720 | 3,741 | -97,736 | 3,204 | -86,697 | -33,745 | 3,327 | -5,422 | -6,620 | -1,249 | -6,390 | -7,643 | -,169 | -5,177 | -5,943 | -,727 | -11,769 | -1,569 | -11,768 | -3,427 | -11,706 | ,000 |
| par\_46 | -2,889 | ,645 | 3,602 | 5,107 | 5,242 | 5,948 | 3,261 | 5,439 | -5,256 | 5,991 | 1,497 | 5,298 | 6,115 | 6,204 | 6,202 | 2,831 | 3,039 | 6,312 | 2,803 | 6,408 | 5,953 | 6,040 | 6,174 | 6,189 | 6,210 | -97,687 | 5,777 | -86,619 | -31,929 | 5,846 | -3,805 | -3,141 | 2,275 | -6,045 | -5,139 | 3,297 | -3,772 | -3,358 | 2,843 | -11,768 | 2,216 | -11,768 | ,579 | -11,622 | 2,493 | ,000 |
| par\_47 | 2,162 | 2,852 | 3,078 | 3,170 | 3,153 | 3,213 | 3,039 | 3,185 | -5,253 | 6,569 | 2,073 | 3,202 | 3,232 | 3,236 | 3,236 | 3,055 | 3,304 | 3,252 | 3,225 | 3,250 | 3,222 | 3,230 | 3,234 | 3,235 | 3,236 | -95,528 | 3,222 | -82,421 | -4,358 | 3,233 | 2,083 | 2,490 | 2,987 | -,571 | 2,249 | 3,042 | 2,108 | 2,437 | 3,016 | -11,767 | 2,972 | -11,764 | 2,857 | -10,100 | 3,064 | 2,787 | ,000 |
| par\_48 | -1,893 | 2,396 | 5,903 | 7,099 | 7,090 | 7,704 | 5,200 | 7,199 | -5,256 | 6,062 | 1,546 | 7,178 | 7,851 | 7,919 | 7,917 | 5,335 | 4,246 | 7,952 | 4,004 | 8,024 | 7,738 | 7,784 | 7,891 | 7,906 | 7,929 | -97,643 | 7,542 | -86,548 | -30,401 | 7,592 | -2,601 | -,948 | 4,588 | -5,635 | -2,826 | 5,427 | -2,426 | -1,213 | 4,947 | -11,768 | 4,503 | -11,768 | 3,022 | -11,546 | 4,680 | 2,091 | -2,582 | ,000 |
| par\_49 | -4,050 | -4,197 | -2,656 | ,348 | ,425 | 1,664 | -3,116 | ,718 | -5,256 | 5,880 | 1,421 | 1,028 | 1,995 | 2,132 | 2,128 | -2,111 | 1,203 | 2,520 | ,750 | 2,360 | 1,680 | 1,900 | 2,068 | 2,104 | 2,140 | -97,757 | 1,550 | -86,731 | -34,548 | 1,747 | -5,979 | -8,336 | -3,687 | -6,635 | -9,480 | -2,573 | -5,914 | -7,431 | -3,266 | -11,769 | -4,333 | -11,768 | -6,180 | -11,741 | -1,604 | -3,819 | -3,157 | -6,423 | ,000 |
| par\_50 | -4,249 | -5,696 | -11,078 | -7,225 | -3,944 | 1,670 | -14,592 | -6,984 | -5,257 | 5,862 | 1,408 | -,497 | 5,472 | 10,518 | 10,433 | -4,686 | ,858 | 2,300 | ,401 | 4,293 | 1,007 | 1,718 | 9,053 | 8,934 | 9,929 | -97,769 | ,444 | -86,751 | -35,282 | ,868 | -6,451 | -9,892 | -7,453 | -6,741 | -10,888 | -7,024 | -6,381 | -8,662 | -8,399 | -11,769 | -10,723 | -11,769 | -11,051 | -11,761 | -3,271 | -5,891 | -3,214 | -7,636 | -1,488 | ,000 |
| par\_51 | -3,985 | -4,505 | -3,638 | 2,010 | 2,114 | 7,041 | -6,680 | 3,657 | -5,256 | 5,885 | 1,424 | 2,237 | 8,192 | 8,908 | 8,882 | -2,663 | 1,286 | 5,063 | ,859 | 7,860 | 6,463 | 6,146 | 8,636 | 8,790 | 8,911 | -97,757 | 3,582 | -86,731 | -34,929 | 3,551 | -6,042 | -8,823 | -4,905 | -6,619 | -10,028 | -3,796 | -5,965 | -7,838 | -5,030 | -11,769 | -6,994 | -11,768 | -8,512 | -11,738 | -1,827 | -4,981 | -3,145 | -6,721 | ,315 | 6,744 | ,000 |
| par\_52 | 9,813 | 10,494 | 10,600 | 10,644 | 10,657 | 10,671 | 10,582 | 10,651 | -5,231 | 10,843 | 5,769 | 10,661 | 10,674 | 10,676 | 10,676 | 10,583 | 10,680 | 10,684 | 10,683 | 10,683 | 10,669 | 10,672 | 10,675 | 10,676 | 10,676 | -87,281 | 10,669 | -68,715 | 6,946 | 10,674 | 10,090 | 10,309 | 10,553 | 8,373 | 10,193 | 10,582 | 10,105 | 10,282 | 10,569 | -11,756 | 10,549 | -11,738 | 10,493 | -3,428 | 10,593 | 10,465 | 7,782 | 10,355 | 10,636 | 10,665 | 10,632 | ,000 |
| par\_53 | -3,912 | -3,927 | -1,860 | 2,890 | 2,511 | 5,679 | -3,603 | 3,713 | -5,256 | 5,894 | 1,430 | 2,826 | 6,324 | 6,652 | 6,645 | -1,752 | 1,449 | 5,477 | 1,044 | 6,579 | 5,354 | 5,766 | 6,520 | 6,590 | 6,656 | -97,751 | 4,035 | -86,722 | -34,721 | 4,029 | -5,882 | -8,411 | -3,765 | -6,584 | -9,602 | -2,459 | -5,800 | -7,480 | -3,465 | -11,769 | -5,019 | -11,768 | -7,036 | -11,728 | -1,175 | -4,305 | -3,116 | -6,310 | 1,021 | 5,359 | 1,363 | -10,612 | ,000 |
| par\_54 | -4,303 | -5,929 | -12,651 | -10,700 | -5,385 | -2,377 | -15,897 | -11,254 | -5,257 | 5,858 | 1,405 | -1,089 | 1,467 | 6,787 | 6,675 | -5,066 | ,772 | 1,563 | ,309 | 2,661 | -,694 | ,309 | 4,196 | 4,505 | 5,733 | -97,772 | -,271 | -86,755 | -35,346 | ,272 | -6,529 | -10,080 | -7,914 | -6,765 | -11,050 | -7,596 | -6,461 | -8,815 | -8,993 | -11,769 | -11,362 | -11,769 | -11,490 | -11,766 | -3,547 | -6,073 | -3,227 | -7,809 | -1,888 | -5,456 | -7,965 | -10,671 | -6,019 | ,000 |
| par\_55 | -3,841 | -3,840 | -1,513 | 4,061 | 3,843 | 7,478 | -2,937 | 4,781 | -5,256 | 5,897 | 1,432 | 3,425 | 8,273 | 8,722 | 8,710 | -1,537 | 1,531 | 6,076 | 1,109 | 8,463 | 7,483 | 7,184 | 8,549 | 8,632 | 8,782 | -97,749 | 4,806 | -86,719 | -34,709 | 4,682 | -5,807 | -8,262 | -3,542 | -6,551 | -9,524 | -2,126 | -5,728 | -7,320 | -3,202 | -11,769 | -4,961 | -11,768 | -7,012 | -11,724 | -,992 | -4,185 | -3,106 | -6,542 | 1,320 | 7,138 | 2,159 | -10,613 | ,451 | 8,096 | ,000 |
| par\_56 | -4,211 | -4,814 | -3,519 | -1,103 | -,962 | ,234 | -5,698 | -,753 | -5,257 | 5,863 | 1,409 | -,208 | ,564 | ,697 | ,693 | -3,174 | ,855 | 1,171 | ,416 | 1,040 | ,287 | ,514 | ,634 | ,672 | ,704 | -97,767 | ,276 | -86,746 | -34,788 | ,528 | -6,280 | -8,789 | -4,700 | -6,720 | -9,995 | -3,765 | -6,202 | -8,089 | -4,555 | -11,769 | -5,569 | -11,769 | -7,269 | -11,759 | -2,558 | -5,155 | -3,207 | -6,029 | -1,023 | ,078 | -1,734 | -10,662 | -2,330 | ,452 | -2,214 | ,000 |
| par\_57 | -4,275 | -5,816 | -12,070 | -9,401 | -4,682 | -,377 | -14,878 | -8,691 | -5,257 | 5,860 | 1,407 | -,791 | 3,821 | 10,803 | 10,727 | -4,876 | ,815 | 1,930 | ,355 | 3,505 | ,168 | 1,023 | 7,510 | 7,962 | 9,542 | -97,771 | ,087 | -86,753 | -35,315 | ,571 | -6,491 | -9,992 | -7,699 | -6,753 | -10,970 | -7,332 | -6,422 | -8,739 | -8,703 | -11,769 | -11,054 | -11,769 | -11,276 | -11,763 | -3,408 | -5,972 | -3,220 | -7,745 | -1,700 | -2,641 | -7,304 | -10,668 | -5,758 | 3,500 | -7,803 | -,262 | ,000 |

##### Model Fit Summary

##### CMIN

| Model | NPAR | CMIN | DF | P | CMIN/DF |
| --- | --- | --- | --- | --- | --- |
| Default model | 57 | 97,316 | 62 | ,003 | 1,570 |
| Saturated model | 119 | ,000 | 0 |
| Independence model | 28 | 1337,713 | 91 | ,000 | 14,700 |

##### Baseline Comparisons

| Model | NFI Delta1 | RFI rho1 | IFI Delta2 | TLI rho2 | CFI |
| --- | --- | --- | --- | --- | --- |
| Default model | ,927 | ,893 | ,972 | ,958 | ,972 |
| Saturated model | 1,000 |  | 1,000 |  | 1,000 |
| Independence model | ,000 | ,000 | ,000 | ,000 | ,000 |

##### Parsimony-Adjusted Measures

| Model | PRATIO | PNFI | PCFI |
| --- | --- | --- | --- |
| Default model | ,681 | ,632 | ,662 |
| Saturated model | ,000 | ,000 | ,000 |
| Independence model | 1,000 | ,000 | ,000 |

##### NCP

| Model | NCP | LO 90 | HI 90 |
| --- | --- | --- | --- |
| Default model | 35,316 | 12,417 | 66,146 |
| Saturated model | ,000 | ,000 | ,000 |
| Independence model | 1246,713 | 1132,122 | 1368,714 |

##### FMIN

| Model | FMIN | F0 | LO 90 | HI 90 |
| --- | --- | --- | --- | --- |
| Default model | ,351 | ,127 | ,045 | ,239 |
| Saturated model | ,000 | ,000 | ,000 | ,000 |
| Independence model | 4,829 | 4,501 | 4,087 | 4,941 |

##### RMSEA

| Model | RMSEA | LO 90 | HI 90 | PCLOSE |
| --- | --- | --- | --- | --- |
| Default model | ,045 | ,027 | ,062 | ,656 |
| Independence model | ,222 | ,212 | ,233 | ,000 |

##### AIC

| Model | AIC | BCC | BIC | CAIC |
| --- | --- | --- | --- | --- |
| Default model | 211,316 | 217,842 |
| Saturated model | 238,000 | 251,626 |
| Independence model | 1393,713 | 1396,919 |

##### ECVI

| Model | ECVI | LO 90 | HI 90 | MECVI |
| --- | --- | --- | --- | --- |
| Default model | ,763 | ,680 | ,874 | ,786 |
| Saturated model | ,859 | ,859 | ,859 | ,908 |
| Independence model | 5,031 | 4,618 | 5,472 | 5,043 |

##### HOELTER

| Model | HOELTER .05 | HOELTER .01 |
| --- | --- | --- |
| Default model | 232 | 259 |
| Independence model | 24 | 26 |

##### Execution time summary

|  |  |
| --- | --- |
| Minimization: | ,013 |
| Miscellaneous: | 1,388 |
| Bootstrap: | ,000 |
| Total: | 1,401 |
