# Supplementary material for: The Contribution of Executive Functions in Predicting Mathematical Creativity in Typical Elementary School Classes: A Twofold Role for Updating
Source: J Intell. 2020 Jun 2;8(2):26. doi: 10.3390/jintelligence8020026 (PMC7713010; doi:10.3390/jintelligence8020026)
Supplement: Supplementary file 1 [file jintelligence-08-00026-s001.zip › mediation model without missings 01052020.AmosOutput]

mediation model without missings or intelligence 01052020.amw


#### \\Client\D$\Corona Backup 15032020\Selfpublished Articles\Creamath Paper\Journal of Intelligence\Revision\mediation model without missings or intelligence 01052020.amw

##### Analysis Summary

##### Date and Time

Date: vrijdag 1 mei 2020

Time: 10:26:06

##### Title

mediation model without missings or intelligence 01052020: vrijdag 1 mei 2020 10:26

##### Notes

Bootstrap confidence intervals are not reported because you specified a bootfactor value greater than 1.

##### Groups

##### Group number 1 (Group number 1)

##### Notes for Group (Group number 1)

The model is recursive.

Sample size = 278

##### Variable Summary (Group number 1)

##### Your model contains the following variables (Group number 1)

Observed, endogenous variables

URC\_Flu\_1

URC\_Flex\_1

URC\_Org\_1

URC\_Flu\_3

URC\_Flex\_3

URC\_Org\_3

URC\_Flu\_4

URC\_Flex\_4

URC\_Org\_4

MeanRTShift

UpdatingCombined

InhibitionIncongruentRT

MathKnowledge\_Cito

Creativity\_TCTDP

Unobserved, endogenous variables

Task\_1

Math\_DT

Task\_3

Task\_4

Unobserved, exogenous variables

e1

e2

e3

e5

e4

e6

e8

e7

e9

e10

e11

e13

e12

e14

e15

e16

e17

e18

##### Variable counts (Group number 1)

|  |  |
| --- | --- |
| Number of variables in your model: | 36 |
| Number of observed variables: | 14 |
| Number of unobserved variables: | 22 |
| Number of exogenous variables: | 18 |
| Number of endogenous variables: | 18 |

##### Parameter Summary (Group number 1)

|  | Weights | Covariances | Variances | Means | Intercepts | Total |
| --- | --- | --- | --- | --- | --- | --- |
| Fixed | 22 | 0 | 0 | 18 | 4 | 44 |
| Labeled | 0 | 0 | 0 | 0 | 0 | 0 |
| Unlabeled | 15 | 12 | 18 | 0 | 14 | 59 |
| Total | 37 | 12 | 18 | 18 | 18 | 103 |

##### Assessment of normality (Group number 1)

| Variable | min | max | skew | c.r. | kurtosis | c.r. |
| --- | --- | --- | --- | --- | --- | --- |
| UpdatingCombined | -3,198 | 1,633 | -,749 | -5,095 | ,698 | 2,375 |
| Creativity\_TCTDP | 4,000 | 47,000 | ,629 | 4,282 | -,451 | -1,535 |
| MathKnowledge\_Cito | -6,287 | 3,154 | -1,196 | -8,144 | 4,673 | 15,906 |
| InhibitionIncongruentRT | 493,150 | 1315,200 | 1,111 | 7,560 | 1,603 | 5,457 |
| MeanRTShift | 492,640 | 1973,950 | -,472 | -3,215 | ,735 | 2,500 |
| URC\_Org\_4 | ,000 | 1,000 | ,178 | 1,210 | -,819 | -2,787 |
| URC\_Flex\_4 | ,000 | 5,000 | ,108 | ,738 | -,399 | -1,359 |
| URC\_Flu\_4 | ,000 | 9,000 | 1,076 | 7,323 | 2,096 | 7,134 |
| URC\_Org\_3 | ,000 | 1,000 | ,642 | 4,373 | ,309 | 1,051 |
| URC\_Flex\_3 | ,000 | 4,000 | -,549 | -3,734 | ,307 | 1,044 |
| URC\_Flu\_3 | ,000 | 48,000 | 2,484 | 16,911 | 7,228 | 24,602 |
| URC\_Org\_1 | ,000 | 1,000 | ,106 | ,722 | -1,465 | -4,986 |
| URC\_Flex\_1 | ,000 | 3,000 | -,144 | -,980 | -,324 | -1,101 |
| URC\_Flu\_1 | ,000 | 8,000 | ,859 | 5,849 | 1,002 | 3,412 |
| Multivariate |  |  |  |  | 28,506 | 11,228 |

##### Observations farthest from the centroid (Mahalanobis distance) (Group number 1)

| Observation number | Mahalanobis d-squared | p1 | p2 |
| --- | --- | --- | --- |
| 165 | 57,105 | ,000 | ,000 |
| 35 | 49,805 | ,000 | ,000 |
| 119 | 42,031 | ,000 | ,000 |
| 218 | 40,284 | ,000 | ,000 |
| 251 | 37,985 | ,001 | ,000 |
| 198 | 33,125 | ,003 | ,000 |
| 255 | 32,496 | ,003 | ,000 |
| 123 | 32,222 | ,004 | ,000 |
| 168 | 30,729 | ,006 | ,000 |
| 24 | 30,201 | ,007 | ,000 |
| 97 | 28,954 | ,011 | ,000 |
| 48 | 28,898 | ,011 | ,000 |
| 104 | 28,868 | ,011 | ,000 |
| 224 | 28,679 | ,012 | ,000 |
| 59 | 28,570 | ,012 | ,000 |
| 245 | 27,853 | ,015 | ,000 |
| 137 | 27,621 | ,016 | ,000 |
| 90 | 27,608 | ,016 | ,000 |
| 253 | 26,701 | ,021 | ,000 |
| 103 | 25,965 | ,026 | ,000 |
| 151 | 25,284 | ,032 | ,000 |
| 254 | 25,245 | ,032 | ,000 |
| 98 | 24,310 | ,042 | ,002 |
| 102 | 24,241 | ,043 | ,001 |
| 157 | 24,156 | ,044 | ,001 |
| 54 | 23,989 | ,046 | ,001 |
| 22 | 23,183 | ,057 | ,006 |
| 86 | 23,117 | ,058 | ,004 |
| 163 | 22,565 | ,068 | ,014 |
| 14 | 22,169 | ,075 | ,030 |
| 188 | 22,121 | ,076 | ,022 |
| 150 | 21,845 | ,082 | ,033 |
| 28 | 21,690 | ,085 | ,034 |
| 159 | 21,684 | ,085 | ,022 |
| 122 | 21,440 | ,091 | ,031 |
| 152 | 21,428 | ,091 | ,021 |
| 261 | 21,420 | ,091 | ,014 |
| 31 | 21,235 | ,096 | ,017 |
| 67 | 20,949 | ,103 | ,030 |
| 190 | 20,582 | ,113 | ,065 |
| 57 | 20,547 | ,114 | ,051 |
| 175 | 20,298 | ,121 | ,078 |
| 121 | 20,200 | ,124 | ,075 |
| 259 | 19,821 | ,136 | ,158 |
| 234 | 19,791 | ,137 | ,131 |
| 5 | 19,600 | ,143 | ,165 |
| 195 | 19,550 | ,145 | ,146 |
| 239 | 19,112 | ,161 | ,317 |
| 161 | 19,091 | ,161 | ,274 |
| 91 | 18,772 | ,174 | ,420 |
| 138 | 18,699 | ,177 | ,409 |
| 176 | 18,593 | ,181 | ,422 |
| 213 | 18,592 | ,181 | ,364 |
| 162 | 18,311 | ,193 | ,503 |
| 72 | 18,297 | ,194 | ,453 |
| 211 | 18,218 | ,197 | ,451 |
| 249 | 18,081 | ,203 | ,492 |
| 108 | 17,731 | ,219 | ,688 |
| 228 | 17,492 | ,231 | ,790 |
| 182 | 17,430 | ,234 | ,783 |
| 149 | 17,246 | ,243 | ,841 |
| 133 | 17,242 | ,244 | ,806 |
| 3 | 17,138 | ,249 | ,823 |
| 180 | 16,991 | ,257 | ,860 |
| 203 | 16,984 | ,257 | ,830 |
| 260 | 16,857 | ,264 | ,858 |
| 247 | 16,746 | ,270 | ,877 |
| 270 | 16,638 | ,276 | ,893 |
| 52 | 16,637 | ,276 | ,866 |
| 240 | 16,635 | ,276 | ,835 |
| 41 | 16,526 | ,282 | ,856 |
| 174 | 16,239 | ,299 | ,938 |
| 142 | 16,233 | ,299 | ,921 |
| 126 | 16,197 | ,301 | ,912 |
| 276 | 16,182 | ,302 | ,895 |
| 264 | 16,177 | ,303 | ,871 |
| 256 | 15,876 | ,321 | ,951 |
| 147 | 15,824 | ,324 | ,949 |
| 73 | 15,768 | ,328 | ,948 |
| 248 | 15,681 | ,333 | ,954 |
| 272 | 15,619 | ,337 | ,955 |
| 51 | 15,560 | ,341 | ,955 |
| 127 | 15,556 | ,341 | ,942 |
| 236 | 15,527 | ,343 | ,935 |
| 112 | 15,493 | ,345 | ,928 |
| 85 | 15,422 | ,350 | ,932 |
| 21 | 15,279 | ,359 | ,954 |
| 125 | 15,155 | ,368 | ,967 |
| 47 | 15,133 | ,369 | ,961 |
| 235 | 15,096 | ,372 | ,958 |
| 179 | 15,044 | ,375 | ,957 |
| 145 | 14,998 | ,378 | ,956 |
| 99 | 14,901 | ,385 | ,964 |
| 153 | 14,873 | ,387 | ,959 |
| 93 | 14,815 | ,391 | ,960 |
| 30 | 14,737 | ,396 | ,965 |
| 113 | 14,721 | ,398 | ,958 |
| 227 | 14,687 | ,400 | ,954 |
| 242 | 14,638 | ,403 | ,953 |
| 34 | 14,594 | ,406 | ,951 |

##### Sample Moments (Group number 1)

##### Sample Covariances (Group number 1)

|  | UpdatingCombined | Creativity\_TCTDP | MathKnowledge\_Cito | InhibitionIncongruentRT | MeanRTShift | URC\_Org\_4 | URC\_Flex\_4 | URC\_Flu\_4 | URC\_Org\_3 | URC\_Flex\_3 | URC\_Flu\_3 | URC\_Org\_1 | URC\_Flex\_1 | URC\_Flu\_1 |
| --- | --- | --- | --- | --- | --- | --- | --- | --- | --- | --- | --- | --- | --- | --- |
| UpdatingCombined | ,694 |
| Creativity\_TCTDP | 1,802 | 91,437 |
| MathKnowledge\_Cito | ,158 | ,160 | ,998 |
| InhibitionIncongruentRT | -47,457 | -94,518 | -9,985 | 22972,423 |
| MeanRTShift | -16,644 | -208,923 | -20,132 | 11910,364 | 55729,438 |
| URC\_Org\_4 | ,025 | ,406 | ,028 | -2,853 | -3,968 | ,085 |
| URC\_Flex\_4 | ,201 | 1,088 | ,150 | -22,641 | -13,859 | ,168 | ,993 |
| URC\_Flu\_4 | ,331 | 2,223 | ,164 | -38,698 | -27,877 | ,327 | 1,252 | 2,478 |
| URC\_Org\_3 | ,058 | ,243 | ,013 | -7,996 | -4,379 | ,010 | ,065 | ,099 | ,053 |
| URC\_Flex\_3 | ,206 | ,865 | ,133 | -19,051 | -10,955 | ,038 | ,240 | ,382 | ,117 | ,730 |
| URC\_Flu\_3 | 2,427 | 7,635 | ,436 | -335,670 | -187,023 | ,354 | 1,718 | 2,827 | ,627 | 2,412 | 74,667 |
| URC\_Org\_1 | ,057 | ,156 | ,023 | -4,669 | -4,565 | ,021 | ,061 | ,137 | ,020 | ,050 | ,614 | ,115 |
| URC\_Flex\_1 | ,224 | 1,335 | ,121 | -26,947 | -14,716 | ,051 | ,218 | ,346 | ,037 | ,101 | 1,616 | ,134 | ,575 |
| URC\_Flu\_1 | ,426 | 2,130 | ,181 | -35,352 | -41,604 | ,120 | ,442 | ,787 | ,090 | ,295 | 3,001 | ,324 | ,788 | 2,067 |

Condition number = 1988132,409

Eigenvalues

59604,478 19103,527 92,505 68,237 3,568 1,761 ,999 ,608 ,442 ,283 ,215 ,061 ,040 ,030

Determinant of sample covariance matrix = 53559569,850

##### Sample Correlations (Group number 1)

|  | UpdatingCombined | Creativity\_TCTDP | MathKnowledge\_Cito | InhibitionIncongruentRT | MeanRTShift | URC\_Org\_4 | URC\_Flex\_4 | URC\_Flu\_4 | URC\_Org\_3 | URC\_Flex\_3 | URC\_Flu\_3 | URC\_Org\_1 | URC\_Flex\_1 | URC\_Flu\_1 |
| --- | --- | --- | --- | --- | --- | --- | --- | --- | --- | --- | --- | --- | --- | --- |
| UpdatingCombined | 1,000 |
| Creativity\_TCTDP | ,226 | 1,000 |
| MathKnowledge\_Cito | ,189 | ,017 | 1,000 |
| InhibitionIncongruentRT | -,376 | -,065 | -,066 | 1,000 |
| MeanRTShift | -,085 | -,093 | -,085 | ,333 | 1,000 |
| URC\_Org\_4 | ,104 | ,145 | ,096 | -,065 | -,058 | 1,000 |
| URC\_Flex\_4 | ,242 | ,114 | ,150 | -,150 | -,059 | ,577 | 1,000 |
| URC\_Flu\_4 | ,253 | ,148 | ,104 | -,162 | -,075 | ,712 | ,798 | 1,000 |
| URC\_Org\_3 | ,303 | ,110 | ,057 | -,229 | -,080 | ,147 | ,282 | ,274 | 1,000 |
| URC\_Flex\_3 | ,290 | ,106 | ,156 | -,147 | -,054 | ,151 | ,282 | ,284 | ,592 | 1,000 |
| URC\_Flu\_3 | ,337 | ,092 | ,051 | -,256 | -,092 | ,140 | ,199 | ,208 | ,315 | ,327 | 1,000 |
| URC\_Org\_1 | ,201 | ,048 | ,067 | -,091 | -,057 | ,213 | ,181 | ,256 | ,257 | ,172 | ,210 | 1,000 |
| URC\_Flex\_1 | ,355 | ,184 | ,160 | -,234 | -,082 | ,231 | ,289 | ,290 | ,213 | ,155 | ,247 | ,521 | 1,000 |
| URC\_Flu\_1 | ,356 | ,155 | ,126 | -,162 | -,123 | ,287 | ,308 | ,348 | ,270 | ,240 | ,242 | ,665 | ,723 | 1,000 |

Condition number = 24,772

Eigenvalues

4,115 1,640 1,442 1,201 ,996 ,978 ,840 ,687 ,496 ,451 ,393 ,371 ,225 ,166

##### Sample Means (Group number 1)

|  | UpdatingCombined | Creativity\_TCTDP | MathKnowledge\_Cito | InhibitionIncongruentRT | MeanRTShift | URC\_Org\_4 | URC\_Flex\_4 | URC\_Flu\_4 | URC\_Org\_3 | URC\_Flex\_3 | URC\_Flu\_3 | URC\_Org\_1 | URC\_Flex\_1 | URC\_Flu\_1 |
| --- | --- | --- | --- | --- | --- | --- | --- | --- | --- | --- | --- | --- | --- | --- |
|  | ,036 | 20,331 | ,006 | 790,082 | 1386,833 | ,498 | 1,777 | 2,306 | ,417 | 2,255 | 8,331 | ,551 | 1,705 | 2,367 |

##### Models

##### Default model (Default model)

##### Notes for Model (Default model)

##### Computation of degrees of freedom (Default model)

|  |  |
| --- | --- |
| Number of distinct sample moments: | 119 |
| Number of distinct parameters to be estimated: | 59 |
| Degrees of freedom (119 - 59): | 60 |

##### Result (Default model)

Minimum was achieved

Chi-square = 72,640

Degrees of freedom = 60

Probability level = ,127

##### Group number 1 (Group number 1 - Default model)

##### Estimates (Group number 1 - Default model)

##### Scalar Estimates (Group number 1 - Default model)

##### Maximum Likelihood Estimates

##### Regression Weights: (Group number 1 - Default model)

|  |  |  | Estimate | S.E. | C.R. | P | Label |
| --- | --- | --- | --- | --- | --- | --- | --- |
| Creativity\_TCTDP | <--- | UpdatingCombined | 2,596 | ,672 | 3,865 | \*\*\* | par\_14 |
| MathKnowledge\_Cito | <--- | UpdatingCombined | ,227 | ,071 | 3,212 | ,001 | par\_15 |
| Math\_DT | <--- | MathKnowledge\_Cito | ,089 | ,061 | 1,476 | ,140 | par\_12 |
| Math\_DT | <--- | Creativity\_TCTDP | ,012 | ,006 | 1,887 | ,059 | par\_13 |
| Math\_DT | <--- | InhibitionIncongruentRT | -,001 | ,000 | -1,564 | ,118 | par\_16 |
| Math\_DT | <--- | MeanRTShift | ,000 | ,000 | -,878 | ,380 | par\_17 |
| Math\_DT | <--- | UpdatingCombined | ,497 | ,090 | 5,512 | \*\*\* | par\_18 |
| Task\_1 | <--- | Math\_DT | 1,000 |  |
| Task\_3 | <--- | Math\_DT | 3,076 | ,645 | 4,767 | \*\*\* | par\_1 |
| Task\_4 | <--- | Math\_DT | ,978 | ,165 | 5,910 | \*\*\* | par\_2 |
| URC\_Flu\_1 | <--- | Task\_1 | 1,000 |  |
| URC\_Flex\_1 | <--- | Task\_1 | ,419 | ,030 | 13,842 | \*\*\* | par\_3 |
| URC\_Org\_1 | <--- | Task\_1 | ,173 | ,014 | 12,603 | \*\*\* | par\_4 |
| URC\_Flu\_3 | <--- | Task\_3 | 1,000 |  |
| URC\_Flex\_3 | <--- | Task\_3 | ,162 | ,025 | 6,428 | \*\*\* | par\_5 |
| URC\_Org\_3 | <--- | Task\_3 | ,044 | ,007 | 6,438 | \*\*\* | par\_6 |
| URC\_Flu\_4 | <--- | Task\_4 | 1,000 |  |
| URC\_Flex\_4 | <--- | Task\_4 | ,518 | ,030 | 17,092 | \*\*\* | par\_7 |
| URC\_Org\_4 | <--- | Task\_4 | ,135 | ,009 | 14,363 | \*\*\* | par\_8 |

##### Standardized Regression Weights: (Group number 1 - Default model)

|  |  |  | Estimate |
| --- | --- | --- | --- |
| Creativity\_TCTDP | <--- | UpdatingCombined | ,226 |
| MathKnowledge\_Cito | <--- | UpdatingCombined | ,189 |
| Math\_DT | <--- | MathKnowledge\_Cito | ,102 |
| Math\_DT | <--- | Creativity\_TCTDP | ,132 |
| Math\_DT | <--- | InhibitionIncongruentRT | -,121 |
| Math\_DT | <--- | MeanRTShift | -,063 |
| Math\_DT | <--- | UpdatingCombined | ,475 |
| Task\_1 | <--- | Math\_DT | ,638 |
| Task\_3 | <--- | Math\_DT | ,677 |
| Task\_4 | <--- | Math\_DT | ,549 |
| URC\_Flu\_1 | <--- | Task\_1 | ,950 |
| URC\_Flex\_1 | <--- | Task\_1 | ,760 |
| URC\_Org\_1 | <--- | Task\_1 | ,699 |
| URC\_Flu\_3 | <--- | Task\_3 | ,460 |
| URC\_Flex\_3 | <--- | Task\_3 | ,754 |
| URC\_Org\_3 | <--- | Task\_3 | ,755 |
| URC\_Flu\_4 | <--- | Task\_4 | ,987 |
| URC\_Flex\_4 | <--- | Task\_4 | ,809 |
| URC\_Org\_4 | <--- | Task\_4 | ,722 |

##### Intercepts: (Group number 1 - Default model)

|  |  |  | Estimate | S.E. | C.R. | P | Label |
| --- | --- | --- | --- | --- | --- | --- | --- |
| UpdatingCombined |  |  | ,036 | ,050 | ,728 | ,467 | par\_29 |
| MeanRTShift |  |  | 1386,833 | 14,184 | 97,774 | \*\*\* | par\_28 |
| InhibitionIncongruentRT |  |  | 790,082 | 9,107 | 86,758 | \*\*\* | par\_30 |
| Creativity\_TCTDP |  |  | 20,236 | ,560 | 36,124 | \*\*\* | par\_31 |
| MathKnowledge\_Cito |  |  | -,002 | ,059 | -,031 | ,976 | par\_32 |
| URC\_Flu\_1 |  |  | 2,979 | ,447 | 6,663 | \*\*\* | par\_33 |
| URC\_Flex\_1 |  |  | 1,961 | ,190 | 10,331 | \*\*\* | par\_34 |
| URC\_Org\_1 |  |  | ,657 | ,079 | 8,314 | \*\*\* | par\_35 |
| URC\_Flu\_3 |  |  | 10,212 | 1,468 | 6,958 | \*\*\* | par\_36 |
| URC\_Flex\_3 |  |  | 2,560 | ,226 | 11,346 | \*\*\* | par\_37 |
| URC\_Org\_3 |  |  | ,499 | ,061 | 8,185 | \*\*\* | par\_38 |
| URC\_Flu\_4 |  |  | 2,904 | ,441 | 6,586 | \*\*\* | par\_39 |
| URC\_Flex\_4 |  |  | 2,087 | ,232 | 9,008 | \*\*\* | par\_40 |
| URC\_Org\_4 |  |  | ,579 | ,061 | 9,481 | \*\*\* | par\_41 |

##### Covariances: (Group number 1 - Default model)

|  |  |  | Estimate | S.E. | C.R. | P | Label |
| --- | --- | --- | --- | --- | --- | --- | --- |
| e1 | <--> | e3 | 11910,364 | 2265,816 | 5,257 | \*\*\* | par\_9 |
| e3 | <--> | e2 | -47,457 | 8,105 | -5,855 | \*\*\* | par\_10 |
| e1 | <--> | e2 | -16,644 | 11,860 | -1,403 | ,161 | par\_11 |
| e6 | <--> | e9 | -,299 | ,409 | -,730 | ,465 | par\_19 |
| e6 | <--> | e12 | -,052 | ,047 | -1,102 | ,271 | par\_20 |
| e9 | <--> | e12 | -,099 | ,388 | -,256 | ,798 | par\_21 |
| e7 | <--> | e10 | -,036 | ,021 | -1,699 | ,089 | par\_22 |
| e7 | <--> | e13 | ,037 | ,020 | 1,835 | ,067 | par\_23 |
| e10 | <--> | e13 | ,015 | ,024 | ,619 | ,536 | par\_24 |
| e8 | <--> | e11 | ,005 | ,003 | 1,930 | ,054 | par\_25 |
| e8 | <--> | e14 | ,002 | ,003 | ,528 | ,597 | par\_26 |
| e11 | <--> | e14 | -,002 | ,002 | -,707 | ,480 | par\_27 |

##### Correlations: (Group number 1 - Default model)

|  |  |  | Estimate |
| --- | --- | --- | --- |
| e1 | <--> | e3 | ,333 |
| e3 | <--> | e2 | -,376 |
| e1 | <--> | e2 | -,085 |
| e6 | <--> | e9 | -,086 |
| e6 | <--> | e12 | -,444 |
| e9 | <--> | e12 | -,050 |
| e7 | <--> | e10 | -,132 |
| e7 | <--> | e13 | ,130 |
| e10 | <--> | e13 | ,046 |
| e8 | <--> | e11 | ,146 |
| e8 | <--> | e14 | ,035 |
| e11 | <--> | e14 | -,052 |

##### Variances: (Group number 1 - Default model)

|  |  |  | Estimate | S.E. | C.R. | P | Label |
| --- | --- | --- | --- | --- | --- | --- | --- |
| e2 |  |  | ,694 | ,059 | 11,769 | \*\*\* | par\_42 |
| e1 |  |  | 55729,438 | 4735,434 | 11,769 | \*\*\* | par\_43 |
| e3 |  |  | 22972,423 | 1952,009 | 11,769 | \*\*\* | par\_44 |
| e5 |  |  | ,962 | ,082 | 11,769 | \*\*\* | par\_45 |
| e4 |  |  | 86,759 | 7,372 | 11,769 | \*\*\* | par\_46 |
| e15 |  |  | ,474 | ,124 | 3,835 | \*\*\* | par\_47 |
| e16 |  |  | 1,113 | ,175 | 6,370 | \*\*\* | par\_48 |
| e17 |  |  | 8,549 | 2,727 | 3,135 | ,002 | par\_49 |
| e18 |  |  | 1,693 | ,208 | 8,131 | \*\*\* | par\_50 |
| e6 |  |  | ,205 | ,094 | 2,170 | ,030 | par\_51 |
| e8 |  |  | ,059 | ,006 | 10,195 | \*\*\* | par\_52 |
| e7 |  |  | ,240 | ,026 | 9,126 | \*\*\* | par\_53 |
| e9 |  |  | 58,792 | 5,449 | 10,789 | \*\*\* | par\_54 |
| e10 |  |  | ,315 | ,049 | 6,383 | \*\*\* | par\_55 |
| e11 |  |  | ,023 | ,004 | 6,374 | \*\*\* | par\_56 |
| e13 |  |  | ,342 | ,038 | 8,900 | \*\*\* | par\_57 |
| e12 |  |  | ,066 | ,094 | ,706 | ,480 | par\_58 |
| e14 |  |  | ,041 | ,004 | 10,534 | \*\*\* | par\_59 |

##### Squared Multiple Correlations: (Group number 1 - Default model)

|  |  |  | Estimate |
| --- | --- | --- | --- |
| UpdatingCombined |  |  | ,000 |
| Creativity\_TCTDP |  |  | ,051 |
| MathKnowledge\_Cito |  |  | ,036 |
| InhibitionIncongruentRT |  |  | ,000 |
| MeanRTShift |  |  | ,000 |
| Math\_DT |  |  | ,378 |
| Task\_4 |  |  | ,301 |
| Task\_3 |  |  | ,458 |
| Task\_1 |  |  | ,407 |
| URC\_Org\_4 |  |  | ,521 |
| URC\_Flex\_4 |  |  | ,655 |
| URC\_Flu\_4 |  |  | ,973 |
| URC\_Org\_3 |  |  | ,570 |
| URC\_Flex\_3 |  |  | ,568 |
| URC\_Flu\_3 |  |  | ,211 |
| URC\_Org\_1 |  |  | ,489 |
| URC\_Flex\_1 |  |  | ,578 |
| URC\_Flu\_1 |  |  | ,902 |

##### Matrices (Group number 1 - Default model)

##### Implied Covariances (Group number 1 - Default model)

|  | UpdatingCombined | Creativity\_TCTDP | MathKnowledge\_Cito | InhibitionIncongruentRT | MeanRTShift | URC\_Org\_4 | URC\_Flex\_4 | URC\_Flu\_4 | URC\_Org\_3 | URC\_Flex\_3 | URC\_Flu\_3 | URC\_Org\_1 | URC\_Flex\_1 | URC\_Flu\_1 |
| --- | --- | --- | --- | --- | --- | --- | --- | --- | --- | --- | --- | --- | --- | --- |
| UpdatingCombined | ,694 |
| Creativity\_TCTDP | 1,802 | 91,437 |
| MathKnowledge\_Cito | ,158 | ,409 | ,998 |
| InhibitionIncongruentRT | -47,457 | -123,203 | -10,780 | 22972,423 |
| MeanRTShift | -16,644 | -43,209 | -3,781 | 11910,364 | 55729,438 |
| URC\_Org\_4 | ,055 | ,282 | ,024 | -5,939 | -4,029 | ,085 |
| URC\_Flex\_4 | ,212 | 1,082 | ,092 | -22,760 | -15,440 | ,170 | ,993 |
| URC\_Flu\_4 | ,409 | 2,087 | ,177 | -43,917 | -29,792 | ,328 | 1,256 | 2,489 |
| URC\_Org\_3 | ,056 | ,288 | ,024 | -6,050 | -4,104 | ,012 | ,052 | ,100 | ,053 |
| URC\_Flex\_3 | ,209 | 1,064 | ,090 | -22,391 | -15,189 | ,050 | ,208 | ,372 | ,112 | ,729 |
| URC\_Flu\_3 | 1,286 | 6,564 | ,557 | -138,115 | -93,693 | ,310 | 1,189 | 2,195 | ,690 | 2,556 | 74,555 |
| URC\_Org\_1 | ,072 | ,370 | ,031 | -7,783 | -5,280 | ,019 | ,067 | ,129 | ,023 | ,066 | ,407 | ,115 |
| URC\_Flex\_1 | ,175 | ,894 | ,076 | -18,814 | -12,763 | ,042 | ,199 | ,313 | ,043 | ,123 | ,983 | ,136 | ,569 |
| URC\_Flu\_1 | ,418 | 2,134 | ,181 | -44,907 | -30,463 | ,101 | ,387 | ,694 | ,103 | ,380 | 2,047 | ,325 | ,786 | 2,080 |

##### Implied Correlations (Group number 1 - Default model)

|  | UpdatingCombined | Creativity\_TCTDP | MathKnowledge\_Cito | InhibitionIncongruentRT | MeanRTShift | URC\_Org\_4 | URC\_Flex\_4 | URC\_Flu\_4 | URC\_Org\_3 | URC\_Flex\_3 | URC\_Flu\_3 | URC\_Org\_1 | URC\_Flex\_1 | URC\_Flu\_1 |
| --- | --- | --- | --- | --- | --- | --- | --- | --- | --- | --- | --- | --- | --- | --- |
| UpdatingCombined | 1,000 |
| Creativity\_TCTDP | ,226 | 1,000 |
| MathKnowledge\_Cito | ,189 | ,043 | 1,000 |
| InhibitionIncongruentRT | -,376 | -,085 | -,071 | 1,000 |
| MeanRTShift | -,085 | -,019 | -,016 | ,333 | 1,000 |
| URC\_Org\_4 | ,228 | ,101 | ,082 | -,134 | -,058 | 1,000 |
| URC\_Flex\_4 | ,255 | ,114 | ,092 | -,151 | -,066 | ,584 | 1,000 |
| URC\_Flu\_4 | ,311 | ,138 | ,112 | -,184 | -,080 | ,712 | ,799 | 1,000 |
| URC\_Org\_3 | ,294 | ,131 | ,106 | -,173 | -,075 | ,179 | ,227 | ,276 | 1,000 |
| URC\_Flex\_3 | ,293 | ,130 | ,106 | -,173 | -,075 | ,202 | ,244 | ,276 | ,569 | 1,000 |
| URC\_Flu\_3 | ,179 | ,080 | ,065 | -,106 | -,046 | ,123 | ,138 | ,161 | ,347 | ,347 | 1,000 |
| URC\_Org\_1 | ,256 | ,114 | ,093 | -,151 | -,066 | ,194 | ,198 | ,242 | ,296 | ,228 | ,139 | 1,000 |
| URC\_Flex\_1 | ,279 | ,124 | ,101 | -,164 | -,072 | ,192 | ,265 | ,262 | ,248 | ,191 | ,151 | ,532 | 1,000 |
| URC\_Flu\_1 | ,348 | ,155 | ,126 | -,205 | -,089 | ,240 | ,269 | ,305 | ,309 | ,309 | ,164 | ,664 | ,722 | 1,000 |

##### Implied Means (Group number 1 - Default model)

|  | UpdatingCombined | Creativity\_TCTDP | MathKnowledge\_Cito | InhibitionIncongruentRT | MeanRTShift | URC\_Org\_4 | URC\_Flex\_4 | URC\_Flu\_4 | URC\_Org\_3 | URC\_Flex\_3 | URC\_Flu\_3 | URC\_Org\_1 | URC\_Flex\_1 | URC\_Flu\_1 |
| --- | --- | --- | --- | --- | --- | --- | --- | --- | --- | --- | --- | --- | --- | --- |
|  | ,036 | 20,331 | ,006 | 790,082 | 1386,833 | ,498 | 1,777 | 2,306 | ,417 | 2,255 | 8,331 | ,551 | 1,705 | 2,367 |

##### Residual Covariances (Group number 1 - Default model)

|  | UpdatingCombined | Creativity\_TCTDP | MathKnowledge\_Cito | InhibitionIncongruentRT | MeanRTShift | URC\_Org\_4 | URC\_Flex\_4 | URC\_Flu\_4 | URC\_Org\_3 | URC\_Flex\_3 | URC\_Flu\_3 | URC\_Org\_1 | URC\_Flex\_1 | URC\_Flu\_1 |
| --- | --- | --- | --- | --- | --- | --- | --- | --- | --- | --- | --- | --- | --- | --- |
| UpdatingCombined | ,000 |
| Creativity\_TCTDP | ,000 | ,000 |
| MathKnowledge\_Cito | ,000 | -,249 | ,000 |
| InhibitionIncongruentRT | ,000 | 28,685 | ,796 | ,000 |
| MeanRTShift | ,000 | -165,714 | -16,351 | ,000 | ,000 |
| URC\_Org\_4 | -,030 | ,123 | ,004 | 3,086 | ,061 | ,000 |
| URC\_Flex\_4 | -,011 | ,006 | ,058 | ,119 | 1,581 | -,002 | ,000 |
| URC\_Flu\_4 | -,078 | ,135 | -,013 | 5,219 | 1,914 | -,001 | -,004 | -,011 |
| URC\_Org\_3 | ,002 | -,045 | -,011 | -1,947 | -,275 | -,002 | ,013 | -,001 | ,000 |
| URC\_Flex\_3 | -,002 | -,199 | ,043 | 3,340 | 4,235 | -,013 | ,033 | ,010 | ,005 | ,001 |
| URC\_Flu\_3 | 1,141 | 1,071 | -,121 | -197,556 | -93,330 | ,044 | ,529 | ,632 | -,064 | -,144 | ,112 |
| URC\_Org\_1 | -,016 | -,213 | -,009 | 3,114 | ,715 | ,002 | -,006 | ,007 | -,003 | -,016 | ,207 | ,000 |
| URC\_Flex\_1 | ,049 | ,441 | ,045 | -8,133 | -1,954 | ,009 | ,019 | ,033 | -,006 | -,023 | ,633 | -,002 | ,005 |
| URC\_Flu\_1 | ,008 | -,004 | ,000 | 9,555 | -11,141 | ,019 | ,055 | ,093 | -,013 | -,086 | ,954 | -,001 | ,002 | -,013 |

##### Residual Means (Group number 1 - Default model)

|  | UpdatingCombined | Creativity\_TCTDP | MathKnowledge\_Cito | InhibitionIncongruentRT | MeanRTShift | URC\_Org\_4 | URC\_Flex\_4 | URC\_Flu\_4 | URC\_Org\_3 | URC\_Flex\_3 | URC\_Flu\_3 | URC\_Org\_1 | URC\_Flex\_1 | URC\_Flu\_1 |
| --- | --- | --- | --- | --- | --- | --- | --- | --- | --- | --- | --- | --- | --- | --- |
|  | ,000 | ,000 | ,000 | ,000 | ,000 | ,000 | ,000 | ,000 | ,000 | ,000 | ,000 | ,000 | ,000 | ,000 |

##### Standardized Residual Covariances (Group number 1 - Default model)

|  | UpdatingCombined | Creativity\_TCTDP | MathKnowledge\_Cito | InhibitionIncongruentRT | MeanRTShift | URC\_Org\_4 | URC\_Flex\_4 | URC\_Flu\_4 | URC\_Org\_3 | URC\_Flex\_3 | URC\_Flu\_3 | URC\_Org\_1 | URC\_Flex\_1 | URC\_Flu\_1 |
| --- | --- | --- | --- | --- | --- | --- | --- | --- | --- | --- | --- | --- | --- | --- |
| UpdatingCombined | ,000 |
| Creativity\_TCTDP | ,000 | ,000 |
| MathKnowledge\_Cito | ,000 | -,434 | ,000 |
| InhibitionIncongruentRT | ,000 | ,328 | ,087 | ,000 |
| MeanRTShift | ,000 | -1,222 | -1,154 | ,000 | ,000 |
| URC\_Org\_4 | -2,004 | ,733 | ,237 | 1,151 | ,015 | ,001 |
| URC\_Flex\_4 | -,217 | ,011 | ,965 | ,013 | ,112 | -,095 | ,003 |
| URC\_Flu\_4 | -,937 | ,148 | -,138 | ,357 | ,085 | -,020 | -,033 | -,050 |
| URC\_Org\_3 | ,155 | -,336 | -,814 | -,914 | -,084 | -,514 | ,902 | -,054 | ,007 |
| URC\_Flex\_3 | -,047 | -,402 | ,830 | ,423 | ,349 | -,831 | ,618 | ,124 | ,346 | ,015 |
| URC\_Flu\_3 | 2,598 | ,215 | -,232 | -2,499 | -,761 | ,287 | 1,013 | ,762 | -,502 | -,307 | ,018 |
| URC\_Org\_1 | -,899 | -1,088 | -,429 | ,996 | ,148 | ,309 | -,288 | ,219 | -,628 | -,897 | 1,167 | -,042 |
| URC\_Flex\_1 | 1,250 | 1,009 | ,989 | -1,168 | -,182 | ,661 | ,409 | ,447 | -,538 | -,573 | 1,598 | -,138 | ,112 |
| URC\_Flu\_1 | ,104 | -,005 | ,003 | ,713 | -,542 | ,744 | ,615 | ,650 | -,629 | -1,105 | 1,258 | -,038 | ,029 | -,075 |

##### Standardized Residual Means (Group number 1 - Default model)

|  | UpdatingCombined | Creativity\_TCTDP | MathKnowledge\_Cito | InhibitionIncongruentRT | MeanRTShift | URC\_Org\_4 | URC\_Flex\_4 | URC\_Flu\_4 | URC\_Org\_3 | URC\_Flex\_3 | URC\_Flu\_3 | URC\_Org\_1 | URC\_Flex\_1 | URC\_Flu\_1 |
| --- | --- | --- | --- | --- | --- | --- | --- | --- | --- | --- | --- | --- | --- | --- |
|  | ,000 | ,000 | ,000 | ,000 | ,000 | ,000 | ,000 | ,000 | ,000 | ,000 | ,000 | ,000 | ,000 | ,000 |

##### Total Effects (Group number 1 - Default model)

|  | UpdatingCombined | Creativity\_TCTDP | MathKnowledge\_Cito | InhibitionIncongruentRT | MeanRTShift | Math\_DT | Task\_4 | Task\_3 | Task\_1 |
| --- | --- | --- | --- | --- | --- | --- | --- | --- | --- |
| Creativity\_TCTDP | 2,596 | ,000 | ,000 | ,000 | ,000 | ,000 | ,000 | ,000 | ,000 |
| MathKnowledge\_Cito | ,227 | ,000 | ,000 | ,000 | ,000 | ,000 | ,000 | ,000 | ,000 |
| Math\_DT | ,549 | ,012 | ,089 | -,001 | ,000 | ,000 | ,000 | ,000 | ,000 |
| Task\_4 | ,537 | ,012 | ,087 | -,001 | ,000 | ,978 | ,000 | ,000 | ,000 |
| Task\_3 | 1,689 | ,037 | ,275 | -,002 | -,001 | 3,076 | ,000 | ,000 | ,000 |
| Task\_1 | ,549 | ,012 | ,089 | -,001 | ,000 | 1,000 | ,000 | ,000 | ,000 |
| URC\_Org\_4 | ,073 | ,002 | ,012 | ,000 | ,000 | ,132 | ,135 | ,000 | ,000 |
| URC\_Flex\_4 | ,278 | ,006 | ,045 | ,000 | ,000 | ,507 | ,518 | ,000 | ,000 |
| URC\_Flu\_4 | ,537 | ,012 | ,087 | -,001 | ,000 | ,978 | 1,000 | ,000 | ,000 |
| URC\_Org\_3 | ,074 | ,002 | ,012 | ,000 | ,000 | ,135 | ,000 | ,044 | ,000 |
| URC\_Flex\_3 | ,274 | ,006 | ,045 | ,000 | ,000 | ,499 | ,000 | ,162 | ,000 |
| URC\_Flu\_3 | 1,689 | ,037 | ,275 | -,002 | -,001 | 3,076 | ,000 | 1,000 | ,000 |
| URC\_Org\_1 | ,095 | ,002 | ,015 | ,000 | ,000 | ,173 | ,000 | ,000 | ,173 |
| URC\_Flex\_1 | ,230 | ,005 | ,037 | ,000 | ,000 | ,419 | ,000 | ,000 | ,419 |
| URC\_Flu\_1 | ,549 | ,012 | ,089 | -,001 | ,000 | 1,000 | ,000 | ,000 | 1,000 |

##### Standardized Total Effects (Group number 1 - Default model)

|  | UpdatingCombined | Creativity\_TCTDP | MathKnowledge\_Cito | InhibitionIncongruentRT | MeanRTShift | Math\_DT | Task\_4 | Task\_3 | Task\_1 |
| --- | --- | --- | --- | --- | --- | --- | --- | --- | --- |
| Creativity\_TCTDP | ,226 | ,000 | ,000 | ,000 | ,000 | ,000 | ,000 | ,000 | ,000 |
| MathKnowledge\_Cito | ,189 | ,000 | ,000 | ,000 | ,000 | ,000 | ,000 | ,000 | ,000 |
| Math\_DT | ,524 | ,132 | ,102 | -,121 | -,063 | ,000 | ,000 | ,000 | ,000 |
| Task\_4 | ,287 | ,073 | ,056 | -,067 | -,035 | ,549 | ,000 | ,000 | ,000 |
| Task\_3 | ,354 | ,090 | ,069 | -,082 | -,043 | ,677 | ,000 | ,000 | ,000 |
| Task\_1 | ,334 | ,084 | ,065 | -,077 | -,040 | ,638 | ,000 | ,000 | ,000 |
| URC\_Org\_4 | ,207 | ,052 | ,040 | -,048 | -,025 | ,396 | ,722 | ,000 | ,000 |
| URC\_Flex\_4 | ,233 | ,059 | ,045 | -,054 | -,028 | ,444 | ,809 | ,000 | ,000 |
| URC\_Flu\_4 | ,284 | ,072 | ,055 | -,066 | -,034 | ,541 | ,987 | ,000 | ,000 |
| URC\_Org\_3 | ,267 | ,068 | ,052 | -,062 | -,032 | ,511 | ,000 | ,755 | ,000 |
| URC\_Flex\_3 | ,267 | ,067 | ,052 | -,062 | -,032 | ,510 | ,000 | ,754 | ,000 |
| URC\_Flu\_3 | ,163 | ,041 | ,032 | -,038 | -,020 | ,311 | ,000 | ,460 | ,000 |
| URC\_Org\_1 | ,234 | ,059 | ,046 | -,054 | -,028 | ,446 | ,000 | ,000 | ,699 |
| URC\_Flex\_1 | ,254 | ,064 | ,050 | -,059 | -,031 | ,485 | ,000 | ,000 | ,760 |
| URC\_Flu\_1 | ,317 | ,080 | ,062 | -,074 | -,038 | ,606 | ,000 | ,000 | ,950 |

##### Direct Effects (Group number 1 - Default model)

|  | UpdatingCombined | Creativity\_TCTDP | MathKnowledge\_Cito | InhibitionIncongruentRT | MeanRTShift | Math\_DT | Task\_4 | Task\_3 | Task\_1 |
| --- | --- | --- | --- | --- | --- | --- | --- | --- | --- |
| Creativity\_TCTDP | 2,596 | ,000 | ,000 | ,000 | ,000 | ,000 | ,000 | ,000 | ,000 |
| MathKnowledge\_Cito | ,227 | ,000 | ,000 | ,000 | ,000 | ,000 | ,000 | ,000 | ,000 |
| Math\_DT | ,497 | ,012 | ,089 | -,001 | ,000 | ,000 | ,000 | ,000 | ,000 |
| Task\_4 | ,000 | ,000 | ,000 | ,000 | ,000 | ,978 | ,000 | ,000 | ,000 |
| Task\_3 | ,000 | ,000 | ,000 | ,000 | ,000 | 3,076 | ,000 | ,000 | ,000 |
| Task\_1 | ,000 | ,000 | ,000 | ,000 | ,000 | 1,000 | ,000 | ,000 | ,000 |
| URC\_Org\_4 | ,000 | ,000 | ,000 | ,000 | ,000 | ,000 | ,135 | ,000 | ,000 |
| URC\_Flex\_4 | ,000 | ,000 | ,000 | ,000 | ,000 | ,000 | ,518 | ,000 | ,000 |
| URC\_Flu\_4 | ,000 | ,000 | ,000 | ,000 | ,000 | ,000 | 1,000 | ,000 | ,000 |
| URC\_Org\_3 | ,000 | ,000 | ,000 | ,000 | ,000 | ,000 | ,000 | ,044 | ,000 |
| URC\_Flex\_3 | ,000 | ,000 | ,000 | ,000 | ,000 | ,000 | ,000 | ,162 | ,000 |
| URC\_Flu\_3 | ,000 | ,000 | ,000 | ,000 | ,000 | ,000 | ,000 | 1,000 | ,000 |
| URC\_Org\_1 | ,000 | ,000 | ,000 | ,000 | ,000 | ,000 | ,000 | ,000 | ,173 |
| URC\_Flex\_1 | ,000 | ,000 | ,000 | ,000 | ,000 | ,000 | ,000 | ,000 | ,419 |
| URC\_Flu\_1 | ,000 | ,000 | ,000 | ,000 | ,000 | ,000 | ,000 | ,000 | 1,000 |

##### Standardized Direct Effects (Group number 1 - Default model)

|  | UpdatingCombined | Creativity\_TCTDP | MathKnowledge\_Cito | InhibitionIncongruentRT | MeanRTShift | Math\_DT | Task\_4 | Task\_3 | Task\_1 |
| --- | --- | --- | --- | --- | --- | --- | --- | --- | --- |
| Creativity\_TCTDP | ,226 | ,000 | ,000 | ,000 | ,000 | ,000 | ,000 | ,000 | ,000 |
| MathKnowledge\_Cito | ,189 | ,000 | ,000 | ,000 | ,000 | ,000 | ,000 | ,000 | ,000 |
| Math\_DT | ,475 | ,132 | ,102 | -,121 | -,063 | ,000 | ,000 | ,000 | ,000 |
| Task\_4 | ,000 | ,000 | ,000 | ,000 | ,000 | ,549 | ,000 | ,000 | ,000 |
| Task\_3 | ,000 | ,000 | ,000 | ,000 | ,000 | ,677 | ,000 | ,000 | ,000 |
| Task\_1 | ,000 | ,000 | ,000 | ,000 | ,000 | ,638 | ,000 | ,000 | ,000 |
| URC\_Org\_4 | ,000 | ,000 | ,000 | ,000 | ,000 | ,000 | ,722 | ,000 | ,000 |
| URC\_Flex\_4 | ,000 | ,000 | ,000 | ,000 | ,000 | ,000 | ,809 | ,000 | ,000 |
| URC\_Flu\_4 | ,000 | ,000 | ,000 | ,000 | ,000 | ,000 | ,987 | ,000 | ,000 |
| URC\_Org\_3 | ,000 | ,000 | ,000 | ,000 | ,000 | ,000 | ,000 | ,755 | ,000 |
| URC\_Flex\_3 | ,000 | ,000 | ,000 | ,000 | ,000 | ,000 | ,000 | ,754 | ,000 |
| URC\_Flu\_3 | ,000 | ,000 | ,000 | ,000 | ,000 | ,000 | ,000 | ,460 | ,000 |
| URC\_Org\_1 | ,000 | ,000 | ,000 | ,000 | ,000 | ,000 | ,000 | ,000 | ,699 |
| URC\_Flex\_1 | ,000 | ,000 | ,000 | ,000 | ,000 | ,000 | ,000 | ,000 | ,760 |
| URC\_Flu\_1 | ,000 | ,000 | ,000 | ,000 | ,000 | ,000 | ,000 | ,000 | ,950 |

##### Indirect Effects (Group number 1 - Default model)

|  | UpdatingCombined | Creativity\_TCTDP | MathKnowledge\_Cito | InhibitionIncongruentRT | MeanRTShift | Math\_DT | Task\_4 | Task\_3 | Task\_1 |
| --- | --- | --- | --- | --- | --- | --- | --- | --- | --- |
| Creativity\_TCTDP | ,000 | ,000 | ,000 | ,000 | ,000 | ,000 | ,000 | ,000 | ,000 |
| MathKnowledge\_Cito | ,000 | ,000 | ,000 | ,000 | ,000 | ,000 | ,000 | ,000 | ,000 |
| Math\_DT | ,052 | ,000 | ,000 | ,000 | ,000 | ,000 | ,000 | ,000 | ,000 |
| Task\_4 | ,537 | ,012 | ,087 | -,001 | ,000 | ,000 | ,000 | ,000 | ,000 |
| Task\_3 | 1,689 | ,037 | ,275 | -,002 | -,001 | ,000 | ,000 | ,000 | ,000 |
| Task\_1 | ,549 | ,012 | ,089 | -,001 | ,000 | ,000 | ,000 | ,000 | ,000 |
| URC\_Org\_4 | ,073 | ,002 | ,012 | ,000 | ,000 | ,132 | ,000 | ,000 | ,000 |
| URC\_Flex\_4 | ,278 | ,006 | ,045 | ,000 | ,000 | ,507 | ,000 | ,000 | ,000 |
| URC\_Flu\_4 | ,537 | ,012 | ,087 | -,001 | ,000 | ,978 | ,000 | ,000 | ,000 |
| URC\_Org\_3 | ,074 | ,002 | ,012 | ,000 | ,000 | ,135 | ,000 | ,000 | ,000 |
| URC\_Flex\_3 | ,274 | ,006 | ,045 | ,000 | ,000 | ,499 | ,000 | ,000 | ,000 |
| URC\_Flu\_3 | 1,689 | ,037 | ,275 | -,002 | -,001 | 3,076 | ,000 | ,000 | ,000 |
| URC\_Org\_1 | ,095 | ,002 | ,015 | ,000 | ,000 | ,173 | ,000 | ,000 | ,000 |
| URC\_Flex\_1 | ,230 | ,005 | ,037 | ,000 | ,000 | ,419 | ,000 | ,000 | ,000 |
| URC\_Flu\_1 | ,549 | ,012 | ,089 | -,001 | ,000 | 1,000 | ,000 | ,000 | ,000 |

##### Standardized Indirect Effects (Group number 1 - Default model)

|  | UpdatingCombined | Creativity\_TCTDP | MathKnowledge\_Cito | InhibitionIncongruentRT | MeanRTShift | Math\_DT | Task\_4 | Task\_3 | Task\_1 |
| --- | --- | --- | --- | --- | --- | --- | --- | --- | --- |
| Creativity\_TCTDP | ,000 | ,000 | ,000 | ,000 | ,000 | ,000 | ,000 | ,000 | ,000 |
| MathKnowledge\_Cito | ,000 | ,000 | ,000 | ,000 | ,000 | ,000 | ,000 | ,000 | ,000 |
| Math\_DT | ,049 | ,000 | ,000 | ,000 | ,000 | ,000 | ,000 | ,000 | ,000 |
| Task\_4 | ,287 | ,073 | ,056 | -,067 | -,035 | ,000 | ,000 | ,000 | ,000 |
| Task\_3 | ,354 | ,090 | ,069 | -,082 | -,043 | ,000 | ,000 | ,000 | ,000 |
| Task\_1 | ,334 | ,084 | ,065 | -,077 | -,040 | ,000 | ,000 | ,000 | ,000 |
| URC\_Org\_4 | ,207 | ,052 | ,040 | -,048 | -,025 | ,396 | ,000 | ,000 | ,000 |
| URC\_Flex\_4 | ,233 | ,059 | ,045 | -,054 | -,028 | ,444 | ,000 | ,000 | ,000 |
| URC\_Flu\_4 | ,284 | ,072 | ,055 | -,066 | -,034 | ,541 | ,000 | ,000 | ,000 |
| URC\_Org\_3 | ,267 | ,068 | ,052 | -,062 | -,032 | ,511 | ,000 | ,000 | ,000 |
| URC\_Flex\_3 | ,267 | ,067 | ,052 | -,062 | -,032 | ,510 | ,000 | ,000 | ,000 |
| URC\_Flu\_3 | ,163 | ,041 | ,032 | -,038 | -,020 | ,311 | ,000 | ,000 | ,000 |
| URC\_Org\_1 | ,234 | ,059 | ,046 | -,054 | -,028 | ,446 | ,000 | ,000 | ,000 |
| URC\_Flex\_1 | ,254 | ,064 | ,050 | -,059 | -,031 | ,485 | ,000 | ,000 | ,000 |
| URC\_Flu\_1 | ,317 | ,080 | ,062 | -,074 | -,038 | ,606 | ,000 | ,000 | ,000 |

##### Modification Indices (Group number 1 - Default model)

##### Covariances: (Group number 1 - Default model)

|  |  |  | M.I. | Par Change |
| --- | --- | --- | --- | --- |
| e9 | <--> | e2 | 4,073 | ,740 |
| e7 | <--> | e3 | 4,135 | -8,326 |

##### Variances: (Group number 1 - Default model)

|  |  |  | M.I. | Par Change |
| --- | --- | --- | --- | --- |

##### Regression Weights: (Group number 1 - Default model)

|  |  |  | M.I. | Par Change |
| --- | --- | --- | --- | --- |
| URC\_Flu\_3 | <--- | UpdatingCombined | 7,984 | 1,611 |

##### Means: (Group number 1 - Default model)

|  |  |  | M.I. | Par Change |
| --- | --- | --- | --- | --- |

##### Intercepts: (Group number 1 - Default model)

|  |  |  | M.I. | Par Change |
| --- | --- | --- | --- | --- |

##### Bootstrap (Group number 1 - Default model)

##### Bootstrap standard errors (Group number 1 - Default model)

##### Scalar Estimates (Group number 1 - Default model)

##### Regression Weights: (Group number 1 - Default model)

| Parameter | | | SE | SE-SE | Mean | Bias | SE-Bias |
| --- | --- | --- | --- | --- | --- | --- | --- |
| Creativity\_TCTDP | <--- | UpdatingCombined | ,491 | ,035 |
| MathKnowledge\_Cito | <--- | UpdatingCombined | ,111 | ,008 |
| Math\_DT | <--- | MathKnowledge\_Cito | ,055 | ,004 |
| Math\_DT | <--- | Creativity\_TCTDP | ,007 | ,001 |
| Math\_DT | <--- | InhibitionIncongruentRT | ,000 | ,000 |
| Math\_DT | <--- | MeanRTShift | ,000 | ,000 |
| Math\_DT | <--- | UpdatingCombined | ,096 | ,007 |
| Task\_1 | <--- | Math\_DT | ,000 | ,000 |
| Task\_3 | <--- | Math\_DT | ,567 | ,040 |
| Task\_4 | <--- | Math\_DT | ,161 | ,011 |
| URC\_Flu\_1 | <--- | Task\_1 | ,000 | ,000 |
| URC\_Flex\_1 | <--- | Task\_1 | ,036 | ,003 |
| URC\_Org\_1 | <--- | Task\_1 | ,011 | ,001 |
| URC\_Flu\_3 | <--- | Task\_3 | ,000 | ,000 |
| URC\_Flex\_3 | <--- | Task\_3 | ,023 | ,002 |
| URC\_Org\_3 | <--- | Task\_3 | ,006 | ,000 |
| URC\_Flu\_4 | <--- | Task\_4 | ,000 | ,000 |
| URC\_Flex\_4 | <--- | Task\_4 | ,043 | ,003 |
| URC\_Org\_4 | <--- | Task\_4 | ,010 | ,001 |

##### Standardized Regression Weights: (Group number 1 - Default model)

| Parameter | | | SE | SE-SE | Mean | Bias | SE-Bias |
| --- | --- | --- | --- | --- | --- | --- | --- |
| Creativity\_TCTDP | <--- | UpdatingCombined | ,040 | ,003 |
| MathKnowledge\_Cito | <--- | UpdatingCombined | ,083 | ,006 |
| Math\_DT | <--- | MathKnowledge\_Cito | ,059 | ,004 |
| Math\_DT | <--- | Creativity\_TCTDP | ,075 | ,005 |
| Math\_DT | <--- | InhibitionIncongruentRT | ,075 | ,005 |
| Math\_DT | <--- | MeanRTShift | ,078 | ,005 |
| Math\_DT | <--- | UpdatingCombined | ,072 | ,005 |
| Task\_1 | <--- | Math\_DT | ,057 | ,004 |
| Task\_3 | <--- | Math\_DT | ,058 | ,004 |
| Task\_4 | <--- | Math\_DT | ,062 | ,004 |
| URC\_Flu\_1 | <--- | Task\_1 | ,026 | ,002 |
| URC\_Flex\_1 | <--- | Task\_1 | ,034 | ,002 |
| URC\_Org\_1 | <--- | Task\_1 | ,036 | ,003 |
| URC\_Flu\_3 | <--- | Task\_3 | ,059 | ,004 |
| URC\_Flex\_3 | <--- | Task\_3 | ,044 | ,003 |
| URC\_Org\_3 | <--- | Task\_3 | ,047 | ,003 |
| URC\_Flu\_4 | <--- | Task\_4 | ,021 | ,001 |
| URC\_Flex\_4 | <--- | Task\_4 | ,036 | ,003 |
| URC\_Org\_4 | <--- | Task\_4 | ,033 | ,002 |

##### Intercepts: (Group number 1 - Default model)

| Parameter | | | SE | SE-SE | Mean | Bias | SE-Bias |
| --- | --- | --- | --- | --- | --- | --- | --- |
| UpdatingCombined |  |  | ,053 | ,004 |
| MeanRTShift |  |  | 15,090 | 1,067 |
| InhibitionIncongruentRT |  |  | 9,481 | ,670 |
| Creativity\_TCTDP |  |  | ,567 | ,040 |
| MathKnowledge\_Cito |  |  | ,058 | ,004 |
| URC\_Flu\_1 |  |  | ,425 | ,030 |
| URC\_Flex\_1 |  |  | ,178 | ,013 |
| URC\_Org\_1 |  |  | ,073 | ,005 |
| URC\_Flu\_3 |  |  | 1,381 | ,098 |
| URC\_Flex\_3 |  |  | ,201 | ,014 |
| URC\_Org\_3 |  |  | ,056 | ,004 |
| URC\_Flu\_4 |  |  | ,431 | ,030 |
| URC\_Flex\_4 |  |  | ,229 | ,016 |
| URC\_Org\_4 |  |  | ,058 | ,004 |

##### Covariances: (Group number 1 - Default model)

| Parameter | | | SE | SE-SE | Mean | Bias | SE-Bias |
| --- | --- | --- | --- | --- | --- | --- | --- |
| e1 | <--> | e3 | 2384,716 | 168,625 |
| e3 | <--> | e2 | 8,339 | ,590 |
| e1 | <--> | e2 | 12,613 | ,892 |
| e6 | <--> | e9 | ,416 | ,029 |
| e6 | <--> | e12 | ,063 | ,004 |
| e9 | <--> | e12 | ,392 | ,028 |
| e7 | <--> | e10 | ,021 | ,001 |
| e7 | <--> | e13 | ,018 | ,001 |
| e10 | <--> | e13 | ,021 | ,001 |
| e8 | <--> | e11 | ,003 | ,000 |
| e8 | <--> | e14 | ,003 | ,000 |
| e11 | <--> | e14 | ,002 | ,000 |

##### Correlations: (Group number 1 - Default model)

| Parameter | | | SE | SE-SE | Mean | Bias | SE-Bias |
| --- | --- | --- | --- | --- | --- | --- | --- |
| e1 | <--> | e3 | ,058 | ,004 |
| e3 | <--> | e2 | ,049 | ,003 |
| e1 | <--> | e2 | ,064 | ,004 |
| e6 | <--> | e9 | ,126 | ,009 |
| e6 | <--> | e12 | ,806 | ,057 |
| e9 | <--> | e12 | ,221 | ,016 |
| e7 | <--> | e10 | ,072 | ,005 |
| e7 | <--> | e13 | ,059 | ,004 |
| e10 | <--> | e13 | ,065 | ,005 |
| e8 | <--> | e11 | ,074 | ,005 |
| e8 | <--> | e14 | ,063 | ,004 |
| e11 | <--> | e14 | ,072 | ,005 |

##### Variances: (Group number 1 - Default model)

| Parameter | | | SE | SE-SE | Mean | Bias | SE-Bias |
| --- | --- | --- | --- | --- | --- | --- | --- |
| e2 |  |  | ,067 | ,005 |
| e1 |  |  | 6243,358 | 441,472 |
| e3 |  |  | 2573,795 | 181,995 |
| e5 |  |  | ,128 | ,009 |
| e4 |  |  | 6,173 | ,437 |
| e15 |  |  | ,120 | ,008 |
| e16 |  |  | ,157 | ,011 |
| e17 |  |  | 2,071 | ,146 |
| e18 |  |  | ,233 | ,016 |
| e6 |  |  | ,109 | ,008 |
| e8 |  |  | ,006 | ,000 |
| e7 |  |  | ,027 | ,002 |
| e9 |  |  | 14,788 | 1,046 |
| e10 |  |  | ,042 | ,003 |
| e11 |  |  | ,004 | ,000 |
| e13 |  |  | ,052 | ,004 |
| e12 |  |  | ,107 | ,008 |
| e14 |  |  | ,003 | ,000 |

##### Squared Multiple Correlations: (Group number 1 - Default model)

| Parameter | | | SE | SE-SE | Mean | Bias | SE-Bias |
| --- | --- | --- | --- | --- | --- | --- | --- |
| UpdatingCombined |  |  | ,000 | ,000 |
| Creativity\_TCTDP |  |  | ,018 | ,001 |
| MathKnowledge\_Cito |  |  | ,032 | ,002 |
| InhibitionIncongruentRT |  |  | ,000 | ,000 |
| MeanRTShift |  |  | ,000 | ,000 |
| Math\_DT |  |  | ,068 | ,005 |
| Task\_4 |  |  | ,068 | ,005 |
| Task\_3 |  |  | ,078 | ,005 |
| Task\_1 |  |  | ,073 | ,005 |
| URC\_Org\_4 |  |  | ,047 | ,003 |
| URC\_Flex\_4 |  |  | ,058 | ,004 |
| URC\_Flu\_4 |  |  | ,042 | ,003 |
| URC\_Org\_3 |  |  | ,071 | ,005 |
| URC\_Flex\_3 |  |  | ,066 | ,005 |
| URC\_Flu\_3 |  |  | ,054 | ,004 |
| URC\_Org\_1 |  |  | ,050 | ,004 |
| URC\_Flex\_1 |  |  | ,052 | ,004 |
| URC\_Flu\_1 |  |  | ,049 | ,003 |

##### Matrices (Group number 1 - Default model)

##### Implied Covariances - Standard Errors (Group number 1 - Default model)

|  | UpdatingCombined | Creativity\_TCTDP | MathKnowledge\_Cito | InhibitionIncongruentRT | MeanRTShift | URC\_Org\_4 | URC\_Flex\_4 | URC\_Flu\_4 | URC\_Org\_3 | URC\_Flex\_3 | URC\_Flu\_3 | URC\_Org\_1 | URC\_Flex\_1 | URC\_Flu\_1 |
| --- | --- | --- | --- | --- | --- | --- | --- | --- | --- | --- | --- | --- | --- | --- |
| UpdatingCombined | ,067 |
| Creativity\_TCTDP | ,341 | 6,287 |
| MathKnowledge\_Cito | ,082 | ,225 | ,156 |
| InhibitionIncongruentRT | 8,339 | 26,250 | 5,219 | 2573,795 |
| MeanRTShift | 12,613 | 34,814 | 3,216 | 2384,716 | 6243,358 |
| URC\_Org\_4 | ,010 | ,095 | ,010 | 1,581 | 2,080 | ,006 |
| URC\_Flex\_4 | ,038 | ,350 | ,039 | 5,818 | 7,993 | ,021 | ,083 |
| URC\_Flu\_4 | ,068 | ,664 | ,076 | 11,141 | 15,158 | ,034 | ,126 | ,311 |
| URC\_Org\_3 | ,009 | ,093 | ,009 | 1,428 | 1,985 | ,003 | ,009 | ,016 | ,005 |
| URC\_Flex\_3 | ,035 | ,327 | ,035 | 5,047 | 7,541 | ,010 | ,044 | ,065 | ,018 | ,068 |
| URC\_Flu\_3 | ,257 | 2,251 | ,227 | 36,003 | 46,726 | ,064 | ,240 | ,501 | ,096 | ,368 | 15,430 |
| URC\_Org\_1 | ,012 | ,124 | ,013 | 1,773 | 2,583 | ,004 | ,012 | ,022 | ,003 | ,010 | ,076 | ,005 |
| URC\_Flex\_1 | ,030 | ,304 | ,032 | 4,409 | 6,042 | ,008 | ,033 | ,056 | ,006 | ,033 | ,177 | ,015 | ,040 |
| URC\_Flu\_1 | ,064 | ,694 | ,074 | 9,363 | 15,204 | ,020 | ,073 | ,136 | ,014 | ,056 | ,585 | ,029 | ,065 | ,208 |

##### Implied Correlations - Standard Errors (Group number 1 - Default model)

|  | UpdatingCombined | Creativity\_TCTDP | MathKnowledge\_Cito | InhibitionIncongruentRT | MeanRTShift | URC\_Org\_4 | URC\_Flex\_4 | URC\_Flu\_4 | URC\_Org\_3 | URC\_Flex\_3 | URC\_Flu\_3 | URC\_Org\_1 | URC\_Flex\_1 | URC\_Flu\_1 |
| --- | --- | --- | --- | --- | --- | --- | --- | --- | --- | --- | --- | --- | --- | --- |
| UpdatingCombined | ,000 |
| Creativity\_TCTDP | ,040 | ,000 |
| MathKnowledge\_Cito | ,083 | ,021 | ,000 |
| InhibitionIncongruentRT | ,049 | ,016 | ,030 | ,000 |
| MeanRTShift | ,064 | ,015 | ,013 | ,058 | ,000 |
| URC\_Org\_4 | ,033 | ,033 | ,032 | ,033 | ,031 | ,000 |
| URC\_Flex\_4 | ,035 | ,035 | ,035 | ,035 | ,034 | ,040 | ,000 |
| URC\_Flu\_4 | ,037 | ,042 | ,042 | ,040 | ,041 | ,027 | ,038 | ,000 |
| URC\_Org\_3 | ,038 | ,041 | ,037 | ,037 | ,037 | ,043 | ,034 | ,037 | ,000 |
| URC\_Flex\_3 | ,038 | ,040 | ,037 | ,035 | ,038 | ,034 | ,046 | ,039 | ,055 | ,000 |
| URC\_Flu\_3 | ,035 | ,027 | ,026 | ,027 | ,023 | ,026 | ,029 | ,042 | ,048 | ,050 | ,000 |
| URC\_Org\_1 | ,039 | ,038 | ,034 | ,031 | ,033 | ,040 | ,032 | ,036 | ,039 | ,030 | ,026 | ,000 |
| URC\_Flex\_1 | ,041 | ,040 | ,038 | ,034 | ,035 | ,034 | ,037 | ,039 | ,029 | ,046 | ,027 | ,041 | ,000 |
| URC\_Flu\_1 | ,043 | ,048 | ,046 | ,037 | ,044 | ,041 | ,042 | ,052 | ,032 | ,034 | ,051 | ,034 | ,032 | ,000 |

##### Implied Means - Standard Errors (Group number 1 - Default model)

|  | UpdatingCombined | Creativity\_TCTDP | MathKnowledge\_Cito | InhibitionIncongruentRT | MeanRTShift | URC\_Org\_4 | URC\_Flex\_4 | URC\_Flu\_4 | URC\_Org\_3 | URC\_Flex\_3 | URC\_Flu\_3 | URC\_Org\_1 | URC\_Flex\_1 | URC\_Flu\_1 |
| --- | --- | --- | --- | --- | --- | --- | --- | --- | --- | --- | --- | --- | --- | --- |
| UpdatingCombined | ,053 | ,603 | ,060 | 9,481 | 15,090 | ,015 | ,062 | ,095 | ,015 | ,051 | ,600 | ,019 | ,045 | ,090 |

##### Sample Covariances - Standard Errors (Group number 1 - Default model)

|  | UpdatingCombined | Creativity\_TCTDP | MathKnowledge\_Cito | InhibitionIncongruentRT | MeanRTShift | URC\_Org\_4 | URC\_Flex\_4 | URC\_Flu\_4 | URC\_Org\_3 | URC\_Flex\_3 | URC\_Flu\_3 | URC\_Org\_1 | URC\_Flex\_1 | URC\_Flu\_1 |
| --- | --- | --- | --- | --- | --- | --- | --- | --- | --- | --- | --- | --- | --- | --- |
| UpdatingCombined | ,067 |
| Creativity\_TCTDP | ,341 | 6,287 |
| MathKnowledge\_Cito | ,082 | ,644 | ,156 |
| InhibitionIncongruentRT | 8,339 | 75,193 | 7,338 | 2573,795 |
| MeanRTShift | 12,613 | 129,555 | 12,378 | 2384,716 | 6243,358 |
| URC\_Org\_4 | ,015 | ,182 | ,018 | 2,497 | 4,942 | ,006 |
| URC\_Flex\_4 | ,053 | ,626 | ,067 | 9,252 | 15,708 | ,021 | ,083 |
| URC\_Flu\_4 | ,073 | ,941 | ,104 | 12,094 | 20,747 | ,034 | ,125 | ,306 |
| URC\_Org\_3 | ,011 | ,151 | ,015 | 1,814 | 3,490 | ,003 | ,014 | ,019 | ,005 |
| URC\_Flex\_3 | ,044 | ,524 | ,060 | 7,470 | 11,834 | ,014 | ,050 | ,073 | ,017 | ,069 |
| URC\_Flu\_3 | ,416 | 4,369 | ,526 | 72,724 | 107,933 | ,153 | ,437 | ,658 | ,110 | ,399 | 15,510 |
| URC\_Org\_1 | ,015 | ,225 | ,018 | 2,872 | 5,116 | ,006 | ,022 | ,035 | ,004 | ,016 | ,167 | ,005 |
| URC\_Flex\_1 | ,036 | ,431 | ,036 | 6,307 | 10,647 | ,012 | ,044 | ,067 | ,009 | ,037 | ,411 | ,016 | ,040 |
| URC\_Flu\_1 | ,070 | ,931 | ,070 | 11,506 | 21,811 | ,024 | ,091 | ,137 | ,019 | ,074 | ,739 | ,029 | ,064 | ,209 |

##### Sample Correlations - Standard Errors (Group number 1 - Default model)

|  | UpdatingCombined | Creativity\_TCTDP | MathKnowledge\_Cito | InhibitionIncongruentRT | MeanRTShift | URC\_Org\_4 | URC\_Flex\_4 | URC\_Flu\_4 | URC\_Org\_3 | URC\_Flex\_3 | URC\_Flu\_3 | URC\_Org\_1 | URC\_Flex\_1 | URC\_Flu\_1 |
| --- | --- | --- | --- | --- | --- | --- | --- | --- | --- | --- | --- | --- | --- | --- |
| UpdatingCombined | ,000 |
| Creativity\_TCTDP | ,040 | ,000 |
| MathKnowledge\_Cito | ,083 | ,067 | ,000 |
| InhibitionIncongruentRT | ,049 | ,052 | ,047 | ,000 |
| MeanRTShift | ,064 | ,057 | ,054 | ,058 | ,000 |
| URC\_Org\_4 | ,059 | ,063 | ,059 | ,056 | ,072 | ,000 |
| URC\_Flex\_4 | ,058 | ,064 | ,061 | ,059 | ,067 | ,042 | ,000 |
| URC\_Flu\_4 | ,045 | ,061 | ,062 | ,046 | ,056 | ,028 | ,039 | ,000 |
| URC\_Org\_3 | ,047 | ,070 | ,062 | ,046 | ,065 | ,047 | ,058 | ,047 | ,000 |
| URC\_Flex\_3 | ,054 | ,065 | ,064 | ,055 | ,059 | ,056 | ,052 | ,048 | ,048 | ,000 |
| URC\_Flu\_3 | ,036 | ,056 | ,060 | ,037 | ,054 | ,063 | ,056 | ,055 | ,059 | ,054 | ,000 |
| URC\_Org\_1 | ,053 | ,070 | ,052 | ,056 | ,065 | ,062 | ,064 | ,058 | ,052 | ,053 | ,048 | ,000 |
| URC\_Flex\_1 | ,051 | ,059 | ,044 | ,050 | ,059 | ,053 | ,054 | ,051 | ,046 | ,054 | ,049 | ,047 | ,000 |
| URC\_Flu\_1 | ,048 | ,065 | ,045 | ,051 | ,062 | ,053 | ,055 | ,051 | ,050 | ,054 | ,053 | ,034 | ,032 | ,000 |

##### Sample Means - Standard Errors (Group number 1 - Default model)

|  | UpdatingCombined | Creativity\_TCTDP | MathKnowledge\_Cito | InhibitionIncongruentRT | MeanRTShift | URC\_Org\_4 | URC\_Flex\_4 | URC\_Flu\_4 | URC\_Org\_3 | URC\_Flex\_3 | URC\_Flu\_3 | URC\_Org\_1 | URC\_Flex\_1 | URC\_Flu\_1 |
| --- | --- | --- | --- | --- | --- | --- | --- | --- | --- | --- | --- | --- | --- | --- |
| UpdatingCombined | ,053 | ,603 | ,060 | 9,481 | 15,090 | ,015 | ,062 | ,095 | ,015 | ,051 | ,600 | ,019 | ,045 | ,090 |

##### Total Effects - Standard Errors (Group number 1 - Default model)

|  | UpdatingCombined | Creativity\_TCTDP | MathKnowledge\_Cito | InhibitionIncongruentRT | MeanRTShift | Math\_DT | Task\_4 | Task\_3 | Task\_1 |
| --- | --- | --- | --- | --- | --- | --- | --- | --- | --- |
| Creativity\_TCTDP | ,491 | ,000 | ,000 | ,000 | ,000 | ,000 | ,000 | ,000 | ,000 |
| MathKnowledge\_Cito | ,111 | ,000 | ,000 | ,000 | ,000 | ,000 | ,000 | ,000 | ,000 |
| Math\_DT | ,094 | ,007 | ,055 | ,000 | ,000 | ,000 | ,000 | ,000 | ,000 |
| Task\_4 | ,082 | ,007 | ,054 | ,000 | ,000 | ,161 | ,000 | ,000 | ,000 |
| Task\_3 | ,337 | ,022 | ,165 | ,001 | ,001 | ,567 | ,000 | ,000 | ,000 |
| Task\_1 | ,094 | ,007 | ,055 | ,000 | ,000 | ,000 | ,000 | ,000 | ,000 |
| URC\_Org\_4 | ,011 | ,001 | ,007 | ,000 | ,000 | ,024 | ,010 | ,000 | ,000 |
| URC\_Flex\_4 | ,044 | ,004 | ,028 | ,000 | ,000 | ,084 | ,043 | ,000 | ,000 |
| URC\_Flu\_4 | ,082 | ,007 | ,054 | ,000 | ,000 | ,161 | ,000 | ,000 | ,000 |
| URC\_Org\_3 | ,012 | ,001 | ,007 | ,000 | ,000 | ,022 | ,000 | ,006 | ,000 |
| URC\_Flex\_3 | ,045 | ,003 | ,027 | ,000 | ,000 | ,079 | ,000 | ,023 | ,000 |
| URC\_Flu\_3 | ,337 | ,022 | ,165 | ,001 | ,001 | ,567 | ,000 | ,000 | ,000 |
| URC\_Org\_1 | ,017 | ,001 | ,009 | ,000 | ,000 | ,011 | ,000 | ,000 | ,011 |
| URC\_Flex\_1 | ,042 | ,003 | ,023 | ,000 | ,000 | ,036 | ,000 | ,000 | ,036 |
| URC\_Flu\_1 | ,094 | ,007 | ,055 | ,000 | ,000 | ,000 | ,000 | ,000 | ,000 |

##### Standardized Total Effects - Standard Errors (Group number 1 - Default model)

|  | UpdatingCombined | Creativity\_TCTDP | MathKnowledge\_Cito | InhibitionIncongruentRT | MeanRTShift | Math\_DT | Task\_4 | Task\_3 | Task\_1 |
| --- | --- | --- | --- | --- | --- | --- | --- | --- | --- |
| Creativity\_TCTDP | ,040 | ,000 | ,000 | ,000 | ,000 | ,000 | ,000 | ,000 | ,000 |
| MathKnowledge\_Cito | ,083 | ,000 | ,000 | ,000 | ,000 | ,000 | ,000 | ,000 | ,000 |
| Math\_DT | ,063 | ,075 | ,059 | ,075 | ,078 | ,000 | ,000 | ,000 | ,000 |
| Task\_4 | ,039 | ,042 | ,034 | ,043 | ,043 | ,062 | ,000 | ,000 | ,000 |
| Task\_3 | ,054 | ,052 | ,040 | ,051 | ,053 | ,058 | ,000 | ,000 | ,000 |
| Task\_1 | ,052 | ,049 | ,038 | ,047 | ,050 | ,057 | ,000 | ,000 | ,000 |
| URC\_Org\_4 | ,032 | ,031 | ,025 | ,031 | ,031 | ,054 | ,033 | ,000 | ,000 |
| URC\_Flex\_4 | ,034 | ,034 | ,027 | ,034 | ,035 | ,054 | ,036 | ,000 | ,000 |
| URC\_Flu\_4 | ,038 | ,041 | ,033 | ,042 | ,042 | ,057 | ,021 | ,000 | ,000 |
| URC\_Org\_3 | ,039 | ,039 | ,030 | ,039 | ,039 | ,043 | ,000 | ,047 | ,000 |
| URC\_Flex\_3 | ,041 | ,039 | ,030 | ,037 | ,040 | ,043 | ,000 | ,044 | ,000 |
| URC\_Flu\_3 | ,034 | ,024 | ,019 | ,024 | ,024 | ,052 | ,000 | ,059 | ,000 |
| URC\_Org\_1 | ,041 | ,035 | ,027 | ,033 | ,035 | ,048 | ,000 | ,000 | ,036 |
| URC\_Flex\_1 | ,042 | ,038 | ,029 | ,037 | ,038 | ,051 | ,000 | ,000 | ,034 |
| URC\_Flu\_1 | ,048 | ,046 | ,037 | ,044 | ,047 | ,053 | ,000 | ,000 | ,026 |

##### Direct Effects - Standard Errors (Group number 1 - Default model)

|  | UpdatingCombined | Creativity\_TCTDP | MathKnowledge\_Cito | InhibitionIncongruentRT | MeanRTShift | Math\_DT | Task\_4 | Task\_3 | Task\_1 |
| --- | --- | --- | --- | --- | --- | --- | --- | --- | --- |
| Creativity\_TCTDP | ,491 | ,000 | ,000 | ,000 | ,000 | ,000 | ,000 | ,000 | ,000 |
| MathKnowledge\_Cito | ,111 | ,000 | ,000 | ,000 | ,000 | ,000 | ,000 | ,000 | ,000 |
| Math\_DT | ,096 | ,007 | ,055 | ,000 | ,000 | ,000 | ,000 | ,000 | ,000 |
| Task\_4 | ,000 | ,000 | ,000 | ,000 | ,000 | ,161 | ,000 | ,000 | ,000 |
| Task\_3 | ,000 | ,000 | ,000 | ,000 | ,000 | ,567 | ,000 | ,000 | ,000 |
| Task\_1 | ,000 | ,000 | ,000 | ,000 | ,000 | ,000 | ,000 | ,000 | ,000 |
| URC\_Org\_4 | ,000 | ,000 | ,000 | ,000 | ,000 | ,000 | ,010 | ,000 | ,000 |
| URC\_Flex\_4 | ,000 | ,000 | ,000 | ,000 | ,000 | ,000 | ,043 | ,000 | ,000 |
| URC\_Flu\_4 | ,000 | ,000 | ,000 | ,000 | ,000 | ,000 | ,000 | ,000 | ,000 |
| URC\_Org\_3 | ,000 | ,000 | ,000 | ,000 | ,000 | ,000 | ,000 | ,006 | ,000 |
| URC\_Flex\_3 | ,000 | ,000 | ,000 | ,000 | ,000 | ,000 | ,000 | ,023 | ,000 |
| URC\_Flu\_3 | ,000 | ,000 | ,000 | ,000 | ,000 | ,000 | ,000 | ,000 | ,000 |
| URC\_Org\_1 | ,000 | ,000 | ,000 | ,000 | ,000 | ,000 | ,000 | ,000 | ,011 |
| URC\_Flex\_1 | ,000 | ,000 | ,000 | ,000 | ,000 | ,000 | ,000 | ,000 | ,036 |
| URC\_Flu\_1 | ,000 | ,000 | ,000 | ,000 | ,000 | ,000 | ,000 | ,000 | ,000 |

##### Standardized Direct Effects - Standard Errors (Group number 1 - Default model)

|  | UpdatingCombined | Creativity\_TCTDP | MathKnowledge\_Cito | InhibitionIncongruentRT | MeanRTShift | Math\_DT | Task\_4 | Task\_3 | Task\_1 |
| --- | --- | --- | --- | --- | --- | --- | --- | --- | --- |
| Creativity\_TCTDP | ,040 | ,000 | ,000 | ,000 | ,000 | ,000 | ,000 | ,000 | ,000 |
| MathKnowledge\_Cito | ,083 | ,000 | ,000 | ,000 | ,000 | ,000 | ,000 | ,000 | ,000 |
| Math\_DT | ,072 | ,075 | ,059 | ,075 | ,078 | ,000 | ,000 | ,000 | ,000 |
| Task\_4 | ,000 | ,000 | ,000 | ,000 | ,000 | ,062 | ,000 | ,000 | ,000 |
| Task\_3 | ,000 | ,000 | ,000 | ,000 | ,000 | ,058 | ,000 | ,000 | ,000 |
| Task\_1 | ,000 | ,000 | ,000 | ,000 | ,000 | ,057 | ,000 | ,000 | ,000 |
| URC\_Org\_4 | ,000 | ,000 | ,000 | ,000 | ,000 | ,000 | ,033 | ,000 | ,000 |
| URC\_Flex\_4 | ,000 | ,000 | ,000 | ,000 | ,000 | ,000 | ,036 | ,000 | ,000 |
| URC\_Flu\_4 | ,000 | ,000 | ,000 | ,000 | ,000 | ,000 | ,021 | ,000 | ,000 |
| URC\_Org\_3 | ,000 | ,000 | ,000 | ,000 | ,000 | ,000 | ,000 | ,047 | ,000 |
| URC\_Flex\_3 | ,000 | ,000 | ,000 | ,000 | ,000 | ,000 | ,000 | ,044 | ,000 |
| URC\_Flu\_3 | ,000 | ,000 | ,000 | ,000 | ,000 | ,000 | ,000 | ,059 | ,000 |
| URC\_Org\_1 | ,000 | ,000 | ,000 | ,000 | ,000 | ,000 | ,000 | ,000 | ,036 |
| URC\_Flex\_1 | ,000 | ,000 | ,000 | ,000 | ,000 | ,000 | ,000 | ,000 | ,034 |
| URC\_Flu\_1 | ,000 | ,000 | ,000 | ,000 | ,000 | ,000 | ,000 | ,000 | ,026 |

##### Indirect Effects - Standard Errors (Group number 1 - Default model)

|  | UpdatingCombined | Creativity\_TCTDP | MathKnowledge\_Cito | InhibitionIncongruentRT | MeanRTShift | Math\_DT | Task\_4 | Task\_3 | Task\_1 |
| --- | --- | --- | --- | --- | --- | --- | --- | --- | --- |
| Creativity\_TCTDP | ,000 | ,000 | ,000 | ,000 | ,000 | ,000 | ,000 | ,000 | ,000 |
| MathKnowledge\_Cito | ,000 | ,000 | ,000 | ,000 | ,000 | ,000 | ,000 | ,000 | ,000 |
| Math\_DT | ,027 | ,000 | ,000 | ,000 | ,000 | ,000 | ,000 | ,000 | ,000 |
| Task\_4 | ,082 | ,007 | ,054 | ,000 | ,000 | ,000 | ,000 | ,000 | ,000 |
| Task\_3 | ,337 | ,022 | ,165 | ,001 | ,001 | ,000 | ,000 | ,000 | ,000 |
| Task\_1 | ,094 | ,007 | ,055 | ,000 | ,000 | ,000 | ,000 | ,000 | ,000 |
| URC\_Org\_4 | ,011 | ,001 | ,007 | ,000 | ,000 | ,024 | ,000 | ,000 | ,000 |
| URC\_Flex\_4 | ,044 | ,004 | ,028 | ,000 | ,000 | ,084 | ,000 | ,000 | ,000 |
| URC\_Flu\_4 | ,082 | ,007 | ,054 | ,000 | ,000 | ,161 | ,000 | ,000 | ,000 |
| URC\_Org\_3 | ,012 | ,001 | ,007 | ,000 | ,000 | ,022 | ,000 | ,000 | ,000 |
| URC\_Flex\_3 | ,045 | ,003 | ,027 | ,000 | ,000 | ,079 | ,000 | ,000 | ,000 |
| URC\_Flu\_3 | ,337 | ,022 | ,165 | ,001 | ,001 | ,567 | ,000 | ,000 | ,000 |
| URC\_Org\_1 | ,017 | ,001 | ,009 | ,000 | ,000 | ,011 | ,000 | ,000 | ,000 |
| URC\_Flex\_1 | ,042 | ,003 | ,023 | ,000 | ,000 | ,036 | ,000 | ,000 | ,000 |
| URC\_Flu\_1 | ,094 | ,007 | ,055 | ,000 | ,000 | ,000 | ,000 | ,000 | ,000 |

##### Standardized Indirect Effects - Standard Errors (Group number 1 - Default model)

|  | UpdatingCombined | Creativity\_TCTDP | MathKnowledge\_Cito | InhibitionIncongruentRT | MeanRTShift | Math\_DT | Task\_4 | Task\_3 | Task\_1 |
| --- | --- | --- | --- | --- | --- | --- | --- | --- | --- |
| Creativity\_TCTDP | ,000 | ,000 | ,000 | ,000 | ,000 | ,000 | ,000 | ,000 | ,000 |
| MathKnowledge\_Cito | ,000 | ,000 | ,000 | ,000 | ,000 | ,000 | ,000 | ,000 | ,000 |
| Math\_DT | ,025 | ,000 | ,000 | ,000 | ,000 | ,000 | ,000 | ,000 | ,000 |
| Task\_4 | ,039 | ,042 | ,034 | ,043 | ,043 | ,000 | ,000 | ,000 | ,000 |
| Task\_3 | ,054 | ,052 | ,040 | ,051 | ,053 | ,000 | ,000 | ,000 | ,000 |
| Task\_1 | ,052 | ,049 | ,038 | ,047 | ,050 | ,000 | ,000 | ,000 | ,000 |
| URC\_Org\_4 | ,032 | ,031 | ,025 | ,031 | ,031 | ,054 | ,000 | ,000 | ,000 |
| URC\_Flex\_4 | ,034 | ,034 | ,027 | ,034 | ,035 | ,054 | ,000 | ,000 | ,000 |
| URC\_Flu\_4 | ,038 | ,041 | ,033 | ,042 | ,042 | ,057 | ,000 | ,000 | ,000 |
| URC\_Org\_3 | ,039 | ,039 | ,030 | ,039 | ,039 | ,043 | ,000 | ,000 | ,000 |
| URC\_Flex\_3 | ,041 | ,039 | ,030 | ,037 | ,040 | ,043 | ,000 | ,000 | ,000 |
| URC\_Flu\_3 | ,034 | ,024 | ,019 | ,024 | ,024 | ,052 | ,000 | ,000 | ,000 |
| URC\_Org\_1 | ,041 | ,035 | ,027 | ,033 | ,035 | ,048 | ,000 | ,000 | ,000 |
| URC\_Flex\_1 | ,042 | ,038 | ,029 | ,037 | ,038 | ,051 | ,000 | ,000 | ,000 |
| URC\_Flu\_1 | ,048 | ,046 | ,037 | ,044 | ,047 | ,053 | ,000 | ,000 | ,000 |

##### Minimization History (Default model)

| Iteration |  | Negative eigenvalues | Condition # | Smallest eigenvalue | Diameter | F | NTries | Ratio |
| --- | --- | --- | --- | --- | --- | --- | --- | --- |
| 0 | e | 9 |  | -,374 | 9999,000 | 1593,049 | 0 | 9999,000 |
| 1 | e | 8 |  | -,378 | 1,599 | 769,527 | 19 | ,681 |
| 2 | e | 2 |  | -,054 | ,909 | 305,232 | 4 | ,831 |
| 3 | e | 1 |  | -,015 | ,270 | 217,114 | 5 | ,823 |
| 4 | e | 0 | 2658783,939 |  | ,879 | 130,345 | 8 | ,734 |
| 5 | e | 0 | 2944283,800 |  | 1,089 | 96,377 | 2 | ,000 |
| 6 | e | 0 | 3176427,460 |  | ,499 | 80,646 | 2 | ,000 |
| 7 | e | 0 | 3200892,138 |  | ,443 | 73,226 | 1 | 1,135 |
| 8 | e | 0 | 3278239,935 |  | ,131 | 72,656 | 1 | 1,083 |
| 9 | e | 0 | 3296420,136 |  | ,036 | 72,640 | 1 | 1,029 |
| 10 | e | 0 | 3406064,548 |  | ,002 | 72,640 | 1 | 1,002 |
| 11 | e | 0 | 3405038,512 |  | ,000 | 72,640 | 1 | 1,000 |

##### Pairwise Parameter Comparisons (Default model)

##### Variance-covariance Matrix of Estimates (Default model)

|  | par\_1 | par\_2 | par\_3 | par\_4 | par\_5 | par\_6 | par\_7 | par\_8 | par\_9 | par\_10 | par\_11 | par\_12 | par\_13 | par\_14 | par\_15 | par\_16 | par\_17 | par\_18 | par\_19 | par\_20 | par\_21 | par\_22 | par\_23 | par\_24 | par\_25 | par\_26 | par\_27 | par\_28 | par\_29 | par\_30 | par\_31 | par\_32 | par\_33 | par\_34 | par\_35 | par\_36 | par\_37 | par\_38 | par\_39 | par\_40 | par\_41 | par\_42 | par\_43 | par\_44 | par\_45 | par\_46 | par\_47 | par\_48 | par\_49 | par\_50 | par\_51 | par\_52 | par\_53 | par\_54 | par\_55 | par\_56 | par\_57 | par\_58 | par\_59 |
| --- | --- | --- | --- | --- | --- | --- | --- | --- | --- | --- | --- | --- | --- | --- | --- | --- | --- | --- | --- | --- | --- | --- | --- | --- | --- | --- | --- | --- | --- | --- | --- | --- | --- | --- | --- | --- | --- | --- | --- | --- | --- | --- | --- | --- | --- | --- | --- | --- | --- | --- | --- | --- | --- | --- | --- | --- | --- | --- | --- |
| par\_1 | ,416 |
| par\_2 | ,040 | ,027 |
| par\_3 | ,001 | ,000 | ,001 |
| par\_4 | ,001 | ,000 | ,000 | ,000 |
| par\_5 | -,010 | ,000 | ,000 | ,000 | ,001 |
| par\_6 | -,003 | ,000 | ,000 | ,000 | ,000 | ,000 |
| par\_7 | ,000 | ,000 | ,000 | ,000 | ,000 | ,000 | ,001 |
| par\_8 | ,000 | ,000 | ,000 | ,000 | ,000 | ,000 | ,000 | ,000 |
| par\_9 | ,000 | ,000 | ,000 | ,000 | ,000 | ,000 | ,000 | ,000 | 5133924,250 |
| par\_10 | ,000 | ,000 | ,000 | ,000 | ,000 | ,000 | ,000 | ,000 | -3420,827 | 65,698 |
| par\_11 | ,000 | ,000 | ,000 | ,000 | ,000 | ,000 | ,000 | ,000 | -10263,402 | 32,698 | 140,655 |
| par\_12 | -,003 | -,001 | ,000 | ,000 | ,000 | ,000 | ,000 | ,000 | ,000 | ,000 | ,000 | ,004 |
| par\_13 | ,000 | ,000 | ,000 | ,000 | ,000 | ,000 | ,000 | ,000 | ,000 | ,000 | ,000 | ,000 | ,000 |
| par\_14 | ,000 | ,000 | ,000 | ,000 | ,000 | ,000 | ,000 | ,000 | ,000 | ,000 | ,000 | ,000 | ,000 | ,451 |
| par\_15 | ,000 | ,000 | ,000 | ,000 | ,000 | ,000 | ,000 | ,000 | ,000 | ,000 | ,000 | ,000 | ,000 | ,000 | ,005 |
| par\_16 | ,000 | ,000 | ,000 | ,000 | ,000 | ,000 | ,000 | ,000 | ,000 | ,000 | ,000 | ,000 | ,000 | ,000 | ,000 | ,000 |
| par\_17 | ,000 | ,000 | ,000 | ,000 | ,000 | ,000 | ,000 | ,000 | ,000 | ,000 | ,000 | ,000 | ,000 | ,000 | ,000 | ,000 | ,000 |
| par\_18 | -,019 | -,006 | ,000 | ,000 | ,000 | ,000 | ,000 | ,000 | ,000 | ,000 | ,000 | ,000 | ,000 | ,000 | ,000 | ,000 | ,000 | ,008 |
| par\_19 | -,009 | ,002 | ,001 | ,000 | ,001 | ,000 | ,000 | ,000 | ,000 | ,000 | ,000 | ,000 | ,000 | ,000 | ,000 | ,000 | ,000 | ,000 | ,167 |
| par\_20 | ,003 | ,000 | ,000 | ,000 | ,000 | ,000 | ,000 | ,000 | ,000 | ,000 | ,000 | ,000 | ,000 | ,000 | ,000 | ,000 | ,000 | ,000 | ,000 | ,002 |
| par\_21 | -,014 | -,002 | ,000 | ,000 | ,000 | ,000 | ,000 | ,000 | ,000 | ,000 | ,000 | ,000 | ,000 | ,000 | ,000 | ,000 | ,000 | ,001 | ,006 | ,000 | ,150 |
| par\_22 | ,000 | ,000 | ,000 | ,000 | ,000 | ,000 | ,000 | ,000 | ,000 | ,000 | ,000 | ,000 | ,000 | ,000 | ,000 | ,000 | ,000 | ,000 | -,001 | ,000 | ,000 | ,000 |
| par\_23 | ,000 | ,000 | ,000 | ,000 | ,000 | ,000 | ,000 | ,000 | ,000 | ,000 | ,000 | ,000 | ,000 | ,000 | ,000 | ,000 | ,000 | ,000 | ,000 | ,000 | ,000 | ,000 | ,000 |
| par\_24 | ,000 | ,000 | ,000 | ,000 | ,000 | ,000 | ,000 | ,000 | ,000 | ,000 | ,000 | ,000 | ,000 | ,000 | ,000 | ,000 | ,000 | ,000 | ,000 | ,000 | -,001 | ,000 | ,000 | ,001 |
| par\_25 | ,000 | ,000 | ,000 | ,000 | ,000 | ,000 | ,000 | ,000 | ,000 | ,000 | ,000 | ,000 | ,000 | ,000 | ,000 | ,000 | ,000 | ,000 | ,000 | ,000 | ,000 | ,000 | ,000 | ,000 | ,000 |
| par\_26 | ,000 | ,000 | ,000 | ,000 | ,000 | ,000 | ,000 | ,000 | ,000 | ,000 | ,000 | ,000 | ,000 | ,000 | ,000 | ,000 | ,000 | ,000 | ,000 | ,000 | ,000 | ,000 | ,000 | ,000 | ,000 | ,000 |
| par\_27 | ,000 | ,000 | ,000 | ,000 | ,000 | ,000 | ,000 | ,000 | ,000 | ,000 | ,000 | ,000 | ,000 | ,000 | ,000 | ,000 | ,000 | ,000 | ,000 | ,000 | ,000 | ,000 | ,000 | ,000 | ,000 | ,000 | ,000 |
| par\_28 | ,000 | ,000 | ,000 | ,000 | ,000 | ,000 | ,000 | ,000 | ,000 | ,000 | ,000 | ,000 | ,000 | ,000 | ,000 | ,000 | ,000 | ,000 | ,000 | ,000 | ,000 | ,000 | ,000 | ,000 | ,000 | ,000 | ,000 | 201,189 |
| par\_29 | ,000 | ,000 | ,000 | ,000 | ,000 | ,000 | ,000 | ,000 | ,000 | ,000 | ,000 | ,000 | ,000 | ,000 | ,000 | ,000 | ,000 | ,000 | ,000 | ,000 | ,000 | ,000 | ,000 | ,000 | ,000 | ,000 | ,000 | -,060 | ,003 |
| par\_30 | ,000 | ,000 | ,000 | ,000 | ,000 | ,000 | ,000 | ,000 | ,000 | ,000 | ,000 | ,000 | ,000 | ,000 | ,000 | ,000 | ,000 | ,000 | ,000 | ,000 | ,000 | ,000 | ,000 | ,000 | ,000 | ,000 | ,000 | 42,998 | -,171 | 82,933 |
| par\_31 | ,000 | ,000 | ,000 | ,000 | ,000 | ,000 | ,000 | ,000 | ,000 | ,000 | ,000 | ,000 | ,000 | -,016 | ,000 | ,000 | ,000 | ,000 | ,000 | ,000 | ,000 | ,000 | ,000 | ,000 | ,000 | ,000 | ,000 | ,000 | ,000 | ,000 | ,314 |
| par\_32 | ,000 | ,000 | ,000 | ,000 | ,000 | ,000 | ,000 | ,000 | ,000 | ,000 | ,000 | ,000 | ,000 | ,000 | ,000 | ,000 | ,000 | ,000 | ,000 | ,000 | ,000 | ,000 | ,000 | ,000 | ,000 | ,000 | ,000 | ,000 | ,000 | ,000 | ,000 | ,003 |
| par\_33 | -,024 | -,007 | ,000 | ,000 | ,000 | ,000 | ,000 | ,000 | ,000 | ,000 | ,000 | ,000 | -,001 | ,000 | ,000 | ,000 | ,000 | -,004 | ,000 | ,000 | ,001 | ,000 | ,000 | ,000 | ,000 | ,000 | ,000 | ,000 | ,000 | ,000 | ,000 | ,000 | ,200 |
| par\_34 | -,009 | -,003 | ,000 | ,000 | ,000 | ,000 | ,000 | ,000 | ,000 | ,000 | ,000 | ,000 | ,000 | ,000 | ,000 | ,000 | ,000 | -,002 | ,000 | ,000 | ,000 | ,000 | ,000 | ,000 | ,000 | ,000 | ,000 | ,000 | ,000 | ,000 | ,000 | ,000 | ,083 | ,036 |
| par\_35 | -,004 | -,001 | ,000 | ,000 | ,000 | ,000 | ,000 | ,000 | ,000 | ,000 | ,000 | ,000 | ,000 | ,000 | ,000 | ,000 | ,000 | -,001 | ,000 | ,000 | ,000 | ,000 | ,000 | ,000 | ,000 | ,000 | ,000 | ,000 | ,000 | ,000 | ,000 | ,000 | ,034 | ,014 | ,006 |
| par\_36 | ,181 | ,002 | ,000 | ,000 | -,006 | -,002 | ,000 | ,000 | ,000 | ,000 | ,000 | -,001 | -,003 | ,000 | ,000 | ,000 | ,000 | -,025 | -,007 | ,001 | -,006 | ,000 | ,000 | ,000 | ,000 | ,000 | ,000 | ,000 | ,000 | ,000 | ,000 | ,000 | ,584 | ,245 | ,102 | 2,154 |
| par\_37 | ,011 | ,000 | ,000 | ,000 | ,000 | ,000 | ,000 | ,000 | ,000 | ,000 | ,000 | ,000 | ,000 | ,000 | ,000 | ,000 | ,000 | -,004 | ,000 | ,000 | ,000 | ,000 | ,000 | ,000 | ,000 | ,000 | ,000 | ,000 | ,000 | ,000 | ,000 | ,000 | ,095 | ,040 | ,016 | ,304 | ,051 |
| par\_38 | ,003 | ,000 | ,000 | ,000 | ,000 | ,000 | ,000 | ,000 | ,000 | ,000 | ,000 | ,000 | ,000 | ,000 | ,000 | ,000 | ,000 | -,001 | ,000 | ,000 | ,000 | ,000 | ,000 | ,000 | ,000 | ,000 | ,000 | ,000 | ,000 | ,000 | ,000 | ,000 | ,026 | ,011 | ,004 | ,082 | ,013 | ,004 |
| par\_39 | ,001 | ,010 | ,000 | ,000 | ,000 | ,000 | ,000 | ,000 | ,000 | ,000 | ,000 | ,000 | -,001 | ,000 | ,000 | ,000 | ,000 | -,008 | ,001 | ,000 | -,001 | ,000 | ,000 | ,000 | ,000 | ,000 | ,000 | ,000 | ,000 | ,000 | ,000 | ,000 | ,186 | ,078 | ,032 | ,573 | ,093 | ,025 | ,194 |
| par\_40 | ,000 | ,005 | ,000 | ,000 | ,000 | ,000 | ,001 | ,000 | ,000 | ,000 | ,000 | ,000 | ,000 | ,000 | ,000 | ,000 | ,000 | -,004 | ,001 | ,000 | ,000 | ,000 | ,000 | ,000 | ,000 | ,000 | ,000 | ,000 | ,000 | ,000 | ,000 | ,000 | ,097 | ,041 | ,017 | ,297 | ,048 | ,013 | ,101 | ,054 |
| par\_41 | ,000 | ,001 | ,000 | ,000 | ,000 | ,000 | ,000 | ,000 | ,000 | ,000 | ,000 | ,000 | ,000 | ,000 | ,000 | ,000 | ,000 | -,001 | ,000 | ,000 | ,000 | ,000 | ,000 | ,000 | ,000 | ,000 | ,000 | ,000 | ,000 | ,000 | ,000 | ,000 | ,025 | ,011 | ,004 | ,078 | ,013 | ,003 | ,026 | ,014 | ,004 |
| par\_42 | ,000 | ,000 | ,000 | ,000 | ,000 | ,000 | ,000 | ,000 | 5,703 | -,238 | -,083 | ,000 | ,000 | ,000 | ,000 | ,000 | ,000 | ,000 | ,000 | ,000 | ,000 | ,000 | ,000 | ,000 | ,000 | ,000 | ,000 | ,000 | ,000 | ,000 | ,000 | ,000 | ,000 | ,000 | ,000 | ,000 | ,000 | ,000 | ,000 | ,000 | ,000 | ,003 |
| par\_43 | ,000 | ,000 | ,000 | ,000 | ,000 | ,000 | ,000 | ,000 | 4792475,901 | -1431,271 | -6697,019 | ,000 | ,000 | ,000 | ,000 | ,000 | ,000 | ,000 | ,000 | ,000 | ,000 | ,000 | ,000 | ,000 | ,000 | ,000 | ,000 | ,000 | ,000 | ,000 | ,000 | ,000 | ,000 | ,000 | ,000 | ,000 | ,000 | ,000 | ,000 | ,000 | ,000 | 2,000 | 22424333,716 |
| par\_44 | ,000 | ,000 | ,000 | ,000 | ,000 | ,000 | ,000 | ,000 | 1975522,993 | -7871,436 | -4081,053 | ,000 | ,000 | ,000 | ,000 | ,000 | ,000 | ,000 | ,000 | ,000 | ,000 | ,000 | ,000 | ,000 | ,000 | ,000 | ,000 | ,000 | ,000 | ,000 | ,000 | ,000 | ,000 | ,000 | ,000 | ,000 | ,000 | ,000 | ,000 | ,000 | ,000 | 16,261 | 1024236,687 | 3810341,053 |
| par\_45 | ,000 | ,000 | ,000 | ,000 | ,000 | ,000 | ,000 | ,000 | ,000 | ,000 | ,000 | ,000 | ,000 | ,000 | ,000 | ,000 | ,000 | ,000 | ,000 | ,000 | ,000 | ,000 | ,000 | ,000 | ,000 | ,000 | ,000 | ,000 | ,000 | ,000 | ,000 | ,000 | ,000 | ,000 | ,000 | ,000 | ,000 | ,000 | ,000 | ,000 | ,000 | ,000 | ,000 | ,000 | ,007 |
| par\_46 | ,000 | ,000 | ,000 | ,000 | ,000 | ,000 | ,000 | ,000 | ,000 | ,000 | ,000 | ,000 | ,000 | ,000 | ,000 | ,000 | ,000 | ,000 | ,000 | ,000 | ,000 | ,000 | ,000 | ,000 | ,000 | ,000 | ,000 | ,000 | ,000 | ,000 | ,000 | ,000 | ,000 | ,000 | ,000 | ,000 | ,000 | ,000 | ,000 | ,000 | ,000 | ,000 | ,000 | ,000 | ,000 | 54,347 |
| par\_47 | -,039 | -,012 | ,000 | ,000 | ,000 | ,000 | ,000 | ,000 | ,000 | ,000 | ,000 | ,001 | ,000 | ,000 | ,000 | ,000 | ,000 | ,004 | -,003 | -,001 | ,000 | ,000 | ,000 | ,000 | ,000 | ,000 | ,000 | ,000 | ,000 | ,000 | ,000 | ,000 | ,005 | ,002 | ,001 | -,010 | -,001 | ,000 | -,003 | -,001 | ,000 | ,000 | ,000 | ,000 | ,000 | ,000 | ,015 |
| par\_48 | ,027 | ,008 | -,002 | -,001 | ,000 | ,000 | ,000 | ,000 | ,000 | ,000 | ,000 | ,000 | ,000 | ,000 | ,000 | ,000 | ,000 | -,003 | ,001 | ,000 | -,001 | ,000 | ,000 | ,000 | ,000 | ,000 | ,000 | ,000 | ,000 | ,000 | ,000 | ,000 | -,003 | -,002 | -,001 | ,007 | ,001 | ,000 | ,002 | ,001 | ,000 | ,000 | ,000 | ,000 | ,000 | ,000 | -,008 | ,031 |
| par\_49 | ,599 | ,000 | ,002 | ,000 | -,051 | -,014 | ,000 | ,000 | ,000 | ,000 | ,000 | ,003 | ,000 | ,000 | ,000 | ,000 | ,000 | ,014 | -,022 | -,004 | -,021 | ,001 | ,001 | ,001 | ,000 | ,000 | ,000 | ,000 | ,000 | ,000 | ,000 | ,000 | ,017 | ,009 | ,003 | ,420 | -,028 | -,008 | ,017 | ,009 | ,002 | ,000 | ,000 | ,000 | ,000 | ,000 | ,002 | -,026 | 7,437 |
| par\_50 | ,000 | -,010 | ,000 | ,000 | ,000 | ,000 | -,002 | ,000 | ,000 | ,000 | ,000 | ,000 | ,000 | ,000 | ,000 | ,000 | ,000 | ,001 | -,001 | ,000 | ,001 | ,000 | ,000 | ,000 | ,000 | ,000 | ,000 | ,000 | ,000 | ,000 | ,000 | ,000 | ,002 | ,001 | ,000 | ,005 | ,001 | ,000 | -,004 | -,003 | -,001 | ,000 | ,000 | ,000 | ,000 | ,000 | ,000 | -,002 | -,020 | ,043 |
| par\_51 | ,004 | ,001 | ,002 | ,001 | ,000 | ,000 | ,000 | ,000 | ,000 | ,000 | ,000 | ,000 | ,000 | ,000 | ,000 | ,000 | ,000 | ,000 | ,001 | ,001 | ,000 | ,000 | ,000 | ,000 | ,000 | ,000 | ,000 | ,000 | ,000 | ,000 | ,000 | ,000 | -,001 | ,001 | ,000 | ,001 | ,000 | ,000 | ,000 | ,000 | ,000 | ,000 | ,000 | ,000 | ,000 | ,000 | -,001 | -,007 | ,001 | ,000 | ,009 |
| par\_52 | ,000 | ,000 | ,000 | ,000 | ,000 | ,000 | ,000 | ,000 | ,000 | ,000 | ,000 | ,000 | ,000 | ,000 | ,000 | ,000 | ,000 | ,000 | ,000 | ,000 | ,000 | ,000 | ,000 | ,000 | ,000 | ,000 | ,000 | ,000 | ,000 | ,000 | ,000 | ,000 | ,000 | ,000 | ,000 | ,000 | ,000 | ,000 | ,000 | ,000 | ,000 | ,000 | ,000 | ,000 | ,000 | ,000 | ,000 | ,000 | ,000 | ,000 | ,000 | ,000 |
| par\_53 | -,001 | ,000 | ,000 | ,000 | ,000 | ,000 | ,000 | ,000 | ,000 | ,000 | ,000 | ,000 | ,000 | ,000 | ,000 | ,000 | ,000 | ,000 | ,000 | ,000 | ,000 | ,000 | ,000 | ,000 | ,000 | ,000 | ,000 | ,000 | ,000 | ,000 | ,000 | ,000 | ,000 | ,000 | ,000 | ,000 | ,000 | ,000 | ,000 | ,000 | ,000 | ,000 | ,000 | ,000 | ,000 | ,000 | ,000 | ,001 | ,000 | ,000 | -,001 | ,000 | ,001 |
| par\_54 | -,462 | -,002 | ,000 | ,000 | ,026 | ,007 | ,000 | ,000 | ,000 | ,000 | ,000 | ,000 | ,000 | ,000 | ,000 | ,000 | ,000 | ,001 | -,041 | ,000 | ,039 | -,001 | ,000 | -,001 | ,000 | ,000 | ,000 | ,000 | ,000 | ,000 | ,000 | ,000 | ,001 | ,000 | ,000 | -,278 | ,005 | ,001 | ,000 | ,000 | ,000 | ,000 | ,000 | ,000 | ,000 | ,000 | ,001 | ,000 | -2,600 | ,001 | ,001 | ,000 | ,000 | 29,693 |
| par\_55 | ,002 | ,000 | ,000 | ,000 | ,000 | ,000 | ,000 | ,000 | ,000 | ,000 | ,000 | ,000 | ,000 | ,000 | ,000 | ,000 | ,000 | ,000 | -,001 | ,000 | ,000 | ,000 | ,000 | ,000 | ,000 | ,000 | ,000 | ,000 | ,000 | ,000 | ,000 | ,000 | ,000 | ,000 | ,000 | ,001 | -,001 | ,000 | ,000 | ,000 | ,000 | ,000 | ,000 | ,000 | ,000 | ,000 | ,000 | ,000 | ,001 | ,000 | ,000 | ,000 | ,000 | -,010 | ,002 |
| par\_56 | ,000 | ,000 | ,000 | ,000 | ,000 | ,000 | ,000 | ,000 | ,000 | ,000 | ,000 | ,000 | ,000 | ,000 | ,000 | ,000 | ,000 | ,000 | ,000 | ,000 | ,000 | ,000 | ,000 | ,000 | ,000 | ,000 | ,000 | ,000 | ,000 | ,000 | ,000 | ,000 | ,000 | ,000 | ,000 | ,000 | ,000 | ,000 | ,000 | ,000 | ,000 | ,000 | ,000 | ,000 | ,000 | ,000 | ,000 | ,000 | ,000 | ,000 | ,000 | ,000 | ,000 | -,001 | ,000 | ,000 |
| par\_57 | ,000 | ,000 | ,000 | ,000 | ,000 | ,000 | ,000 | ,000 | ,000 | ,000 | ,000 | ,000 | ,000 | ,000 | ,000 | ,000 | ,000 | ,000 | ,000 | ,000 | ,000 | ,000 | ,000 | ,000 | ,000 | ,000 | ,000 | ,000 | ,000 | ,000 | ,000 | ,000 | ,000 | ,000 | ,000 | ,000 | ,000 | ,000 | ,000 | ,000 | ,000 | ,000 | ,000 | ,000 | ,000 | ,000 | ,000 | ,000 | ,000 | ,002 | ,000 | ,000 | ,000 | ,000 | ,000 | ,000 | ,001 |
| par\_58 | ,000 | ,000 | ,000 | ,000 | ,000 | ,000 | ,002 | ,000 | ,000 | ,000 | ,000 | ,000 | ,000 | ,000 | ,000 | ,000 | ,000 | ,000 | ,000 | ,001 | ,001 | ,000 | ,000 | ,000 | ,000 | ,000 | ,000 | ,000 | ,000 | ,000 | ,000 | ,000 | ,000 | ,000 | ,000 | ,000 | ,000 | ,000 | ,000 | ,001 | ,000 | ,000 | ,000 | ,000 | ,000 | ,000 | ,000 | ,000 | -,001 | -,008 | ,000 | ,000 | ,000 | ,001 | ,000 | ,000 | -,002 | ,009 |
| par\_59 | ,000 | ,000 | ,000 | ,000 | ,000 | ,000 | ,000 | ,000 | ,000 | ,000 | ,000 | ,000 | ,000 | ,000 | ,000 | ,000 | ,000 | ,000 | ,000 | ,000 | ,000 | ,000 | ,000 | ,000 | ,000 | ,000 | ,000 | ,000 | ,000 | ,000 | ,000 | ,000 | ,000 | ,000 | ,000 | ,000 | ,000 | ,000 | ,000 | ,000 | ,000 | ,000 | ,000 | ,000 | ,000 | ,000 | ,000 | ,000 | ,000 | ,000 | ,000 | ,000 | ,000 | ,000 | ,000 | ,000 | ,000 | ,000 | ,000 |

##### Correlations of Estimates (Default model)

|  | par\_1 | par\_2 | par\_3 | par\_4 | par\_5 | par\_6 | par\_7 | par\_8 | par\_9 | par\_10 | par\_11 | par\_12 | par\_13 | par\_14 | par\_15 | par\_16 | par\_17 | par\_18 | par\_19 | par\_20 | par\_21 | par\_22 | par\_23 | par\_24 | par\_25 | par\_26 | par\_27 | par\_28 | par\_29 | par\_30 | par\_31 | par\_32 | par\_33 | par\_34 | par\_35 | par\_36 | par\_37 | par\_38 | par\_39 | par\_40 | par\_41 | par\_42 | par\_43 | par\_44 | par\_45 | par\_46 | par\_47 | par\_48 | par\_49 | par\_50 | par\_51 | par\_52 | par\_53 | par\_54 | par\_55 | par\_56 | par\_57 | par\_58 | par\_59 |
| --- | --- | --- | --- | --- | --- | --- | --- | --- | --- | --- | --- | --- | --- | --- | --- | --- | --- | --- | --- | --- | --- | --- | --- | --- | --- | --- | --- | --- | --- | --- | --- | --- | --- | --- | --- | --- | --- | --- | --- | --- | --- | --- | --- | --- | --- | --- | --- | --- | --- | --- | --- | --- | --- | --- | --- | --- | --- | --- | --- |
| par\_1 | 1,000 |
| par\_2 | ,371 | 1,000 |
| par\_3 | ,065 | ,091 | 1,000 |
| par\_4 | ,071 | ,058 | ,394 | 1,000 |
| par\_5 | -,585 | -,006 | -,048 | -,028 | 1,000 |
| par\_6 | -,590 | -,013 | ,002 | ,027 | ,722 | 1,000 |
| par\_7 | ,000 | -,031 | ,044 | ,005 | ,010 | -,008 | 1,000 |
| par\_8 | -,007 | -,037 | ,007 | ,009 | ,003 | -,016 | ,361 | 1,000 |
| par\_9 | ,000 | ,000 | ,000 | ,000 | ,000 | ,000 | ,000 | ,000 | 1,000 |
| par\_10 | ,000 | ,000 | ,000 | ,000 | ,000 | ,000 | ,000 | ,000 | -,186 | 1,000 |
| par\_11 | ,000 | ,000 | ,000 | ,000 | ,000 | ,000 | ,000 | ,000 | -,382 | ,340 | 1,000 |
| par\_12 | -,089 | -,107 | -,018 | -,014 | ,002 | ,003 | ,003 | ,003 | ,000 | ,000 | ,000 | 1,000 |
| par\_13 | -,114 | -,136 | -,023 | -,019 | ,003 | ,004 | ,004 | ,004 | ,000 | ,000 | ,000 | ,021 | 1,000 |
| par\_14 | ,000 | ,000 | ,000 | ,000 | ,000 | ,000 | ,000 | ,000 | ,000 | ,000 | ,000 | ,000 | ,000 | 1,000 |
| par\_15 | ,000 | ,000 | ,000 | ,000 | ,000 | ,000 | ,000 | ,000 | ,000 | ,000 | ,000 | ,000 | ,000 | ,000 | 1,000 |
| par\_16 | ,094 | ,113 | ,019 | ,015 | -,002 | -,003 | -,003 | -,004 | ,000 | ,000 | ,000 | -,017 | -,022 | ,000 | ,000 | 1,000 |
| par\_17 | ,053 | ,063 | ,010 | ,009 | -,001 | -,002 | -,002 | -,002 | ,000 | ,000 | ,000 | -,010 | -,013 | ,000 | ,000 | -,312 | 1,000 |
| par\_18 | -,333 | -,398 | -,066 | -,054 | ,008 | ,012 | ,010 | ,013 | ,000 | ,000 | ,000 | -,088 | -,101 | ,000 | ,000 | ,245 | -,076 | 1,000 |
| par\_19 | -,035 | ,032 | ,046 | ,020 | ,071 | ,037 | ,006 | ,006 | ,000 | ,000 | ,000 | -,003 | -,003 | ,000 | ,000 | ,003 | ,002 | -,010 | 1,000 |
| par\_20 | ,097 | -,010 | ,090 | ,102 | ,002 | -,001 | ,093 | ,099 | ,000 | ,000 | ,000 | -,010 | -,013 | ,000 | ,000 | ,011 | ,006 | -,037 | ,022 | 1,000 |
| par\_21 | -,057 | -,032 | ,007 | ,004 | ,044 | ,057 | ,027 | ,031 | ,000 | ,000 | ,000 | ,005 | ,006 | ,000 | ,000 | -,005 | -,003 | ,017 | ,039 | ,007 | 1,000 |
| par\_22 | ,005 | ,012 | -,091 | -,078 | -,024 | -,015 | -,009 | -,006 | ,000 | ,000 | ,000 | ,001 | ,001 | ,000 | ,000 | -,001 | ,000 | ,002 | -,098 | -,044 | -,014 | 1,000 |
| par\_23 | -,036 | ,004 | -,037 | -,059 | -,002 | -,001 | -,049 | -,062 | ,000 | ,000 | ,000 | ,004 | ,005 | ,000 | ,000 | -,004 | -,002 | ,014 | -,016 | -,386 | -,025 | ,044 | 1,000 |
| par\_24 | -,004 | -,006 | -,012 | -,010 | -,006 | -,011 | -,044 | -,040 | ,000 | ,000 | ,000 | ,001 | ,002 | ,000 | ,000 | -,002 | -,001 | ,005 | -,015 | -,080 | -,116 | ,093 | -,046 | 1,000 |
| par\_25 | ,003 | ,016 | -,021 | -,022 | -,005 | -,012 | -,007 | -,006 | ,000 | ,000 | ,000 | -,001 | -,001 | ,000 | ,000 | ,001 | ,000 | -,002 | -,072 | -,040 | -,014 | -,027 | ,018 | ,016 | 1,000 |
| par\_26 | -,024 | ,002 | -,044 | -,027 | -,001 | ,000 | -,052 | -,034 | ,000 | ,000 | ,000 | ,003 | ,003 | ,000 | ,000 | -,003 | -,002 | ,010 | -,012 | -,250 | -,014 | ,016 | ,096 | ,024 | -,027 | 1,000 |
| par\_27 | ,002 | -,003 | -,008 | -,005 | -,011 | -,008 | -,052 | -,044 | ,000 | ,000 | ,000 | ,001 | ,001 | ,000 | ,000 | -,001 | ,000 | ,003 | -,013 | -,033 | -,086 | ,014 | ,017 | ,002 | ,014 | ,124 | 1,000 |
| par\_28 | ,000 | ,000 | ,000 | ,000 | ,000 | ,000 | ,000 | ,000 | ,000 | ,000 | ,000 | ,000 | ,000 | ,000 | ,000 | ,000 | ,000 | ,000 | ,000 | ,000 | ,000 | ,000 | ,000 | ,000 | ,000 | ,000 | ,000 | 1,000 |
| par\_29 | ,000 | ,000 | ,000 | ,000 | ,000 | ,000 | ,000 | ,000 | ,000 | ,000 | ,000 | ,000 | ,000 | ,000 | ,000 | ,000 | ,000 | ,000 | ,000 | ,000 | ,000 | ,000 | ,000 | ,000 | ,000 | ,000 | ,000 | -,085 | 1,000 |
| par\_30 | ,000 | ,000 | ,000 | ,000 | ,000 | ,000 | ,000 | ,000 | ,000 | ,000 | ,000 | ,000 | ,000 | ,000 | ,000 | ,000 | ,000 | ,000 | ,000 | ,000 | ,000 | ,000 | ,000 | ,000 | ,000 | ,000 | ,000 | ,333 | -,376 | 1,000 |
| par\_31 | ,000 | ,000 | ,000 | ,000 | ,000 | ,000 | ,000 | ,000 | ,000 | ,000 | ,000 | ,000 | ,000 | -,044 | ,000 | ,000 | ,000 | ,000 | ,000 | ,000 | ,000 | ,000 | ,000 | ,000 | ,000 | ,000 | ,000 | ,000 | ,000 | ,000 | 1,000 |
| par\_32 | ,000 | ,000 | ,000 | ,000 | ,000 | ,000 | ,000 | ,000 | ,000 | ,000 | ,000 | ,000 | ,000 | ,000 | -,044 | ,000 | ,000 | ,000 | ,000 | ,000 | ,000 | ,000 | ,000 | ,000 | ,000 | ,000 | ,000 | ,000 | ,000 | ,000 | ,000 | 1,000 |
| par\_33 | -,083 | -,099 | -,016 | -,013 | ,002 | ,003 | ,003 | ,003 | ,000 | ,000 | ,000 | ,015 | -,263 | ,000 | ,000 | -,529 | -,573 | -,109 | -,002 | -,009 | ,004 | ,001 | ,004 | ,001 | -,001 | ,002 | ,001 | ,000 | ,000 | ,000 | ,000 | ,000 | 1,000 |
| par\_34 | -,075 | -,089 | ,081 | ,025 | -,003 | ,003 | ,007 | ,004 | ,000 | ,000 | ,000 | ,014 | -,261 | ,000 | ,000 | -,520 | -,564 | -,114 | ,002 | ,000 | ,005 | -,008 | ,000 | ,000 | -,003 | -,002 | ,000 | ,000 | ,000 | ,000 | ,000 | ,000 | ,981 | 1,000 |
| par\_35 | -,073 | -,091 | ,026 | ,093 | -,001 | ,006 | ,003 | ,004 | ,000 | ,000 | ,000 | ,014 | -,260 | ,000 | ,000 | -,517 | -,561 | -,113 | ,000 | ,002 | ,005 | -,008 | -,003 | ,000 | -,003 | -,001 | ,000 | ,000 | ,000 | ,000 | ,000 | ,000 | ,975 | ,965 | 1,000 |
| par\_36 | ,192 | ,007 | ,002 | ,006 | -,156 | -,156 | ,002 | ,001 | ,000 | ,000 | ,000 | -,009 | -,277 | ,000 | ,000 | -,470 | -,522 | -,192 | -,012 | ,017 | -,011 | ,002 | -,006 | ,000 | ,000 | -,004 | ,001 | ,000 | ,000 | ,000 | ,000 | ,000 | ,891 | ,880 | ,875 | 1,000 |
| par\_37 | ,079 | ,006 | -,008 | ,001 | ,046 | -,012 | ,005 | ,002 | ,000 | ,000 | ,000 | -,010 | -,291 | ,000 | ,000 | -,497 | -,551 | -,201 | ,003 | ,019 | -,003 | -,003 | -,007 | -,001 | -,001 | -,005 | -,001 | ,000 | ,000 | ,000 | ,000 | ,000 | ,941 | ,925 | ,923 | ,918 | 1,000 |
| par\_38 | ,078 | ,005 | ,003 | ,012 | -,012 | ,046 | ,001 | -,002 | ,000 | ,000 | ,000 | -,009 | -,291 | ,000 | ,000 | -,497 | -,551 | -,200 | -,005 | ,018 | ,000 | -,001 | -,007 | -,002 | -,002 | -,004 | -,001 | ,000 | ,000 | ,000 | ,000 | ,000 | ,942 | ,929 | ,928 | ,918 | ,965 | 1,000 |
| par\_39 | ,003 | ,132 | ,005 | ,000 | ,001 | ,000 | -,005 | -,005 | ,000 | ,000 | ,000 | -,009 | -,292 | ,000 | ,000 | -,499 | -,553 | -,200 | ,005 | -,011 | -,003 | ,003 | ,004 | ,000 | ,003 | ,003 | ,000 | ,000 | ,000 | ,000 | ,000 | ,000 | ,944 | ,933 | ,927 | ,886 | ,935 | ,935 | 1,000 |
| par\_40 | ,003 | ,127 | ,008 | ,000 | ,001 | -,001 | ,074 | ,023 | ,000 | ,000 | ,000 | -,009 | -,287 | ,000 | ,000 | -,492 | -,546 | -,196 | ,005 | -,004 | -,001 | ,003 | ,001 | -,004 | ,002 | -,001 | -,004 | ,000 | ,000 | ,000 | ,000 | ,000 | ,932 | ,924 | ,914 | ,874 | ,923 | ,922 | ,985 | 1,000 |
| par\_41 | ,002 | ,125 | ,005 | ,001 | ,001 | -,001 | ,029 | ,087 | ,000 | ,000 | ,000 | -,009 | -,285 | ,000 | ,000 | -,488 | -,541 | -,194 | ,005 | -,002 | ,000 | ,003 | -,001 | -,004 | ,002 | ,000 | -,004 | ,000 | ,000 | ,000 | ,000 | ,000 | ,924 | ,912 | ,907 | ,866 | ,913 | ,911 | ,975 | ,964 | 1,000 |
| par\_42 | ,000 | ,000 | ,000 | ,000 | ,000 | ,000 | ,000 | ,000 | ,043 | -,498 | -,119 | ,000 | ,000 | ,000 | ,000 | ,000 | ,000 | ,000 | ,000 | ,000 | ,000 | ,000 | ,000 | ,000 | ,000 | ,000 | ,000 | ,000 | ,000 | ,000 | ,000 | ,000 | ,000 | ,000 | ,000 | ,000 | ,000 | ,000 | ,000 | ,000 | ,000 | 1,000 |
| par\_43 | ,000 | ,000 | ,000 | ,000 | ,000 | ,000 | ,000 | ,000 | ,447 | -,037 | -,119 | ,000 | ,000 | ,000 | ,000 | ,000 | ,000 | ,000 | ,000 | ,000 | ,000 | ,000 | ,000 | ,000 | ,000 | ,000 | ,000 | ,000 | ,000 | ,000 | ,000 | ,000 | ,000 | ,000 | ,000 | ,000 | ,000 | ,000 | ,000 | ,000 | ,000 | ,007 | 1,000 |
| par\_44 | ,000 | ,000 | ,000 | ,000 | ,000 | ,000 | ,000 | ,000 | ,447 | -,498 | -,176 | ,000 | ,000 | ,000 | ,000 | ,000 | ,000 | ,000 | ,000 | ,000 | ,000 | ,000 | ,000 | ,000 | ,000 | ,000 | ,000 | ,000 | ,000 | ,000 | ,000 | ,000 | ,000 | ,000 | ,000 | ,000 | ,000 | ,000 | ,000 | ,000 | ,000 | ,141 | ,111 | 1,000 |
| par\_45 | ,000 | ,000 | ,000 | ,000 | ,000 | ,000 | ,000 | ,000 | ,000 | ,000 | ,000 | ,000 | ,000 | ,000 | ,000 | ,000 | ,000 | ,000 | ,000 | ,000 | ,000 | ,000 | ,000 | ,000 | ,000 | ,000 | ,000 | ,000 | ,000 | ,000 | ,000 | ,000 | ,000 | ,000 | ,000 | ,000 | ,000 | ,000 | ,000 | ,000 | ,000 | ,000 | ,000 | ,000 | 1,000 |
| par\_46 | ,000 | ,000 | ,000 | ,000 | ,000 | ,000 | ,000 | ,000 | ,000 | ,000 | ,000 | ,000 | ,000 | ,000 | ,000 | ,000 | ,000 | ,000 | ,000 | ,000 | ,000 | ,000 | ,000 | ,000 | ,000 | ,000 | ,000 | ,000 | ,000 | ,000 | ,000 | ,000 | ,000 | ,000 | ,000 | ,000 | ,000 | ,000 | ,000 | ,000 | ,000 | ,000 | ,000 | ,000 | ,000 | 1,000 |
| par\_47 | -,488 | -,583 | -,106 | -,072 | ,013 | ,020 | ,012 | ,007 | ,000 | ,000 | ,000 | ,090 | ,115 | ,000 | ,000 | -,095 | -,053 | ,336 | -,050 | -,145 | -,007 | -,004 | ,056 | ,019 | -,009 | ,037 | ,010 | ,000 | ,000 | ,000 | ,000 | ,000 | ,083 | ,072 | ,074 | -,053 | -,053 | -,052 | -,051 | -,049 | -,049 | ,000 | ,000 | ,000 | ,000 | ,000 | 1,000 |
| par\_48 | ,243 | ,286 | -,333 | -,312 | ,011 | -,008 | -,009 | -,003 | ,000 | ,000 | ,000 | -,043 | -,054 | ,000 | ,000 | ,045 | ,025 | -,159 | ,010 | ,021 | -,009 | ,076 | ,009 | -,007 | ,030 | ,010 | -,004 | ,000 | ,000 | ,000 | ,000 | ,000 | -,039 | -,071 | -,072 | ,028 | ,032 | ,028 | ,027 | ,025 | ,026 | ,000 | ,000 | ,000 | ,000 | ,000 | -,375 | 1,000 |
| par\_49 | ,341 | ,000 | ,026 | -,009 | -,742 | -,743 | -,003 | ,007 | ,000 | ,000 | ,000 | ,015 | ,020 | ,000 | ,000 | -,016 | -,009 | ,057 | -,019 | -,032 | -,020 | ,024 | ,013 | ,009 | ,020 | ,008 | ,007 | ,000 | ,000 | ,000 | ,000 | ,000 | ,014 | ,017 | ,013 | ,105 | -,045 | -,045 | ,014 | ,014 | ,015 | ,000 | ,000 | ,000 | ,000 | ,000 | ,007 | -,054 | 1,000 |
| par\_50 | ,003 | -,280 | -,010 | ,008 | -,001 | ,004 | -,305 | -,238 | ,000 | ,000 | ,000 | ,019 | ,024 | ,000 | ,000 | -,020 | -,011 | ,069 | -,008 | ,025 | ,011 | -,010 | ,010 | ,028 | -,007 | ,013 | ,034 | ,000 | ,000 | ,000 | ,000 | ,000 | ,017 | ,016 | ,018 | ,017 | ,017 | ,019 | -,047 | -,070 | -,068 | ,000 | ,000 | ,000 | ,000 | ,000 | ,008 | -,053 | -,035 | 1,000 |
| par\_51 | ,062 | ,059 | ,606 | ,529 | -,019 | ,005 | ,025 | ,011 | ,000 | ,000 | ,000 | -,014 | -,018 | ,000 | ,000 | ,015 | ,008 | -,053 | ,019 | ,128 | ,006 | -,132 | -,082 | -,016 | -,037 | -,062 | -,009 | ,000 | ,000 | ,000 | ,000 | ,000 | -,013 | ,046 | ,043 | ,005 | ,001 | ,006 | ,001 | ,002 | ,002 | ,000 | ,000 | ,000 | ,000 | ,000 | -,089 | -,431 | ,002 | ,008 | 1,000 |
| par\_52 | -,029 | -,023 | -,206 | -,270 | ,007 | -,005 | -,010 | -,008 | ,000 | ,000 | ,000 | ,006 | ,008 | ,000 | ,000 | -,006 | -,004 | ,022 | -,020 | -,079 | -,004 | ,047 | ,045 | ,009 | ,167 | ,050 | ,007 | ,000 | ,000 | ,000 | ,000 | ,000 | ,005 | -,015 | -,023 | -,003 | -,001 | -,004 | ,000 | -,001 | -,001 | ,000 | ,000 | ,000 | ,000 | ,000 | ,039 | ,146 | ,002 | -,004 | -,341 | 1,000 |
| par\_53 | -,036 | -,036 | -,389 | -,256 | ,012 | -,004 | -,020 | -,008 | ,000 | ,000 | ,000 | ,008 | ,011 | ,000 | ,000 | -,009 | -,005 | ,032 | -,037 | -,095 | -,006 | -,044 | ,155 | ,001 | ,019 | ,042 | ,007 | ,000 | ,000 | ,000 | ,000 | ,000 | ,008 | -,030 | -,020 | -,002 | ,000 | -,003 | ,000 | -,002 | -,001 | ,000 | ,000 | ,000 | ,000 | ,000 | ,057 | ,206 | -,002 | -,003 | -,484 | ,157 | 1,000 |
| par\_54 | -,131 | -,002 | -,003 | ,001 | ,192 | ,191 | ,001 | ,000 | ,000 | ,000 | ,000 | ,001 | ,001 | ,000 | ,000 | -,001 | ,000 | ,002 | -,019 | ,002 | ,019 | -,010 | -,002 | -,005 | -,005 | -,001 | -,005 | ,000 | ,000 | ,000 | ,000 | ,000 | ,001 | ,000 | ,001 | -,035 | ,004 | ,004 | ,000 | ,000 | ,000 | ,000 | ,000 | ,000 | ,000 | ,000 | ,002 | ,000 | -,175 | ,001 | ,002 | -,001 | -,001 | 1,000 |
| par\_55 | ,061 | -,004 | ,034 | ,036 | -,312 | ,123 | -,014 | -,014 | ,000 | ,000 | ,000 | ,001 | ,001 | ,000 | ,000 | -,001 | ,000 | ,002 | -,056 | -,006 | -,005 | -,089 | ,000 | ,035 | -,007 | ,002 | ,005 | ,000 | ,000 | ,000 | ,000 | ,000 | ,001 | ,004 | ,004 | ,017 | -,048 | ,044 | ,000 | -,001 | -,002 | ,000 | ,000 | ,000 | ,000 | ,000 | ,005 | -,010 | ,005 | ,002 | ,009 | -,006 | -,001 | -,038 | 1,000 |
| par\_56 | ,066 | ,004 | -,031 | -,039 | ,126 | -,308 | ,012 | ,013 | ,000 | ,000 | ,000 | -,001 | -,001 | ,000 | ,000 | ,001 | ,001 | -,004 | ,015 | ,002 | -,032 | -,012 | ,000 | ,008 | ,132 | -,006 | -,046 | ,000 | ,000 | ,000 | ,000 | ,000 | -,001 | -,004 | -,005 | ,017 | ,044 | -,047 | ,000 | ,001 | ,001 | ,000 | ,000 | ,000 | ,000 | ,000 | -,004 | ,008 | ,001 | -,002 | -,010 | ,017 | ,008 | -,036 | -,431 | 1,000 |
| par\_57 | -,008 | ,021 | -,021 | -,009 | -,004 | ,003 | -,417 | -,322 | ,000 | ,000 | ,000 | -,001 | -,001 | ,000 | ,000 | ,001 | ,000 | -,003 | -,006 | -,116 | -,031 | ,011 | ,166 | ,084 | ,008 | ,056 | ,050 | ,000 | ,000 | ,000 | ,000 | ,000 | -,001 | -,003 | -,002 | -,003 | -,004 | -,002 | ,004 | -,029 | -,026 | ,000 | ,000 | ,000 | ,000 | ,000 | ,011 | -,004 | ,004 | ,248 | -,019 | ,010 | ,021 | -,001 | ,006 | -,003 | 1,000 |
| par\_58 | ,008 | -,032 | ,027 | ,011 | ,005 | -,005 | ,637 | ,526 | ,000 | ,000 | ,000 | ,002 | ,002 | ,000 | ,000 | -,002 | -,001 | ,006 | ,007 | ,136 | ,038 | -,010 | -,096 | -,072 | -,010 | -,077 | -,080 | ,000 | ,000 | ,000 | ,000 | ,000 | ,002 | ,004 | ,003 | ,004 | ,005 | ,003 | -,006 | ,044 | ,043 | ,000 | ,000 | ,000 | ,000 | ,000 | -,011 | ,005 | -,004 | -,411 | ,021 | -,013 | -,018 | ,001 | -,007 | ,004 | -,609 | 1,000 |
| par\_59 | -,005 | ,014 | -,012 | -,007 | -,002 | ,002 | -,251 | -,235 | ,000 | ,000 | ,000 | ,000 | -,001 | ,000 | ,000 | ,000 | ,000 | -,002 | -,004 | -,081 | -,022 | ,005 | ,049 | ,031 | ,004 | ,058 | -,022 | ,000 | ,000 | ,000 | ,000 | ,000 | ,000 | -,002 | -,001 | -,002 | -,002 | -,001 | ,003 | -,017 | -,019 | ,000 | ,000 | ,000 | ,000 | ,000 | ,008 | -,003 | ,002 | ,160 | -,013 | ,008 | ,009 | -,001 | ,003 | -,001 | ,240 | -,394 | 1,000 |

##### Bootstrap (Default model)

##### Summary of Bootstrap Iterations (Default model)

##### (Default model)

| Iterations | Method 0 | Method 1 | Method 2 |
| --- | --- | --- | --- |
| 1 | 0 | 0 | 0 |
| 2 | 0 | 0 | 0 |
| 3 | 0 | 0 | 3 |
| 4 | 0 | 0 | 19 |
| 5 | 0 | 0 | 1 |
| 6 | 0 | 0 | 0 |
| 7 | 0 | 0 | 0 |
| 8 | 0 | 6 | 0 |
| 9 | 0 | 29 | 0 |
| 10 | 0 | 22 | 0 |
| 11 | 0 | 14 | 0 |
| 12 | 0 | 3 | 0 |
| 13 | 0 | 2 | 0 |
| 14 | 0 | 0 | 0 |
| 15 | 0 | 1 | 0 |
| 16 | 0 | 0 | 0 |
| 17 | 0 | 0 | 0 |
| 18 | 0 | 0 | 0 |
| 19 | 0 | 0 | 0 |
| Total | 0 | 77 | 23 |

0 bootstrap samples were unused because of a singular covariance matrix.

0 bootstrap samples were unused because a solution was not found.

100 usable bootstrap samples were obtained.

##### Model Fit Summary

##### CMIN

| Model | NPAR | CMIN | DF | P | CMIN/DF |
| --- | --- | --- | --- | --- | --- |
| Default model | 59 | 72,640 | 60 | ,127 | 1,211 |
| Saturated model | 119 | ,000 | 0 |
| Independence model | 28 | 1337,713 | 91 | ,000 | 14,700 |

##### Baseline Comparisons

| Model | NFI Delta1 | RFI rho1 | IFI Delta2 | TLI rho2 | CFI |
| --- | --- | --- | --- | --- | --- |
| Default model | ,946 | ,918 | ,990 | ,985 | ,990 |
| Saturated model | 1,000 |  | 1,000 |  | 1,000 |
| Independence model | ,000 | ,000 | ,000 | ,000 | ,000 |

##### Parsimony-Adjusted Measures

| Model | PRATIO | PNFI | PCFI |
| --- | --- | --- | --- |
| Default model | ,659 | ,624 | ,653 |
| Saturated model | ,000 | ,000 | ,000 |
| Independence model | 1,000 | ,000 | ,000 |

##### NCP

| Model | NCP | LO 90 | HI 90 |
| --- | --- | --- | --- |
| Default model | 12,640 | ,000 | 38,294 |
| Saturated model | ,000 | ,000 | ,000 |
| Independence model | 1246,713 | 1132,122 | 1368,714 |

##### FMIN

| Model | FMIN | F0 | LO 90 | HI 90 |
| --- | --- | --- | --- | --- |
| Default model | ,262 | ,046 | ,000 | ,138 |
| Saturated model | ,000 | ,000 | ,000 | ,000 |
| Independence model | 4,829 | 4,501 | 4,087 | 4,941 |

##### RMSEA

| Model | RMSEA | LO 90 | HI 90 | PCLOSE |
| --- | --- | --- | --- | --- |
| Default model | ,028 | ,000 | ,048 | ,967 |
| Independence model | ,222 | ,212 | ,233 | ,000 |

##### AIC

| Model | AIC | BCC | BIC | CAIC |
| --- | --- | --- | --- | --- |
| Default model | 190,640 | 197,396 |
| Saturated model | 238,000 | 251,626 |
| Independence model | 1393,713 | 1396,919 |

##### ECVI

| Model | ECVI | LO 90 | HI 90 | MECVI |
| --- | --- | --- | --- | --- |
| Default model | ,688 | ,643 | ,781 | ,713 |
| Saturated model | ,859 | ,859 | ,859 | ,908 |
| Independence model | 5,031 | 4,618 | 5,472 | 5,043 |

##### HOELTER

| Model | HOELTER .05 | HOELTER .01 |
| --- | --- | --- |
| Default model | 302 | 338 |
| Independence model | 24 | 26 |

##### Execution time summary

|  |  |
| --- | --- |
| Minimization: | ,016 |
| Miscellaneous: | 1,325 |
| Bootstrap: | ,204 |
| Total: | 1,545 |
